# Supplementary material for: Molecular and functional properties of human Plasmodium falciparum CSP C‐terminus antibodies
Source: EMBO Mol Med. 2023 Apr 21;15(6):e17454. doi: 10.15252/emmm.202317454 (PMC10245032; doi:10.15252/emmm.202317454)
Supplement: Supplementary file 1 — Appendix [file EMMM-15-e17454-s006.pdf]

Molecular and functional properties of human *Plasmodium falciparum* CSP C-terminus antibodies

Opeyemi Ernest Oludada[1,2], Giulia Costa[3], Clare Burn Aschner[4], Anna S. Obraztsova[1,2], Katherine Prieto[4], Caterina Canetta[1], Stephen L. Hoffman[5], Peter G. Kremsner[6,7], Benjamin, Mordmüller[6,8], Rajagopal Murugan[1*], Jean-Philippe Julien[4,9*], Elena A. Levashina[3*], and Hedda Wardemann[1*]

[1]B Cell Immunology, German Cancer Research Center, Heidelberg, Germany

[2]Biosciences Faculty, University of Heidelberg, Germany

[3]Max Planck Institute for Infection Biology, Berlin, Germany

[4]The Hospital for Sick Children Research Institute, Toronto, Canada

[5]Sanaria Inc., Rockville, MD 20850, USA

[6]Institute of Tropical Medicine, Tübingen, Germany

[7]Centre de Recherches de Lambaréné (CERMEL), Lambaréné, Gabon

[8]Radboud University Medical Center, Nijmegen, The Netherlands

[9]Departments of Biochemistry and Immunology, University of Toronto, Toronto, Canada

*equal contribution

Correspondence: Hedda Wardemann (h.wardemann@dkfz.de), Elena A. Levashina (levashina@mpiib-berlin.mpg.de), Jean-Philippe Julien (jean-philippe.julien@sickkids.ca)

TABLE OF CONTENT

Appendix Table S1: Amino acid (aa) sequences of PfCSP antigens used in ELISAs

Appendix Table S2: Sequenced and cloned antibodies per time-point

Appendix Table S3: Epitope specificity and 7G8 cross-reactivity pattern of C-CSP specific mAbs

Appendix Table S4: Live sporozoites binding and hepatocyte traversal inhibition

Appendix Table S5: Statistical analysis for *in vivo* protection of 1961 mAb against mosquito bite challenge infection (Fig 4F).

Appendix Table S6: X-ray crystallography data collection and refinement data

APPENDIX TABLES

Appendix Table S1: Amino acid (aa) sequences of PfCSP antigens used in ELISAs

Antigen/Peptide	Sequence (aa)
Biotinylated FL-CSP (NF54)	MGILPSPGMPALLSLVSLLSVLLMGCVASLGENDDGNNEDN<br>EKLRKPKHKKLKQPADGNPDPNANPNVDPNANPNVDPNAN<br>PNVDPNANPNANPNANPNANPNANPNANPNANPNANPNA<br>NPNANPNANPNANPNANPNANPNANPNANPNANPNVDPN<br>ANPNANPNKNNQGNGQGHNMPNDPNRNVDENANANSAV<br>KNNNNEEPSDKHIKEYLNKIQNSLSTEWSPCSVTCGNGIQV<br>RIKPGSANKPKDELDYANDIEKKICKMEKCSSVFNVV**NSSG**<br>**SGLNDIFEAQKIEWHEGSHHHHHH**
7G8 FL-CSP	MGILPSPGMPALLSLVSLLSVLLMGCVASLGENDDGNNNN<br>GDNGREGKDEDKRDGNNEDNEKLRKPKHKKLKQPGDGNP<br>DPNANPNVDPNANPNVDPNANPNANPNANPNANPNANPN<br>ANPNANPNANPNANPNANPNANPNANPNANPNANPNANP<br>NVDPNANPNANPNANPNANPNKNNQGNGQGHNMPNDPN<br>RNVDENANANNAVKNNNNEEPSDKHIEQYLKKIKNSISTEW<br>SPCSVTCGNGIQVRIKPGSANKPKDELDYENDIEKKICKMEK<br>C**SSVFNVVNSSGSHHHHHH**
FL-CSP (NF54)	MEYQCYGSSSNTRVLNELNYDNAGTNLYNELEMNYYGKQE<br>NWYSLKKNSRSLGENDDGNNEDNEKLRKPKHKKLKQPAD<br>GNPDPNANPNVDPNANPNVDPNANPNVDPNANPNANPNA<br>NPNANPNANPNANPNANPNANPNANPNANPNANPNANPN<br>ANPNANPNANPNANPNANPNVDPNANPNANPNANPNANP<br>NANPNANPNANPNANPNANPNANPNANPNANPNANPNAN<br>PNANPNANPNANPNANPNKNNQGNGQGHNMPNDPNRNV<br>DENANANSAVKNNNNEEPSDKHIKEYLNKIQNSLSTEWSPC<br>SVTCGNGIQVRIKPGSANKPKDELDYANDIEKKICKMEKCSS<br>VFNVVNSSKLAAALEHHHHHH
N-Junc	GKQPADGNPDPNANPNVDPNANP
$NANP_{10}$	NANPNANPNANPNANPNANPNANPNANPNANPNANPNANP
C-CSP	NKNNQGNGQGHNMPNDPNRNVDENANANSAVKNNNNEE<br>PSDKHIKEYLNKIQNSLSTEWSPCSVTCGNGIQVRIKPGSAN<br>KPKDELDYANDIEKKICKMEKCSVFNVVNSS
C-linker	NKNNQGNGQGHNMPNDPNRNVDENANANSAVKNNNNEE
α-TSR	MGILPSPGMPALLSLVSLLSVLLMGCVAETGYVEFEPSDKHI<br>KEYLNKIQNSLSTEWSPCSVTCGNGIQVRIKPGSANKPKDE<br>LDYANDIEKKICKMEKCPHHHHHHALE
P1	NKNNQGNGQGHNMP
P2	QGHNMPNDPNRNVD
P3	PNRNVDENANANSA
P4	ANANSAVKNNNNEE
P5	NNNNEEPSDKHIKE
P6	DKHIKEYLNKIQNS
P7	NKIQNSLSTEWSPC
P8	TEWSPCSVTCGNGI
P9	TCGNGIQVRIKPGS

P10	RIKPGSANKPKDEL
P11	KPKDELDYANDIEK
P12	ANDIEKKICKMEKC
P13	CKMEKCSSVFNVVN
P14	MEKCSSVFNVVNSS

Appendix Table S2: Sequenced and cloned antibodies per time-point

	**PfCSP Memory B cells**				**Plasmablasts**	
	**Sequenced antibodies**		**Cloned antibodies**		**Sequenced antibodies**	
**Donor**	**III+14**	**III+35**	**III+14**	**III+35**	**III+14**	**III+35**
M1	67	107	24	23	67	NA
M2	25	35	4	13	53	NA
M3	41	36	13	12	216	196
M4	213	134	26	26	129	136
M5	43	NA	10	-	144	NA
M6	NA	37	-	12	NA	NA
M7	29	14	11	5	177	119
M8	NA	34	-	9	NA	86
M9	22	10	5	1	342	184
M10	24	73	9	15	140	NA
M11	NA	46	-	8	NA	NA
M12	132	50	29	12	147	244

Appendix Table S3: Epitope specificity and 7G8 cross-reactivity pattern of C-CSP specific mAbs

mAbs	Affinity	NF54 C-CSP binding (ELISA AUC) after blocking with the indicated mAbs		7G8 FL-CSP binding
**ID**	K_D (M)	**mAb 1710**	**mAb 1512**	**ELISA AUC**
3393	5.11E-10	15.48		1
1805	1.41E-09	21.54		1.3
4192	2.77E-09	18.35		0.8
3667	3.49E-09	22.46		1.4
3666	5.50E-09	50.69		2.9
0566	5.70E-09	18.1		0.6
2801	5.74E-09	42.07		0.6
4595	6.40E-09	19.76		10.1
1593	8.00E-09	19.72		0.5
2889	9.28E-09	18.09		0.6
4121	9.81E-09	36.09		12.7
0621	1.02E-08	40.09		1.6
3743	1.04E-08	22.59		0.7
3033	1.09E-08	17.49		13.4
3133	1.21E-08	18.4		0.6
1891	1.25E-08	48.59		0.9
3451	1.26E-08	69.77		0.8
0937	1.34E-08	28.11		1.4
1984	1.50E-08	26.99		1.3
3116	1.74E-08	23.48		0.9
4025	2.31E-08	145.8	28.33	13.2
4164	2.54E-08	25.04		0.5
3268	2.66E-08	41.77		1.8
4118	2.74E-08	149.3	45.37	10.8
2446	2.78E-08	46.61		1.5
2384	2.80E-08	45.21		0.8
3128	2.91E-08	26.48		2.4
4135	3.15E-08	47.63		2.2
2959	3.51E-08	143.9	75.75	13.1
0762	3.70E-08	41.61		0.8
2421	4.10E-08	69.39		1.4
0709	4.11E-08	144.4	27.04	13.1
3616	4.63E-08	49.29		0.6
2599	6.61E-08	31.44		1.1
0335	7.34E-08	51.57		0.5
3694	9.10E-08	155.2	68.04	13.5
3713	9.58E-08	55.03		0.6
3217	9.95E-08	42.57		12

mAb 1710 (Scally *et al.*, 2017)	7.01E-08	53.74	183.6	0.2
mAb 1512 (Beutler *et al.*, 2022)	2.85E-10	156.8	21.53	ND

mAbs- monoclonal antibodies; ID- identification number; AUC- area under the curve; ND- not determined

Appendix Table S4: Live sporozoites binding and hepatocyte traversal inhibition

		mAbs		Binding affinity	Sporozoite binding (% average)		Traversal inhibition (% average)
**ID**	**VH**	**VL**	**Epitope specificity**	**K_D (M)**	**100 µg/ml**	**1 µg/ml**	**100 µg/ml**
3393	3-21	Vλ3-1	Th2R/Th3R	5.11E-10	0.8		4.1
0566	3-21	Vλ3-21	Th2R/Th3R	5.70E-09	0.4		-3.2
4121	3-30	Vλ1-36	Th2R/Th3R	9.81E-09	0.7		0.7
3743	3-9	Vλ2-23	Th2R/Th3R	1.04E-08	0.6		9.9
4135	3-21	Vλ6-57	Th2R/Th3R	3.15E-08	0.6		-8.3
4025	4-61	Vκ3-20	RII+	2.31E-08	1		3.5
4164	3-7	Vλ3-1	Th2R/Th3R	2.54E-08	0.7		-1.6
4118	3-23	Vλ7-43	RII+	2.74E-08	0.5		5.1
0709	3-48	Vκ3-11	RII+	4.11E-08	0.6		-12.8
2152	3-23	Vκ1-13	ND	1.64E-07	1		2
3764	3-33	Vκ2-30	C-linker	1.72E-07	77	0.9	-12.8
1061	4-59	Vλ1-47	Linear peptide	1.93E-07	0.9		-10.7
0590	1-3	Vλ3-21	ND	2.45E-07	0.6		-13.7
3750	1-3	Vκ1-33	ND	4.17E-07	0.8		16.3
2449	3-9	Vκ1-12	ND	5.04E-07	0.7		3.1

mAbs- monoclonal antibodies; ID- identification number; VH- Variable Ig heavy chain segment; VL- Variable Ig light chain segment; ND- not determined

Appendix Table S5: Statistical analysis for *in vivo* protection of 1961 mAb against mosquito bite challenge infection (Fig 4F).

	**1961 (n=8)**	**317 (n=10)**
**1710 (n=9)**	0.0592	<0.0001
**1961 (n=8)**	-	0.027

P-values of pair comparisons are indicated, Mantel‐Cox log‐rank test, N=2.

Appendix Table S6: X-ray crystallography data collection and refinement data

	**3764 Fab - PfCSP$_{281-294}$**
Beamline	APS-23-ID-B
Wavelength (Å)	1.033167
Space group	$P2_1$
Cell dimensions	
a, b, c (Å)	92.2, 66.7, 95.5
α, β, γ (°)	90, 113.4, 90
Resolution (Å)[a]	40.0-2.36 (2.46-2.36)
No. molecules in ASU	2
No. observations	303,283 (34,515)
No. unique observations	43,719 (5067)
Multiplicity	6.9 (6.7)
R_{merge} (%)[b]	12.3 (63.4)
R_{pim} (%)[c]	5.0 (26.1)
$\langle I/\sigma I \rangle$	10.9 (1.8)
$CC_{1/2}$	99.7 (74.9)
Completeness (%)	98.9 (98.6)
Refinement statistics	
Reflections used in refinement	43,695
Reflections used for R_{free}	1995
Non–hydrogen atoms	7007
Macromolecules	6718
Solvent	289
R_{work}[d]/R_{free}[e]	17.5 / 23.7
Rms deviations from ideality	
Bond lengths (Å)	0.007
Bond angle (°)	0.93
Ramachandran plot	
Favored regions (%)	97.6
Allowed regions (%)	2.3
Wilson B-value (Å^2)	43.7
B-factors (Å^2)	
Average	44.5
Macromolecules	44.5
Solvent	44.3

[a]Values in parentheses refer to the highest resolution bin.
[b]$R_{merge} = \Sigma hkl\, \Sigma i\, |\, Ihkl, i - \langle Ihkl \rangle \,|\, / \Sigma hkl \langle Ihkl \rangle$.
[c]$R_{pim} = \Sigma hkl\, [1/(N-1)]1/2\, \Sigma i\, |\, Ihkl, i - \langle Ihkl \rangle \,|\, / \Sigma hkl \langle Ihkl \rangle$.
[d]$R_{work} = (\Sigma\, |\, |Fo| - |Fc|\, |) / (\Sigma\, |\, |Fo|)$ for all data except as indicated in footnote e.
[e]5% of data was used for the R_{free} calculation.
