# Supplementary material for: Molecular and functional properties of human Plasmodium falciparum CSP C‐terminus antibodies
Source: EMBO Mol Med. 2023 Apr 21;15(6):e17454. doi: 10.15252/emmm.202317454 (PMC10245032; doi:10.15252/emmm.202317454)
Supplement: Supplementary file 2 — Expanded View Figures PDF [file EMMM-15-e17454-s008.pdf]

Expanded View Figures

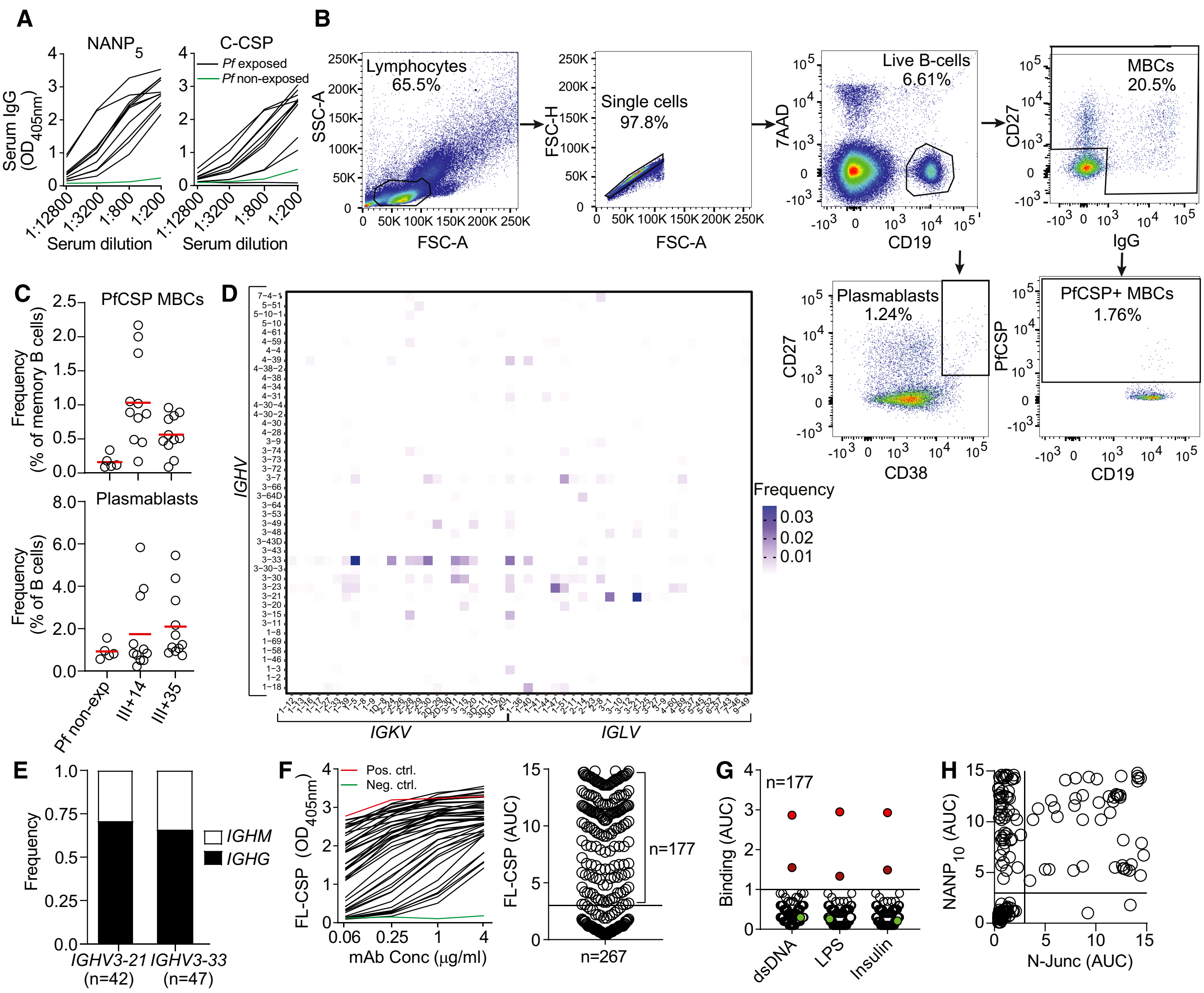

Figure EV1. Immunization with radiation-attenuated *Plasmodium falciparum* sporozoites (PfSPZ Vac) induces strong antibody responses against the PfCSP C terminus.

- A Representative ELISA curves of serum IgG reactivity against PfCSP-derived $NANP_5$ peptide and C-CSP domain.
- B Flow-cytometric single-cell isolation strategy for PfCSP-reactive memory B cells and plasmablasts from a representative immunized donor.
- C Frequency of PfCSP-reactive memory B cells (upper panel) and plasmablasts (lower panel) in PBMCs of immunized donors at the indicated time points (III + 14 and III + 35) and non-immunized donors (Pf non-exp).
- D Paired Ig heavy and light chain V gene usage in PfCSP-reactive memory B cells (n = 1,172).
- E Isotype distribution of *IGHV3-21*- and *IGHV3-33*-encoded mAbs.
- F Representative ELISA curves of mAbs from PfCSP-reactive memory B cells (black) or positive (red) and negative (green) control mAbs 2A10 (Triller *et al*, 2017) and mGO53 (Wardemann *et al*, 2003), respectively, binding to FL-CSP (left) with corresponding AUC values (right; n = 267).
- G ELISA binding strength of FL-CSP-reactive mAbs to LPS, dsDNA or insulin compared to the highly polyreactive mAb ED38 (bright red; Meffre *et al*, 2004), weakly polyreactive mAb JB40 (dark red; Meffre *et al*, 2004), and the non-polyreactive mAb mGO53 (green; Wardemann *et al*, 2003).
- H ELISA binding strength of FL-CSP-reactive mAbs to N-Junc peptide vs $NANP_{10}$.

Data information: Data in (C) represent one biological measurement obtained prior to cell sorting. Data in (F and H) indicate means from three independent technical replicates. Data in (G) are representative of two independent technical replicates.

 © 2023 The Authors

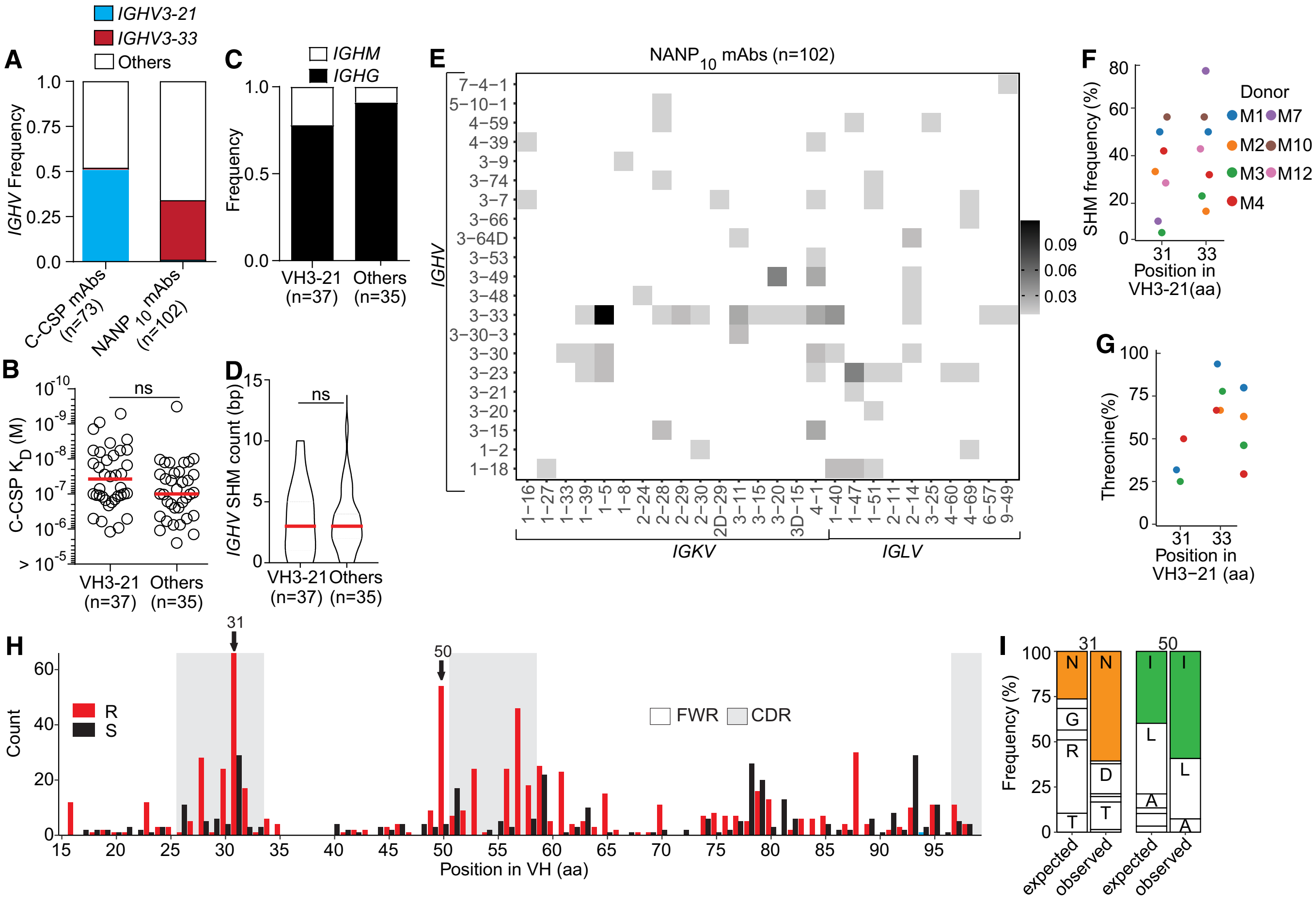

Figure EV2. Molecular characteristics and affinity of C-CSP specific mAbs.

A *IGHV3-21* or *IGHV3-33* gene frequency in C-CSP- and NANP$_{10}$-reactive mAbs.

B–D Comparison of C-CSP specific mAbs encoded by *IGHV3-21* and other *IGHV* genes. Anti-C-CSP SPR affinity (B), isotype distribution (C) and *IGHV* SHM count (D).

E Frequency of NANP$_{10}$-reactive mAbs encoded by the indicated *IGHV* + *IGKV* and *IGHV* + *IGLV* combinations ($n = 102$).

F, G Percentage of VH aa replacement SHM (F) and frequency of Thr at position H.31 and H.33 (G) in VH3-21 mAbs at donor level.

H Amino acid (aa) VH replacement (red bars) and silent (black bars) SHM in VH3-33 mAbs ($n = 256$). FWR, framework region; CDR, complementarity-determining region.

I Observed (Obs) aa usage frequency at position H.31 and H.50 in VH3-33 mAbs carrying a replacement mutation at these positions compared to the expected (Exp) neutral mutation model (Yaari *et al*, 2013; Gupta *et al*, 2015). Single-letters indicate aa residues: A, Ala; D, Asp; G, Gly; I, Ile; L, Leu; N, Asn; R, Arg; T, Thr.

Data information: Data in (F and G) represent donors with at least 5 VH3-21 mAbs and ≥ 4 mAbs with mutations at position H.33, respectively. Red lines in (B) and (D) indicate geometric and arithmetic mean values, respectively. ns, non-significant, two-tailed Mann–Whitney test (B, D). Data in (B) are representative of two independent technical replicates.

© 2023 The Authors

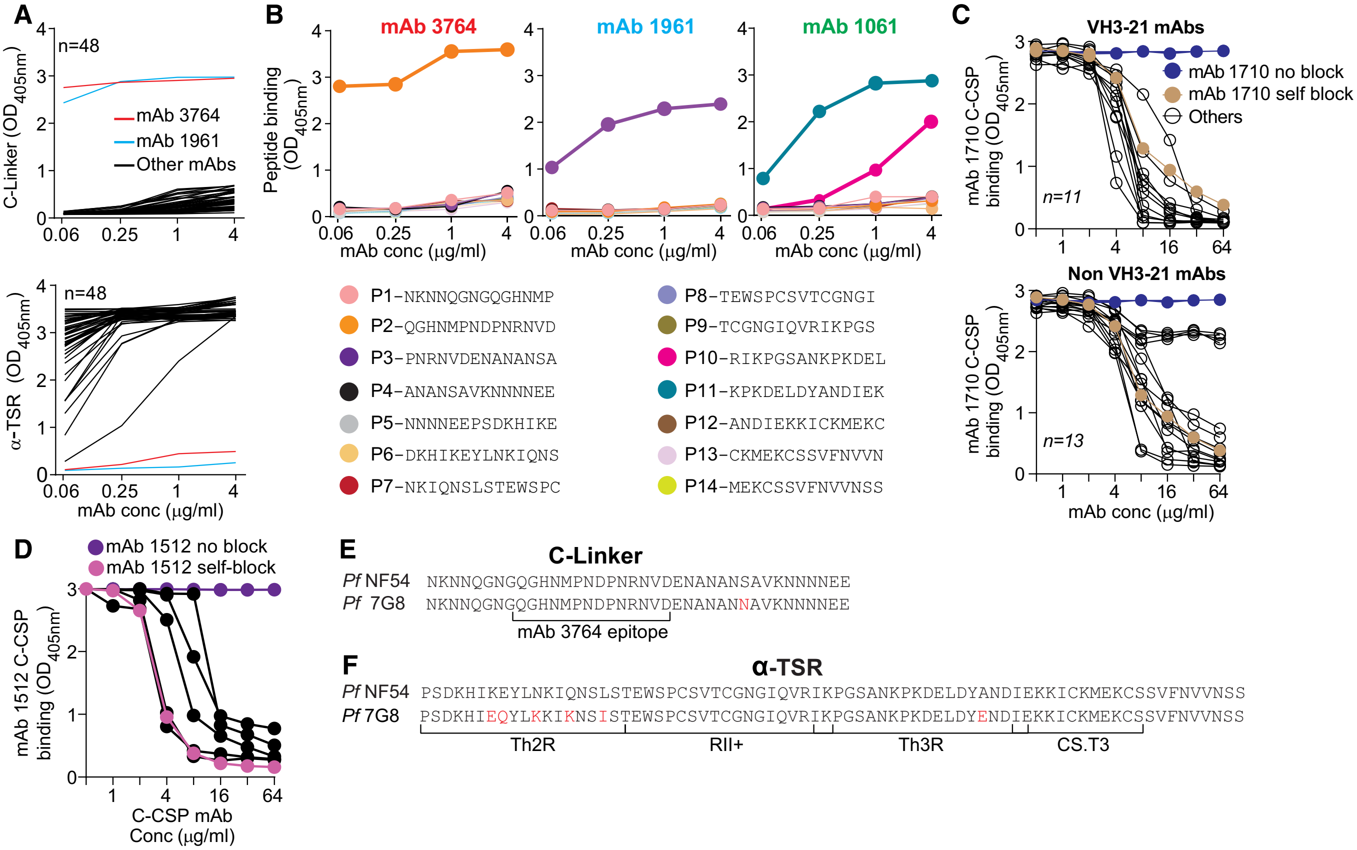

Figure EV3. C-CSP specific mAbs target preferentially two distinct conformational epitopes in the α-TSR domain.

A Representative ELISA binding curves for all C-CSP specific mAbs (black lines) to the C-linker (upper panel) and α-TSR (lower panel). The C-linker specific mAb 3764 (red line) and C-linker cross-reactive mAb 1961 (light blue line) are highlighted.

B Representative ELISA binding curves to overlapping peptides (P1-P14) covering the complete C-CSP for mAb 3764 (left), mAb 1961 (middle) and mAb 1061 (right). Amino acid sequences of the peptides are indicated.

C Representative ELISA curves illustrating the ability of individual VH3-21 (upper panel) and non-VH3-21 (lower panel) C-CSP specific mAbs (black lines) to block binding of mAb 1710 (Scally *et al*, 2018) to C-CSP. mAb 1710 C-CSP binding without blocking (blue) and after self-blocking (golden) is shown for comparison.

D ELISA curves showing C-CSP reactivity of mAb 1512 (Beutler *et al*, 2022) in a blocking ELISA with mAbs that do not block C-CSP binding of mAb 1710 (black). mAb 1512 binding without blocking (purple) and after self-blocking (magenta) is shown for comparison.

E, F Alignment of the PfCSP NF54 and 7G8 C-linker (E) and α-TSR (F) aa sequences. Amino acids that differ are highlighted in red.

 © 2023 The Authors

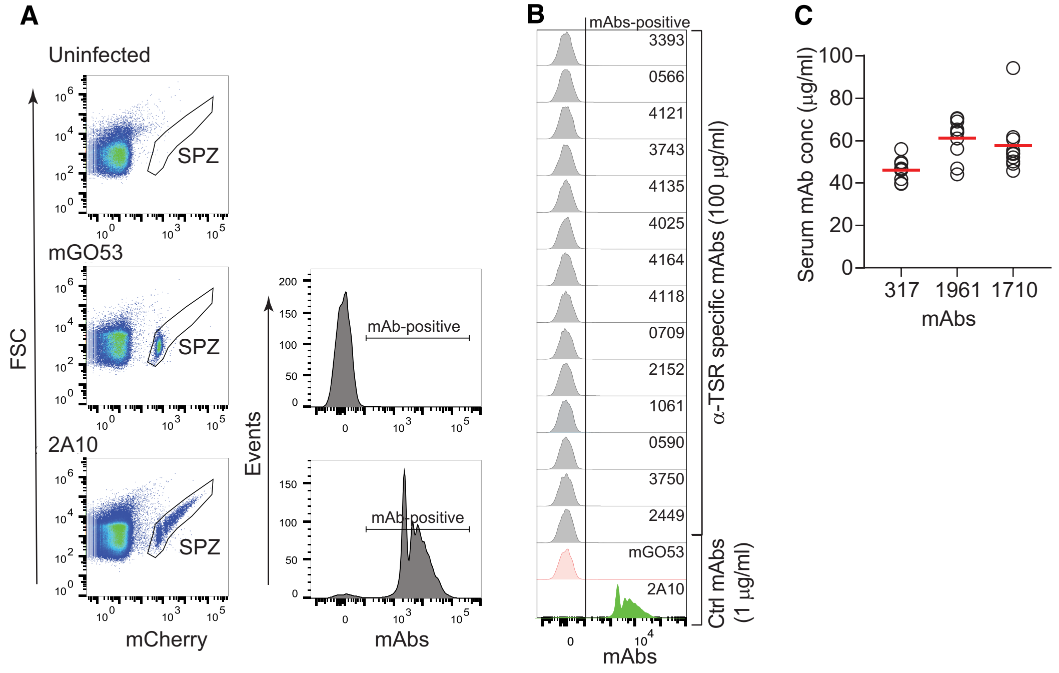

Figure EV4. Gating strategy to assess mAb binding to live PbPfCSP(mCherry) sporozoites.

A Flow cytometric gating strategy to analyze the proportion of mAb-positive PbPfCSP(mCherry) live sporozoites. Live mCherry-positive sporozoites (SPZ) were gated. Salivary gland extracts from uninfected mosquitoes were used as sporozoite gating control. The sporozoite binding profile of the negative control mAb mGO53 (Wardemann *et al*, 2003) and the positive control anti-NANP mAb 2A10 (Triller *et al*, 2017) are shown for comparison.

B Representative flow-cytometric profiles of live PbPfCSP(mCherry) sporozoites recognized by α-TSR-specific mAbs (100 μg/ml) as determined by flow cytometry. The positive control anti-NANP mAb 2A10 (1 μg/ml; Triller *et al*, 2017) and negative control non-PfCSP reactive mAb mGO53 (1 μg/ml; Wardemann *et al*, 2003) are shown for comparison.

C mAb serum concentrations in individual mice (n = 10 for mAb 317, n = 8 for mAb 1961, and n = 9 for mAb 1710) after passive transfer of the indicated monoclonal antibodies. The data in C only included mice that had detectable serum IgG concentrations of the indicated mAbs at the time of the challenge.

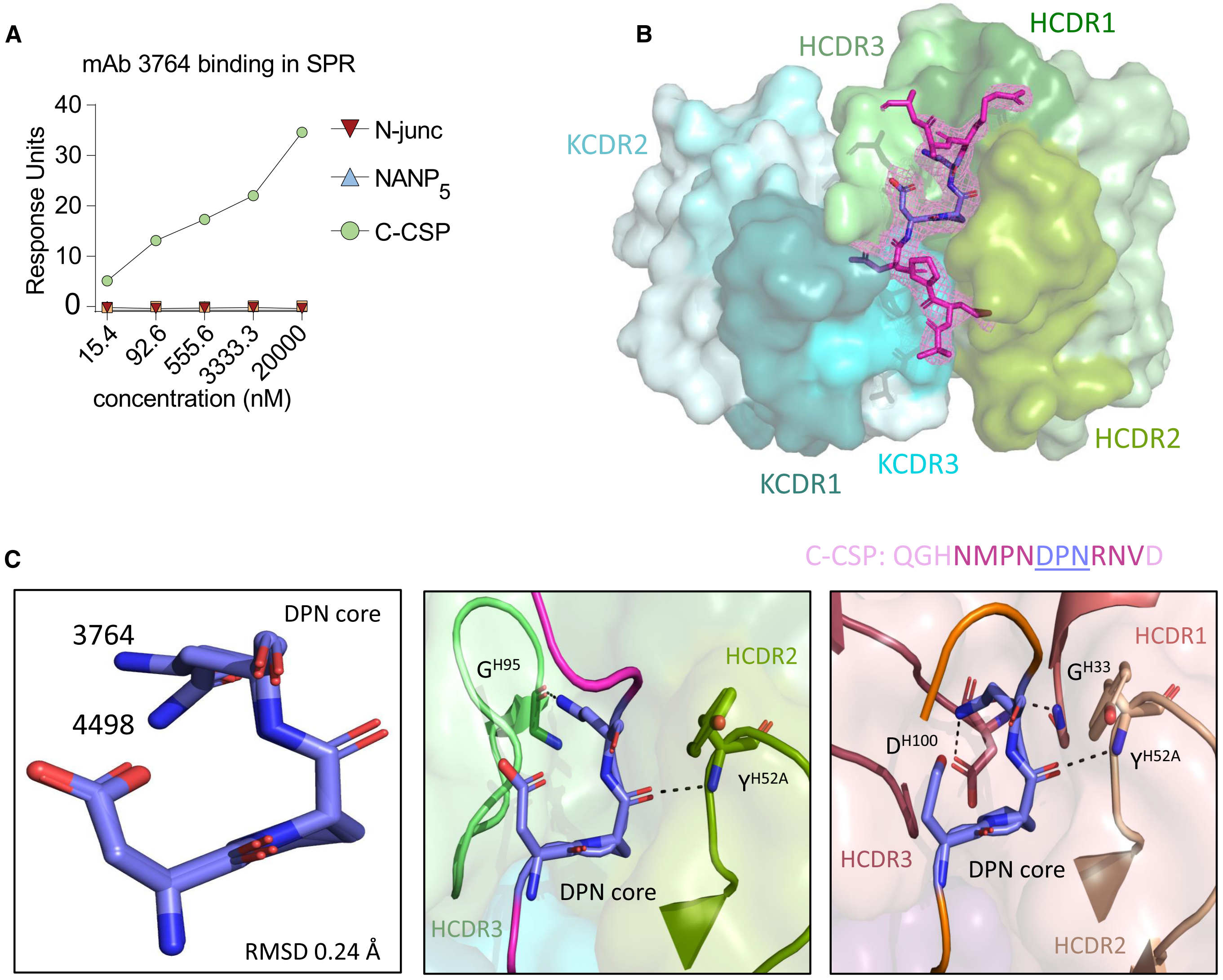

Figure EV5. Specificity of mAb 3764 towards the PfCSP C-terminal region.

A mAb 3764 SPR affinity measurement against peptides covering the N-term junction (N-Junc), $NANP_5$ or C-CSP at various concentrations.

B Composite omit map electron density contoured at 1 σ (light magenta mesh) around the C-CSP peptide (magenta).

C (Left) Superposition of the DPN core (deep blue) in crystal structures of mAb 3764 bound to the indicated C-CSP peptide (magenta) and mAb 4498 (Murugan *et al*, 2020) bound to the NDN3 peptide (orange) (PDB ID: 6ULF). (Middle) mAb 3764 HCDR2 and 3 (pea and light green, respectively) are shown interacting with the C-CSP peptide. (Right) mAb 4498 HCDR1, 2, and 3 (dark salmon, raspberry and wheat, respectively) are shown interacting with NDN3.

Data information: Data in (A) is a representative of two independent technical replicates.

 © 2023 The Authors
