# Supplementary material for: Molecular and functional properties of human Plasmodium falciparum CSP C‐terminus antibodies
Source: EMBO Mol Med. 2023 Apr 21;15(6):e17454. doi: 10.15252/emmm.202317454 (PMC10245032; doi:10.15252/emmm.202317454)
Supplement: Supplementary file 4 — PDF+ [file EMMM-15-e17454-s001.pdf]

Article

SOURCE DATA | TRANSPARENT PROCESS | OPEN ACCESS

EMBO Molecular Medicine

Molecular and functional properties of human *Plasmodium falciparum* CSP C-terminus antibodies

Opeyemi Ernest Oludada[1,2], Giulia Costa[3], Clare Burn Aschner[4], Anna S Obraztsova[1,2], Katherine Prieto[4], Caterina Canetta[1], Stephen L Hoffman[5], Peter G Kremsner[6,7], Benjamin Mordmüller[6,8], Rajagopal Murugan[1,†], Jean-Philippe Julien[4,9,*,†], Elena A Levashina[3,**,†] & Hedda Wardemann[1,***,†]

Abstract

Human monoclonal antibodies (mAbs) against the central repeat and junction domain of *Plasmodium falciparum* circumsporozoite protein (PfCSP) have been studied extensively to guide malaria vaccine design compared to antibodies against the PfCSP C terminus. Here, we describe the molecular characteristics and protective potential of 73 germline and mutated human mAbs against the highly immunogenic PfCSP C-terminal domain. Two mAbs recognized linear epitopes in the C-terminal linker with sequence similarity to repeat and junction motifs, whereas all others targeted conformational epitopes in the α-thrombospondin repeat (α-TSR) domain. Specificity for the polymorphic Th2R/Th3R but not the conserved RII+/CS.T3 region in the α-TSR was associated with *IGHV3-21*/*IGVL3-21* or *IGLV3-1* gene usage. Although the C terminus specific mAbs showed signs of more efficient affinity maturation and class-switching compared to anti-repeat mAbs, live sporozoite binding and inhibitory activity was limited to a single C-linker reactive mAb with cross-reactivity to the central repeat and junction. The data provide novel insights in the human anti-C-linker and anti-α-TSR antibody response that support exclusion of the PfCSP C terminus from malaria vaccine designs.

Keywords Affinity maturation; Antibodies; Malaria; PfCSP; Plasmodium
Subject Categories Immunology; Microbiology, Virology & Host Pathogen Interaction; Structural Biology
DOI 10.15252/emmm.202317454 | Received 19 January 2023 | Revised 11 March 2023 | Accepted 23 March 2023 | Published online 21 April 2023
EMBO Mol Med (2023) 15: e17454
See also: **J Murdoch & J Baum** (June 2023)

Introduction

Plasmodium falciparum (Pf) malaria is a mosquito-borne parasitic disease that is highly endemic in sub-Saharan Africa, where it remains the primary cause of childhood illness and death (WHO, 2021). RTS,S/AS01 (Mosquirix™), the only marketed malaria vaccine, has been recommended for widespread use among children in areas with high to moderate Pf transmission.

RTS,S/AS01 is a subunit vaccine that targets Pf circumsporozoite protein (PfCSP), the major protein on the surface of sporozoites that are transmitted to humans during the blood meal of infected *Anopheles* mosquitoes (Swearingen *et al*, 2016). PfCSP consists of three domains: a poorly characterized N-terminal domain (N-CSP), an unstructured central repeat region with large numbers of repeating four amino-acid motifs, and a C-terminal domain (C-CSP) with a linker (C-linker) and α-thrombospondin type-1 repeat (α-TSR) subdomain that is attached to the cell membrane by a GPI anchor (Dame *et al*, 1984; McCutchan *et al*, 1996; Wang *et al*, 2005; Doud *et al*, 2012). RTS,S/AS01 contains a genetic fusion protein of an N-terminally truncated form of PfCSP from the Pf clone 3D7, derived from the laboratory strain NF54, and hepatitis B surface antigen (HbsAg), which is complexed with free HbsAg to form virus-like particles and boost immunogenicity (Gordon *et al*, 1995; Stoute *et al*, 1997; Bojang *et al*, 2001; Casares *et al*, 2010; Agnandji *et al*, 2011). The PfCSP part of RTS,S comprises 18.5 of the 38 repeating asparagine (N)—alanine (A)—asparagine (N)—proline (P) motifs in the central repeat domain (Zavala *et al*, 1985; Stoute *et al*, 1997) and the complete C-CSP. The characteristic NANP motifs are fully conserved in Pf parasites (with only slight variations in the number of repeating units) independent of their geographic origin

1 B Cell Immunology, German Cancer Research Center, Heidelberg, Germany
2 Biosciences Faculty, University of Heidelberg, Germany
3 Vector Biology Unit, Max Planck Institute for Infection Biology, Berlin, Germany
4 The Hospital for Sick Children Research Institute, Toronto, ON, Canada
5 Sanaria Inc., Rockville, MD, USA
6 Institute of Tropical Medicine, Tübingen, Germany
7 Centre de Recherches de Lambaréné (CERMEL), Lambaréné, Gabon
8 Radboud University Medical Center, Nijmegen, The Netherlands
9 Departments of Biochemistry and Immunology, University of Toronto, Toronto, ON, Canada
*Corresponding author. Tel: +1 416 813 7654; E-mail: jean-philippe.julien@sickkids.ca
**Corresponding author. Tel: +49 30 28460223; E-mail: levashina@mpiib-berlin.mpg.de
***Corresponding author. Tel: +49 6221 421270; E-mail: h.wardemann@dkfz.de
†These authors contributed equally to this work

© 2023 The Authors. Published under the terms of the CC BY 4.0 license

and are long known to be the target epitopes of protective antibodies (Nardin *et al*, 1982; Zavala *et al*, 1983).

The successful clinical development of RTS,S/AS01 in contrast to the other numerous malaria vaccine candidates confirms the potency of PfCSP as a promising vaccine target. However, the overall efficacy is limited and protection is short-lived (Agnandji *et al*, 2011; Asante *et al*, 2011; Olotu *et al*, 2011, 2016). To guide the design of a more efficacious PfCSP-based malaria vaccine, recent studies have characterized large numbers of human monoclonal antibodies (mAbs) against PfCSP (Oyen *et al*, 2017; Triller *et al*, 2017; Kisalu *et al*, 2018; Murugan *et al*, 2018; Tan *et al*, 2018; Wang *et al*, 2020). Their molecular and functional characterization provided deep insights in the development and potency of anti-repeat antibodies, and identified new epitopes of protective mAbs in the N-terminal junction, with alternating NANP and NANP-like (NPDP, NVDP) motifs that are not included in RTS,S.

In contrast to the large numbers of well-characterized antibodies against the central repeat domain and N-terminal junction, the role of antibodies against N-CSP and C-CSP remains controversial. N-CSP is overall poorly immunogenic and undergoes proteolytic cleavage at region I (RI), a stretch of six amino acids that is highly conserved in all *Plasmodium* species (Coppi *et al*, 2005; Espinosa *et al*, 2015). Human monoclonal antibodies against N-CSP distal to RI have not been described, and the few known mouse monoclonal antibodies show little sporozoite reactivity and parasite inhibitory activity (Herrera *et al*, 2015; Thai *et al*, 2020).

Although immunization with recombinant PfCSP induces dominant humoral responses against C-CSP in animal models and humans (Genito *et al*, 2017; Cawlfield *et al*, 2019; Hutter *et al*, 2022), the limited number of human C-CSP mAbs that have been described so far did not allow deep analyses of their gene features, epitope breadth or preferences, and parasite-inhibitory activity. Two target sites of human monoclonal antibodies have been identified in the α-TSR subdomain of C-CSP: the highly polymorphic Th2R/Th3R region and the conserved RII+/CS.T3 region (Scally *et al*, 2018; Beutler *et al*, 2022). However, whether binding to these epitopes is associated with specific Ig genes, whether other epitopes are targeted by anti-C-CSP antibodies, and whether epitope specificity is linked to differences in parasite inhibition remains unclear. To address these questions, we characterized the molecular and functional properties, as well as binding-specificity of a large panel of recombinant human monoclonal antibodies cloned from memory B cells of malaria-naïve individuals immunized with aseptic, cryopreserved, radiation-attenuated Pf sporozoites (PfSPZ Vaccine; Mordmuller *et al*, 2022).

Results

Immunization with radiation-attenuated *Plasmodium falciparum* sporozoites (PfSPZ) induces antibody responses against C-CSP

To identify individuals with antibody responses against C-CSP, we measured the humoral response in sera from 12 malaria-naive European volunteers 28 days after three direct venous inoculation (DVI) immunizations with 900,000 aseptic, purified, radiation-attenuated Pf NF54 sporozoites (Sanaria® PfSPZ Vaccine; Mordmuller *et al*, 2022). Comparison of the IgG response against a peptide representing the central NANP repeat ($NANP_5$) and against C-CSP showed responses to both domains (Figs 1A and EV1A; Appendix Table S1).

To directly compare the anti-C-CSP and anti-repeat antibody response at monoclonal level, we isolated single circulating PfCSP-reactive memory B cells from all donors at 14 and 35 days after the third immunization (III + 14, III + 35; Fig EV1B and C) and amplified the paired immunoglobulin (Ig) heavy and light chain genes. Sequencing of the RT-PCR Ig gene products showed that in all donors at both time points, the anti-PfCSP response was dominated by memory B cells expressing VH3-21 and VH3-33 Igs, frequently in association with Vλ3-21 or Vλ3-1 and Vκ1-5 light chains, respectively (Figs 1B and EV1D). Both *IGHV* genes were enriched in the PfCSP-reactive memory B cell pool compared to circulating plasmablasts from the same individuals and time points that represent the B cell response to all sporozoite antigens (Fig 1C). The high abundance of VH3-33 expressing PfCSP-reactive memory B cells and frequent association with Vκ1-5 suggested that these cells recognized the central repeat, similar to VH3-33 antibodies induced by immunization with non-irradiated sporozoites (Murugan *et al*, 2018). Compared to VH3-33 antibodies, which were mostly IgM carrying a few somatic hypermutations (SHM), the VH3-21 response was dominated by IgG cells with higher mean SHM counts (Fig 1D and E), indicating their participation in germinal center (GC) responses.

To determine how VH3-21 usage was linked to PfCSP-reactivity, we cloned and expressed 267 monoclonal antibodies (mAbs) covering the overall repertoire diversity of PfCSP-reactive memory B cells, including 42 VH3-21 and 47 VH3-33 antibodies with similar isotype distribution (Fig EV1E; Appendix Table S2 and Dataset EV1). From this panel, 177 (66%) recombinant mAbs showed ELISA reactivity with full-length PfCSP (FL-CSP) and lacked binding to non-related antigens, demonstrating their specificity for PfCSP (Fig EV1F and G). Of these FL-CSP specific mAbs, 102 showed reactivity to a peptide representing the central NANP repeat ($NANP_{10}$), and 73 mAbs recognized the C-CSP domain (Fig 1F). Two mAbs lacked binding to $NANP_{10}$ or C-CSP, but bound a peptide covering the junction motifs (N-junc) (Fig EV1H). NANP-reactive mAbs were identified in all donors and C-CSP-reactive mAbs in all but one donor (Fig 1G).

To determine whether any of the C-CSP-reactive antibodies showed cross-reactivity with the repeat domain including the N-terminal junction, as previously reported (Murugan *et al*, 2020), we measured binding of the mAbs to $NANP_{10}$ and the N-junc peptide. With one exception (mAb 1961), all C-CSP-reactive mAbs were highly specific, whereas most of the $NANP_{10}$-binders showed cross-reactivity to the junction as expected (Fig 1H and I). Thus, immunization with radiation-attenuated sporozoites elicited PfCSP C-CSP specific and repeat/junction cross-reactive humoral and memory B cell responses associated with *IGHV3-21* and *IGHV3-33* gene signatures, respectively.

C-CSP specific mAbs are frequently encoded by *IGHV3-21*

To further characterize the panel of 73 C-CSP specific mAbs, we determined their binding strength by surface plasmon resonance (SPR; Fig 2A). The mAbs recognized C-CSP with a wide range of affinities (10^{-5} to 10^{-10} M). Ig gene sequence analysis showed that half (37/73) of all C-CSP binders were encoded by *IGHV3-21* most of

© 2023 The Authors

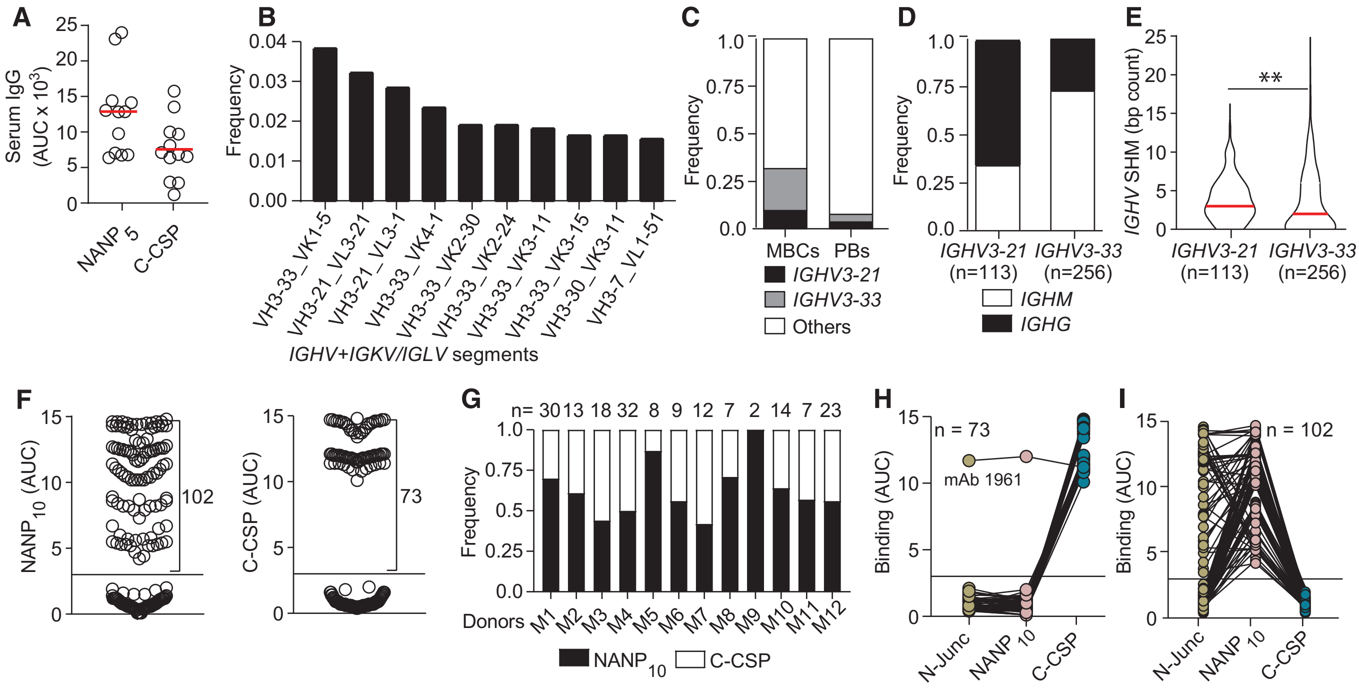

Figure 1. B cell response against the PfCSP C terminus.

A Anti-NANP$_5$ and anti-C-CSP serum IgG. Circles represent ELISA area under the curve (AUC) values for individual donors (n = 12).

B Ten most frequent Ig heavy and light chain V gene pairs in PfCSP-reactive memory B cells (MBCs; n = 1,172).

C Usage frequency of *IGHV3-21*, *IGHV3-33* and other *IGHV* genes in PfCSP-reactive MBCs (n = 1,172) and plasmablasts (PBs; n = 2,380).

D, E Isotype distribution (D) and somatic hypermutation (SHM) count (E) in *IGHV3-21* and *IGHV3-33* genes from MBCs in (B, C).

F NANP$_{10}$ (left) and C-CSP (right) binding of FL-CSP-reactive mAbs (n = 177). Circles represent ELISA AUC values for each mAb.

G Frequency of NANP$_{10}$ and C-CSP reactive mAbs per donor. The number of tested mAbs is indicated.

H, I Cross-reactivity of C-CSP (H) and NANP$_{10}$ (I) reactive mAbs with the PfCSP N-junc, NANP$_{10}$ and C-CSP; n indicates the number of mAbs tested.

Data information: Red lines in (A and E) indicate mean values. Black horizontal lines in (F), (H) and (I) indicate the threshold for binding. (E). The statistical significance in E was assessed by two-tailed Mann–Whitney test: $^{**}P$ = 0.0017. Data in (A) are representative of two independent technical replicates. Data in (F, H and I) are obtained from three independent technical replicates and indicated as mean.

Source data are available online for this figure.

which paired with *IGLV3-21* or *IGLV1-3* genes, demonstrating the strong link between this gene combination and anti-C-CSP antibody reactivity (Figs 2B and EV2A). The other half of the anti-C-CSP antibodies used diverse gene combinations and showed no significant difference in affinity, isotype distribution or somatic mutation load compared to VH3-21 mAbs (Figs 2B and EV2B–D). As expected, VH3-33 heavy chains paired with Vκ1-5 light chains were highly abundant among the NANP-reactive mAbs, similar to VH3-33/Vκ1-5 anti-repeat antibodies induced by immunization with non-irradiated sporozoites (Fig EV2A and E).

Although most C-CSP specific antibodies were class-switched and carried somatic mutations as signs of affinity maturation, we identified several VH3-21 and non-VH3-21 germline antibodies with high C-CSP affinity suggesting that the naïve human B cell repertoire contains numerous C-CSP-reactive precursor cells (Fig 2C). Sequence alignments of all VH3-21 antibodies showed strong signs of selection indicated by enrichment of replacement mutations in CDRs compared to framework regions (FWR; Fig 2D). Almost half of the VH3-21 antibodies carried replacement mutations at two positions in HCDR1 (H.31, H.33) and threonine was strongly enriched in these positions compared to the neutral mutation model (Figs 2D and E, and EV2F and G; Yaari *et al*, 2013; Gupta *et al*, 2015). VH3-21 mAbs carrying the selected H.S33T mutation showed on average higher C-CSP affinities than mAbs with the germline residue H.S33 or with non-selected mutations demonstrating the role of the H.S33T exchange in affinity maturation (Fig 2F). The data shows a strong link of VH3-21 gene segment usage with C-CSP reactivity and provides direct evidence for effective affinity maturation of the anti-C-CSP response similar to the selection of H.S31N and H.V50I mutations in VH3-33 antibodies (Fig EV2H and I), which mediate high affinity to repeating NANP motifs (Imkeller *et al*, 2018).

C-CSP specific antibodies preferentially target two distinct conformational epitopes in the α-TSR domain

To determine which of the two C-terminal subregions (Fig 3A) the C-CSP specific and C-CSP cross-reactive antibodies recognized, we measured binding to the C-linker (aa 273–310) and the α-TSR domain (aa 311–384) by ELISA (Figs 3B and EV3A). With two exceptions that recognized the C-linker region (mAb 3764, and mAb 1961 with NANP$_{10}$ and N-junc cross-reactivity; Fig 1H), all mAbs showed specificity for the α-TSR domain.

© 2023 The Authors

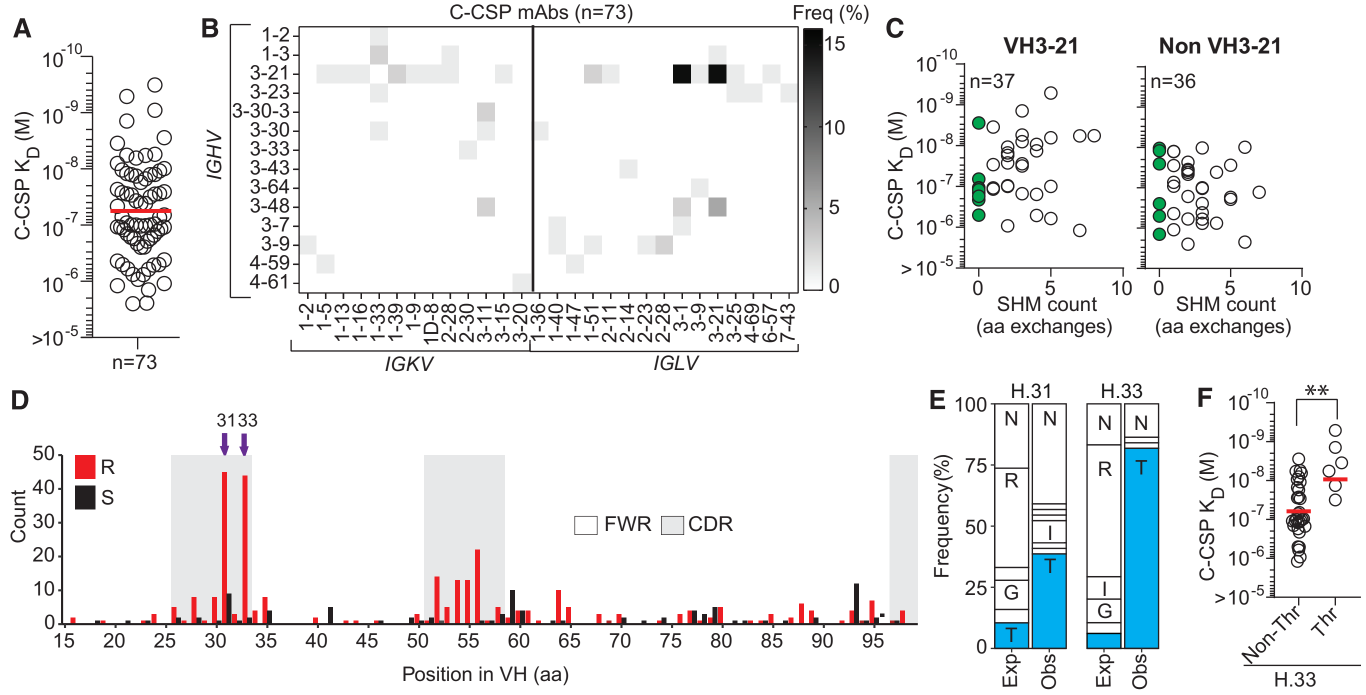

Figure 2. C-CSP specific mAbs are frequently encoded by *IGHV3-21*.

A SPR affinity of C-CSP reactive mAbs.

B Frequency of mAbs encoded by the indicated *IGHV* and *IGKV* or *IGLV* pairs.

C VH SHM load of VH3-21 and non-VH3-21 mAbs. mAbs with unmutated VH are highlighted in green; n indicates the number of mAbs tested.

D Amino acid (aa) VH replacement (red bars) and silent (black bars) SHM in VH3-21 mAbs (n = 113). FWR, framework region; CDR, complementarity-determining region.

E Observed (Obs) aa usage frequency at position H.31 and H.33 in VH3-21 mAbs carrying a replacement mutation at these positions compared to the expected (Exp) neutral mutation model (Yaari *et al*, 2013; Gupta *et al*, 2015). Single-letters indicate aa residues: G, Gly; I, Ile; N, Asn; R, Arg; T, Thr.

F C-CSP affinity for selected VH3-21 mAbs with or without Thr mutation at position H.33.

Data information: The statistical significance in (F) was assessed by two-tailed Mann–Whitney test: **P = 0.0019. Red horizontal lines in (A, F) indicate geometric mean values. Data in (A, C and F) are representative of two independent technical replicates.
Source data are available online for this figure.

To define whether any of the C-CSP-reactive antibodies recognized linear epitopes, we tested their binding to 14 overlapping peptides (P1-14) covering the complete NF54 C-CSP (Figs 3C and EV3B; Appendix Table S1). Only three mAbs showed reactivity in these assays: the C-linker specific mAb 3764 with specificity for P2 spanning aa 281–294 (QGHNMPNDPNRNVD) and the α-TSR-reactive mAb 1061, which bound P10 and P11 covering aa 345–366 in Th3R region. These two peptides overlap by six aa (KPKDEL), suggesting that the mAb 1061 target epitope covers aa KPKDEL. The C-CSP, junction and repeat cross-reactive mAb 1961 showed reactivity with P3 (PNRNVDENANANSA) suggesting that C-CSP binding of this mAb might be mediated via the NANA motif, similar to the previously reported C-CSP, junction and repeat cross-reactive mAb 3246 (Murugan *et al*, 2020). All other C-CSP specific mAbs (70/73) lacked peptide reactivity indicating that they recognized conformational epitopes.

Similar to the previously reported anti-α-TSR mAb 1710 (Scally *et al*, 2018), many of these antibodies were encoded by *IGHV3-21*, suggesting that these antibodies and mAb 1710 might recognize the same or overlapping epitopes in Th2R and Th3R. To address this question, we selected 21 *IGHV3-21*-encoded and 17 non-*IGHV3-21* encoded C-CSP specific mAbs with similar or higher C-CSP affinity compared to mAb 1710 (≤ 10^{-7} M) and tested their ability to block mAb 1710 binding to C-CSP by ELISA (Figs 3D and EV3C; Appendix Table S3). All 21 VH3-21 and most of the non-VH3-21 (12/17) mAbs blocked binding of mAb 1710 demonstrating that the vast majority of anti-C-CSP antibodies shared specificity for the same or overlapping epitopes in the Th2R/Th3R. Only five of the 17 non-VH3-21 mAbs with diverse Ig genes did not interfere with mAb 1710 binding (Fig 3D). To determine their epitope specificity, we tested their ability to block mAb 1512, an RTS,S/AS01-induced C-CSP mAb with specificity for a conformational epitope in the conserved RII+/CS.T3 of the α-TSR subdomain (Beutler *et al*, 2022). All five antibodies blocked mAb 1512 binding, demonstrating their specificity for the conserved RII+/CS.T3 epitope independent of their Ig gene usage (Figs 3E and EV3D).

The Th2R and to lesser extent the C-linker and Th3R are highly polymorphic (Gandhi *et al*, 2012; Aragam *et al*, 2013). To evaluate whether the C-CSP specific antibodies were able to accommodate these polymorphisms, we tested their ability to bind to PfCSP from 7G8, which differs from NF54 C-CSP by seven aa: one aa in the C-linker and six aa in the α-TSR region (five in Th2R and one in

 © 2023 The Authors

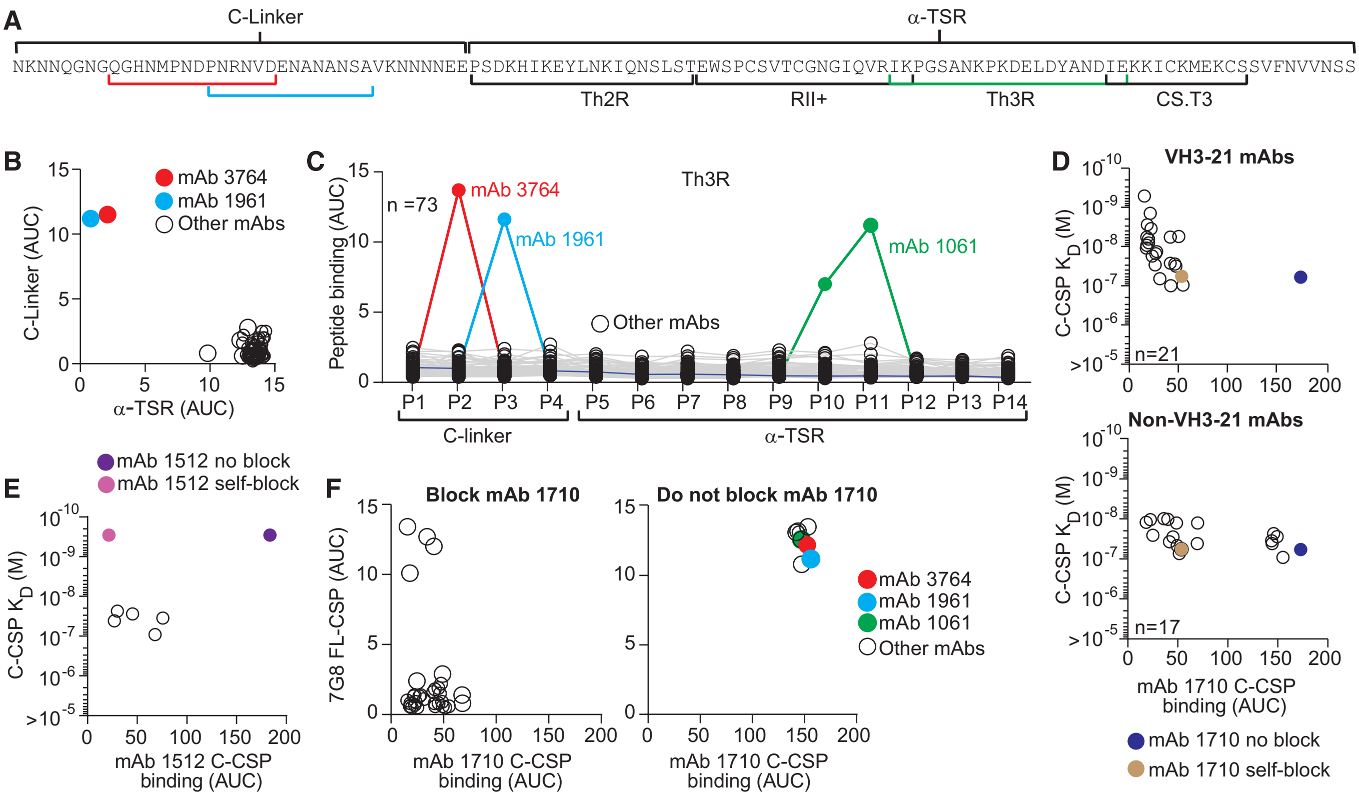

Figure 3. Epitope specificity of C-CSP specific mAbs.

A NF54 PfCSP C-CSP aa sequence. The C-linker and α-TSR domain with Th2R, RII+, Th3R and CS.T3 are indicated.

B, C ELISA reactivity of C-CSP-reactive mAbs (n = 73) with the C-linker and α-TSR domain (B) and overlapping C-CSP peptides (P1-P14; Appendix Table S1 for sequences) (C).

D C-CSP reactivity of the α-TSR-specific mAb 1710 (Scally *et al*, 2018) in a blocking ELISA with C-CSP specific VH3-21 (n = 21; upper panel) or non-VH3-21 (n = 17; lower panel) mAbs with the indicated SPR affinities. mAb 1710 binding without blocking (blue) and after self-blocking (gold) is shown for comparison.

E C-CSP reactivity of mAb 1512 (Beutler *et al*, 2022) in a blocking ELISA with mAbs (with the indicated SPR affinities; open circles) that do not block C-CSP binding of mAb 1710 (from D lower panel). mAb 1512 binding without blocking (purple) and after self-blocking (magenta) is shown for comparison.

F ELISA cross-reactivity with Pf-7G8 FL-CSP of CSP-specific mAbs that block (left) or do not block (right) mAb 1710 binding to NF54 C-CSP (D).

Data information: Highlighted mAbs (B, C and F) with their binding sites (highlighted in A) are mAb 3764 (C-linker specific; red), mAb 1961 (C-linker, repeat and junction cross-reactive; light blue), mAb 1061 (α-TSR peptide 10 and peptide 11 specific; green). Data in (B–F) are representative of two independent technical replicates.
Source data are available online for this figure.

Th3R; Fig EV3E and F). With four exceptions, none of the mAb 1710-blocking mAbs recognized 7G8 PfCSP (Fig 3E). In contrast, mAbs 3764, 1961 and 1061 with linear peptide epitopes in the C-linker and Th3R, respectively, and the five mAbs with reactivity to the conformational mAb 1512 epitope in RII+/CS.T3 showed cross-reactivity with 7G8 (Fig 3F). Thus, only antibodies against the conserved linear or conformational C-CSP epitopes not overlapping with the polymorphic mAb 1710 binding site showed 7G8 cross-reactivity.

In summary, the vast majority of anti-C-CSP antibodies showed the same specificity as mAb 1710, demonstrating the strong immunogenicity of their polymorphic target epitopes in the α-TSR domain. Binding to this site was strongly linked with *IGHV3-21* gene usage since all VH3-21 mAbs bound this epitope. Antibodies against conserved epitopes in the α-TSR domain or the C-linker (conformational or linear) used diverse IgH and IgL combinations and showed cross-reactivity to PfCSP from 7G8, but were overall rare.

C-CSP specific antibodies fail to bind live sporozoites and lack parasite inhibitory activity

Differences in epitope specificity and Ig gene usage of antibodies might be linked to their parasite binding and inhibitory capacities. We selected 15 representative antibodies with high C-CSP affinity against the linear and conformational epitopes in the C-linker and α-TSR domain and measured their ability to bind live fluorescent *P. berghei* sporozoites expressing PfCSP (PbPfCSP (mCherry), Fig EV4A; Appendix Table S4). Compared to the anti-repeat mAb 2A10 (Triller *et al*, 2017), which showed strong binding at low concentration (1 μg/ml) in flow cytometry, none of the mAb 1710-like or mAb 1512-like anti-α-TSR mAbs recognized live sporozoites even at 100 μg/ml regardless of their epitope specificity and affinity (Figs 4A and EV4B), supporting the earlier observations that the α-TSR domain on the surface of live sporozoites was not accessible for antibody binding (Scally *et al*, 2018; Wang *et al*, 2021). Weak

© 2023 The Authors

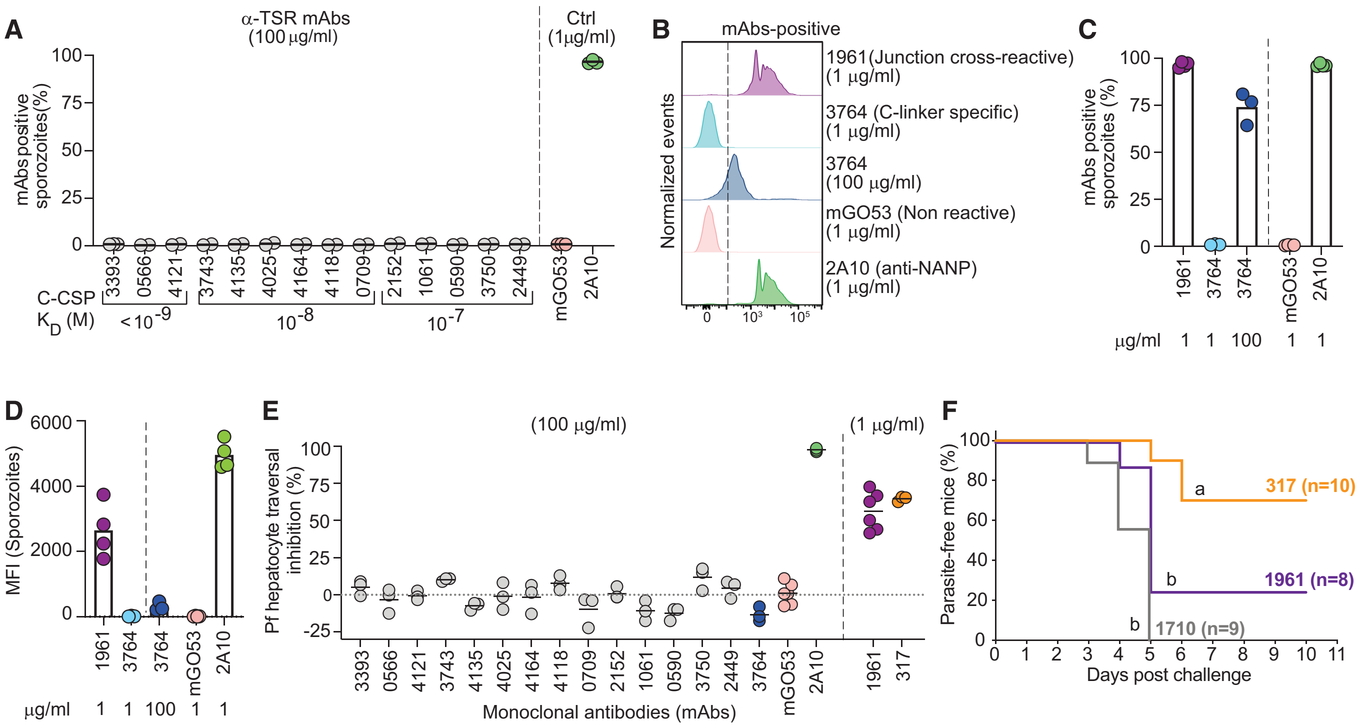

Figure 4. C-CSP specific mAbs lack anti-parasite activity.

A Percent of live PbPfCSP(mCherry) sporozoites recognized by α-TSR-specific mAbs (100 μg/ml) as determined by flow cytometry, with the respective affinities measured by SPR. The positive control anti-NANP mAb 2A10 (1 μg/ml; Triller *et al*, 2017) and negative control non-PfCSP reactive mAb mGO53 (1 μg/ml; Wardemann *et al*, 2003) are shown for comparison (n = 2–3).

B–D Representative flow cytometry profiles (B), percentage of mAb-positive live PbPfCSP(mCherry) sporozoites (C), and mean fluorescence intensity (MFI) (D) for the junction cross-reactive mAb 1961, C-linker-specific mAb 3764 and the control mAbs 2A10 and mGO53 at the indicated concentrations (n = 3–4).

E Pf sporozoite hepatocyte traversal inhibitory capacity of the indicated C-CSP reactive, C-CSP cross-reactive (1961), and control (2A10, mGO53 and anti-NANP 317; Oyen *et al*, 2017) mAbs at the indicated concentrations (n = 3–6).

F Capacity of the indicated passively-transferred antibodies (100 μg) to protect mice from parasitemia after the bite of three PbPfCSP(mCherry)-infected mosquitoes (n = 10 for mAb 317; Oyen *et al*, 2017, n = 8 for mAb 1961, and n = 9 for mAb 1710; Scally *et al*, 2018). Data show the percentage of parasite-free mice in two independent experiments (only mice with detectable serum IgG concentrations of the indicated mAbs at the time of the challenge are shown; Fig EV4C). Groups marked with the same letter were not statistically significantly different (Mantel–Cox log-rank test, see also Appendix Table S5).

Data information: Circles in (A, C–E) indicate independent biological replicates.
Source data are available online for this figure.

binding was only observed for the C-linker specific mAb 3764 at high (100 μg/ml) but not at low (1 μg/ml) concentration. However, the cross-reactive mAb 1961 strongly bound to the sporozoites similar to the positive control anti-repeat mAb 2A10 (Fig 4B–D). In agreement with their inability to bind live sporozoites, none of the anti-C-CSP specific antibodies showed Pf sporozoite traversal-inhibitory capacity even at a high concentration (100 μg/ml), including the high-affinity C-linker specific mAb 3764, which weakly bound sporozoites (Fig 4E). Only the repeat and junction cross-reactive mAb 1961 showed *in vitro* sporozoite-inhibitory activity in the range of mAbs 2A10 and 317, a high affinity human anti-NANP mAb (Oyen *et al*, 2017; Triller *et al*, 2017). Nevertheless, the capacity of mAb 1961 to protect from the development of parasitemia *in vivo* after passive transfer in mice was significantly lower than that of mAb 317 and similar to mAb 1710 (Figs 4F and EV4C; Appendix Table S5).

Thus, regardless of their fine epitope conformational specificity and affinity, anti-C-CSP antibodies lacked measurable sporozoite-binding capacity and *in vitro* parasite-inhibitory activity even at 100-fold higher concentrations than anti-repeat and cross-reactive mAbs. Only a single C-CSP reactive mAb with cross-reactivity to the repeat and N-terminal junction mediated parasite inhibition *in vitro* but was less efficacious than the potent anti-repeat mAb 317 *in vivo*.

mAb 3764 binds a DPN core sequence in $PfCSP_{281–294}$

The C-linker specific mAb 3764 that weakly bound sporozoites was the only C-CSP specific mAb (Fig EV5A) encoded by *IGHV3-33*, a gene which is associated with germline NANP reactivity and also encodes for antibodies with cross-reactivity to NPDP and NVDP motifs in the PfCSP N-terminal junction (Murugan *et al*, 2020). The C-CSP target peptide P2 of mAb 3764 (QGHNMPN**DPN**RNVD; Figs 3B and EV3B) showed sequence similarity with the N-terminal junction (NP**DPN**AN). To define the molecular basis for the specific recognition of the C-terminal linker region but not the N-terminal

 © 2023 The Authors

junction, we solved the crystal structure of the 3764 Fab in complex with P2 to 2.36 Å resolution (Appendix Table S6). The 3764 Fab recognizes its epitope with all complementarity-determining regions (CDRs) except Igκ CDR2 (KCDR2, Fig 5A). IgH CDR (HCDR) 2 and 3 form two hydrogen bonds with the core binding motif DPN (Fig 5A, inset I) contributing 274.7 $Å^2$ of buried surface area (BSA). Comparison of this binding mode to that of mAb 4498, a VH3-33 mAb that binds to the N-terminal junction (peptide NANPNVDPNANP; PDB ID: 6ULF; Murugan *et al*, 2020) shows that both mAbs recognize the DPN core motif in a highly similar disposition (whole atom RMSD of 0.24 Å; Fig EV5B and C).

In periphery of the DPN core motif, the surrounding C-CSP P2 residues Met 285 (M285), Asp 287 (N287) and Arg 291 (R291) partake in important interactions: M285 contributes 160.3 $Å^2$ of BSA at the interface with HCDR2 and Igκ CDR (KCDR) 3 (Fig 5A, inset II); N287 (Fig 5A, inset III) is responsible for five hydrogen bonds with HCDR3, KCDR1 and KCDR3 and contributes 156.8 $Å^2$ of BSA; while R291 (Fig 5A, inset IV) contributes four hydrogen bonds with HCDR1 and 106.3 $Å^2$ of BSA. Sequence analysis revealed that mAb 3764 had undergone limited somatic hypermutation, resulting in residues H.N56Y and H.Y58S in the HCDR2, which form part of the peptide-binding interface and together contribute 70.8 $Å^2$ of BSA around M285 (Fig 5A, inset II).

Given the sequence similarities between the core DPN in the C-CSP and N-junction peptides (Fig 5B), we investigated the molecular basis for mAb 3764-binding specificity to C-CSP. Modeling was performed to mutate the important peripheral C-CSP peptide residues M285, N287 and R291 (see Fig 5A), which surround the DPN core, to their corresponding N-junction peptide residues, Gly, Pro and Ala, respectively (Fig 5B). While it does not contribute any hydrogen bonds, the replacement of M285 with Gly would result in a much smaller BSA in the mAb 3764 HCDR2 and KCDR3 (compare Fig 5A inset II to Fig 5B left panel). Replacing N287 with Pro would result in the loss of four of the five hydrogen bonds with HCDR3, KCDR1 and KCDR3, as well as introducing steric hindrance with KCDR1 (compare Fig 5A inset III to Fig 5B center panel). Finally, Ala in place of Arg in position 291 would result in a loss of three of the four hydrogen bonds with HCDR1 (compare Fig 5A inset IV to Fig 5B right panel). Taken together, the crystal structure resolving PfCSP residues 284–293 in complex with mAb 3764, and molecular modeling, helped to explain the specificity of mAb 3764 for C-CSP through interactions with the IgH ($PfCSP_{284–292}$) and Igκ chains ($PfCSP_{285–287}$ and $PfCSP_{289}$), and the lack of cross-reactivity with the DPN motif in the N-terminal junction.

Discussion

The characterization of our large panel of human mAbs provides insights in the gene usage characteristics, development, cellular origin, epitope specificity, binding mode and functional properties of C-CSP reactive antibodies. Similar to the strong association between NANP-repeat reactivity and *IGHV3-33/IGKV1-5* gene usage, (Imkeller *et al*, 2018; Murugan *et al*, 2018; Tan *et al*, 2018), our data establish a link between C-CSP-reactivity and *IGHV3-21* and *IGVL3-21 or IGVL3-1* gene usage for Th2R/Th3R specific antibodies, which might allow conclusions about the frequency of C-CSP-reactive cells based on Ig gene sequencing. The association between *IGHV3-21* gene usage and antibody specificity for the C-CSP α-TSR domain appears to be independent of whether the immune system responds to recombinant protein or whole sporozoites, since Ig genes encoding VH3-21 anti-C-CSP mAbs have also been cloned from B cells isolated from RTS,S/AS01 and PfSPZ CVac vaccinees (Scally *et al*, 2018; Beutler *et al*, 2022). Nevertheless, the high *IGHV3-21* frequency that we observed after immunization with 3 doses of the PfSPZ Vaccine (900,000 radiation attenuated PfSPZ administered on days 1, 8, and 29) compared to immunization with the chemo-attenuated PfSPZ-CVac (Mordmuller *et al*, 2017) with strong dominance of *IGHV3-33* (Imkeller *et al*, 2018; Murugan *et al*, 2018; Tan *et al*, 2018) suggests that radiation, dose, or dosing interval might influence the anti-sporozoite B cell response. The large number of VH3-21 and non-VH3-21 B cells with Th2R/Th3R specificity including many that expressed unmutated antibodies indicates that the naïve B cell repertoire contains potent anti-Th2R/Th3R precursors. The relative abundance of these cells might contribute to the apparent immunodominance of this epitope compared to the conserved RII+/CS.T3, which is targeted by antibodies with diverse gene combinations, and compared to non-α-TSR epitopes.

In contrast to antibodies against the α-TSR domain, antibodies with reactivity to the C-linker appear to be rare and target linear epitopes. The differences in abundance of these antibodies may be linked to differences in the frequency of naïve precursor B cells that recognize these epitopes and structural differences between the C-linker region and α-TSR domain or differences in epitope presentation and immunogenicity. Analysis of the crystal structure of mAb 3764 in complex with amino acid residues 284–293 provides first insights into how the C-linker domain is recognized by a non-cross-reactive human anti-PfCSP antibody. Despite sequence identity in the C-linker with the N-terminal junction, the structural data provides a molecular explanation for the specificity of mAb 3764 for C-CSP. Steric constraints and suboptimal interactions with residues surrounding the core preclude cross-reactivity with the DPN motif in the junction. The data support the notion that peripheral residues define the binding specificity and cross-reactivity of antibodies beyond recognition of the core binding motif.

Despite the relative abundance of germline-encoded α-TSR-reactive antibodies, B cells with this specificity seem to undergo efficient affinity maturation and IgG isotype switching in germinal center reactions. The relative abundance of IgG compared to IgM antibodies, higher numbers of selected somatic mutations in VH3-21 mAbs compared to VH3-33 mAbs, and enrichment of the affinity-increasing H.T33 mutation, suggests that epitope specificity is linked to differences in cell differentiation pathways or cell differentiation kinetics. The large number of NANP motifs in the central repeat domain might mediate stronger BCR cross-linking and B cell activation signals compared to the non-repeating epitopes in the PfCSP C terminus, thereby affecting B cell activation, differentiation and germinal center selection (Imkeller *et al*, 2018). Alternatively, and not mutually exclusively, structure rather than valency differences between the disordered and highly flexible central repeat region compared to the structured C-CSP domain may underlie these isotype and mutation-load differences (MacRaild *et al*, 2018). Future studies will have to define the molecular and cellular mechanisms and determine whether epitope-associated differences in affinity maturation between repeat and C-CSP antibodies are also seen in response to immunization with recombinant PfCSP and RTS,S/AS01

© 2023 The Authors

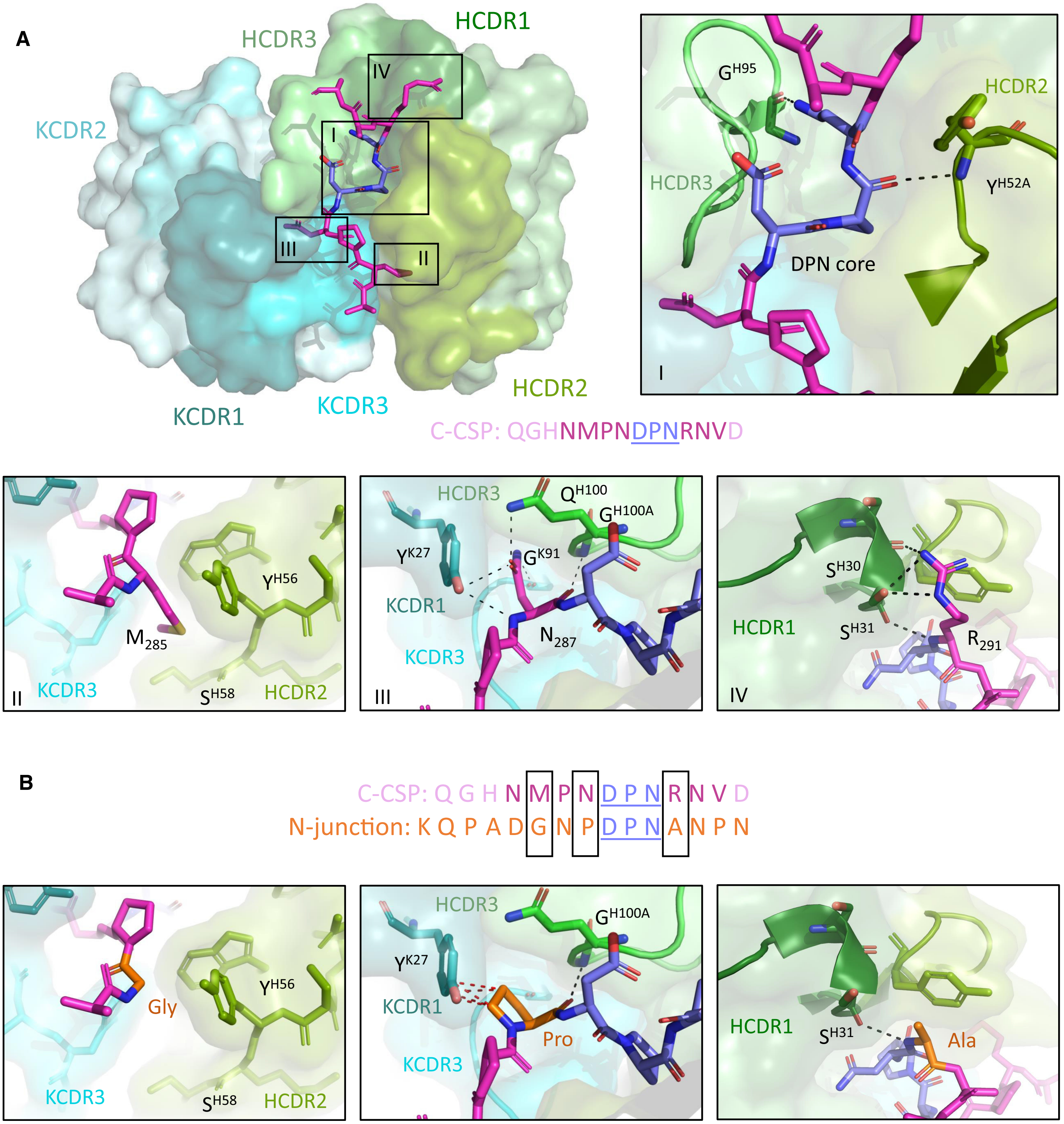

Figure 5. Molecular characterization of mAb 3764 recognition of the PfCSP.

A Crystal structure showing the variable regions of the C-linker specific mAb 3764 and P2, with the mAb 3764 heavy chain illustrated in green and the kappa chain in blue. HCDR1, 2 and 3 (dark, pea and light green, respectively) as well as KCDR1 and 3 (dark teal and aqua, respectively) contribute to epitope recognition, but KCDR2 (teal) does not. The central DPN core (deep blue) of the C-CSP peptide (magenta) interacts with HCDR2 and 3 similarly to other VH3-33 antibodies (inset I), forming hydrogen bonds (H-bonds, shown with black dotted lines) with HCDR2 and 3. C-CSP residues M285 (inset II), N287 (inset III) and R291 (inset IV) contribute substantial buried surface area (BSA) to the complex. (II) M285 does not contribute any H-bonds, but does contribute to BSA. N287 (III) and R291 (IV) are responsible for hydrogen bonding with HCDR3, KCDR1 and KCDR3, and with HCDR1, respectively.

B Despite significant sequence similarity in the region of the DPN core between the C-CSP peptide and the N-junction peptide (sequence shown in orange), mAb 3764 does not bind to the N-junction peptide. Molecular modeling replacing M285, N287 and R291 with Gly, Pro and Ala, respectively, reveals these alternate residues would result in a loss of hydrogen bonds, and reduced BSA and introduction of steric clashes (shown with red dotted lines).

© 2023 The Authors

or other antigens with repeating disordered domains that are characteristic of numerous parasite proteins.

Our study shows that the C-CSP of irradiated sporozoites is highly immunogenic and identifies linear target epitopes in the C-terminal linker and α-TSR domain. However, in assays with live Pf sporozoites none of the C-CSP epitopes seem to be the target of potent parasite-inhibitory antibodies. Even the C-linker specific mAb 3764, which showed weak parasite binding, lacked Pf-inhibitory activity at a hundred-fold higher concentration than antibodies against the central repeat domain, suggesting that modest C-CSP binding is not sufficient to mediate protection. Although the C-linker region may be slightly easier to reach for C-CSP mAbs than the α-TSR, which is directly anchored to the parasite surface, the central repeat and junction domains are clearly more accessible on the surface of live sporozoites. The direct comparison of C-CSP and NANP antibodies demonstrates that the almost complete inaccessibility of the PfCSP C-terminal domain strongly limits the direct parasite-inhibitory activity and potency of antibodies with specificity for this domain.

We have shown previously that passive transfer of the Th2R/Th3R-reactive mAb 1710 at high dose fails to protect mice from infection with PfCSP transgenic *P. berghei* parasites and the development of blood stage parasitemia (Scally *et al*, 2018; Murugan *et al*, 2020), findings that have recently been confirmed in an independent study (Wang *et al*, 2021). Since the epitopes of the Th2R/Th3R antibodies identified in this study overlap with the mAb 1710 target site (including many that share *VH3-21/Vλ3-21* Ig gene combinations), we do not expect these mAbs to exhibit any direct parasite-inhibitory activity *in vivo*. Given their lack of Pf sporozoite binding and *in vitro* parasite inhibition, we assume that the RII+/CS.T3 reactive antibodies would behave similarly. Some degree of *in vivo* protection from i.v. parasite challenge has recently been reported after high-dose passive transfer of the RII+/CS.T3 and the Th2R/Th3R-reactive mAbs 1512 and 236, respectively (Beutler *et al*, 2022). How the potency of these high-affinity mAbs compares to anti-repeat mAbs with similar binding strength has not been determined but given that mAbs 1512 and 236 target the same epitopes as the mAbs in our collection, we expect it to be overall low.

In response to RTS,S/AS01 immunization, anti-NANP and anti-C-terminus antibody titers correlate with protection (Chaudhury *et al*, 2016, 2021; Dobano *et al*, 2019). To what degree the C-CSP-reactive serum antibodies recognize C-CSP specifically or cross-react with the repeat and junction has not been determined. Therefore, it is unclear whether protection is associated with C-CSP specific or cross-reactive antibodies or whether C-CSP antibodies are simply a marker of the overall strength of the anti-parasite response, while protection is mediated by anti-repeat antibodies. Passive transfer of mAb 1961 shows that cross-reactive mAbs can mediate low levels of parasite inhibition *in vivo*. Such antibodies likely confer some degree of protection by binding to repeat and junction epitopes that are readily accessible on the surface of live sporozoites and not to the hidden C-CSP (Murugan *et al*, 2020). Cross-reactive anti-PfCSP mAbs have been shown to have overall higher affinity than antibodies that target the junction, repeat or C-CSP specifically (Murugan *et al*, 2020). Thus, protection seems to be linked to high affinity C-CSP cross-reactive rather than C-CSP specific antibodies. Nevertheless, we cannot exclude that C-CSP specific antibodies gain access to the C-terminus upon PfCSP binding by high affinity anti-NANP or junction antibodies. PfCSP shedding induced by anti-repeat/junction antibodies might uncover epitopes in the α-TSR domain enabling C-CSP-antibody binding and parasite clearance. Although synergistic effects of anti-repeat/junction and anti-C-CSP mAbs may not be easily detected in animal models with human monoclonal antibodies (Wang *et al*, 2021), antibody effector functions such as opsonization and phagocytosis or complement activation may contribute to parasite inhibition in humans. However, the high sequence diversity of the Th2R/Th3R domain among Pf parasite populations in natural settings will limit these indirect protective effects to antibodies against the conserved RII+/CS.T3 of the α-TSR domain, and to protection from parasites with high sequence similarity to the vaccine strain.

Future studies will need to determine whether efforts to design a second generation PfCSP vaccine might benefit from suppressing or even abrogating the humoral response against C-CSP, e.g. by boosting the anti-repeat and junction response. However, exclusion of the complete domain, especially of the highly immunodominant α-TSR, would eliminate the main T helper cell epitopes with likely strong negative effects on the quality and strength of the humoral response against the potent repeat and junction epitopes. Inclusion of linear peptide epitopes rather than the complete C-CSP may be sufficient to provide efficient T cell help without inducing non-protective humoral responses (Wahl *et al*, 2022). Alternatively, non-PfCSP T cell epitopes could substitute for the loss of T cell help and promote affinity maturation of the PfCSP-specific response (Ludwig *et al*, 2023).

In summary, with our mAb collection we demonstrate the lack of accessible C-CSP epitopes on live sporozoites as targets for potent parasite-inhibitory antibodies. Due to the high immunogenicity of the PfCSP C terminus, these observations are of direct relevance for the design of an improved PfCSP-based malaria vaccine.

Materials and Methods

Clinical samples

Serum and peripheral blood mononuclear cells (PBMCs, obtained by Ficoll density gradient centrifugation) were collected from malaria-naïve, healthy European volunteers who participated in the MAVACHE clinical trial and received three doses of 900,000 PfSPZ at 0, 1 and 4 weeks (Mordmuller *et al*, 2022). All study volunteers provided written informed consent. Ethical approval was obtained from the ethics committee of the medical faculty and the University Clinics of the University of Tübingen and regulatory approval by the Paul-Ehrlich-Institute. The study was conducted according to the principles of the Declaration of Helsinki. The study also conformed to the principles set out in the Department of Health and Human Services Belmont Report.

Cell lines

Human embryonic kidney HEK293T cells (Invitrogen, 0.3×10^6/ml) were cultured under rotating conditions (180 rpm) in 50 ml bioreactors with FreeStyle 293-F medium at 37°C and 8% CO_2. HC-04 cells (BEI Resources MRA-975; Sattabongkot *et al*, 2006) were cultured at 37°C and 5% CO_2 in HC-04 complete medium with 428.75 ml MEM (without L-glutamine), 428.75 ml F-12 Nutrient Mix (with

L-glutamine), 15 mM HEPES, 1.5 g/l NaHCO3, 2.5 mM L-glutamine, 10% FCS and 1% penicillin/streptomycin solution.

Bacteria

MAX efficiency DH10B™ (ThermoFisher scientific; Cat. No: 18297010) competent cells were grown in LB medium for cultivation and in terrific broth for plasmid purification. Bacterial shaker at 37°C and 180 rpm was used for cultivation.

Proteins and peptides

Biotinylated NF54 FL-CSP, non-biotinylated 7G8 FL-CSP and α-TSR protein were expressed in HEK293F cells and purified by HisTrap HP (Cytiva) and size exclusion chromatography (Superdex 200 Increase 10/300 GL, Cytiva). FL-CSP and C-CSP were expressed in *E.coli* by the EMBL protein expression and purification core facility (Heidelberg, Germany). N-junc, $NANP_5$, $NANP_{10}$, C-linker and the 14 overlapping peptides (P1-14) covering the complete NF54 PfCSP C-terminus were synthesized by PSL Peptide Specialty Laboratories GmbH (Heidelberg, Germany). Sequences of peptides and proteins are listed in Appendix Table S1.

Parasites, mosquitoes and mice

Anopheles coluzzii (Ngousso S1 strain; Harris *et al*, 2010) and transgenic *Anopheles gambiae* (*7b* strain; Pompon & Levashina, 2015) were maintained at 28°C 70–80% humidity 12/12 h day/night cycle. The *P. falciparum* NF54 clone (Pf) used in this study originated from Prof. Sauerwein's laboratory (RUMC, Nijmegen) and was regularly tested for *Mycoplasma* contamination. For *P. falciparum* infections, *A. coluzzii* mosquitoes were fed for 15 min via artificial midi feeders (Glass Instruments, The Netherlands) with NF54 gametocyte cultures and kept at 26°C 80% humidity 12/12 h day/night cycle in a secured BSL3 laboratory according to the national regulations (Landesamt für Gesundheit und Soziales, project number 297/13). Infected mosquitoes were offered an additional uninfected blood meal to boost sporozoite formation 8 days post infection (dpi). Sporozoites were dissected for traversal assay 5–7 days later as described below.

Anopheles gambiae 7b mosquitoes, an immunocompromised transgenic mosquito line derived from the G3 laboratory strain, were used for the production of transgenic *P. berghei* sporozoites expressing PfCSP and the reporter fluorescence protein mCherry (PbPfCSP(mCherry), Ludwig *et al*, 2023). Briefly, female CD1 mice (7–12-weeks-old) and female C57BL/6J mice (8-weeks-old) were bred in the MPIIB Experimental Animal Facility (Marienfelde, Berlin), housed in a pathogen-free animal facility in accordance with the German Animal Protection Law (§8 Tierschutzgesetz) and approved by the Landesamt für Gesundheit und Soziales (LAGeSo), Berlin, Germany (project numbers 368/12 and H0335/17). *A. gambiae 7b* mosquitoes were fed on PbPfCSP(mCherry)-infected female CD1 mice 3 days post passage (0.1–0.8% gametocytemia) for 30–45 min, and kept at 20°C 80% humidity 12/12 h day/night cycle. Infected mosquitoes were offered an additional uninfected blood meal to boost sporozoite formation 7 dpi, and after 11 days were used for C57BL/6J mice challenge experiments and live sporozoite FACS staining as described below.

Flow cytometry and isolation of PfCSP-specific memory B cells

Frozen PBMCs were thawed, washed with RPMI 1640 (Gibco) and incubated with full-length biotinylated NF54 PfCSP in the presence of the following mouse anti-human antibodies at the indicated dilutions: CD27-phycoerythrin (PE) (Clone ID: M-T271; Cat. No: 555441) at 1:5, CD38- Brilliant Violet 605 (BV605) (Clone ID: HB7; Cat. No: 562665) at 1:20, IgG-BV510 (Clone ID: G18-145; Cat. No: 563247) at 1:20, IgM-BV421 (Clone ID: G20-127; Cat. No: 562618) at 1:20, IgD-Allophycocyanin-H7 (APC-H7) (Clone ID: IA6-2; Cat. No: 561305) at 1:20 (all from BD Biosciences), CD19-BV786 (Clone ID: HIB19; Cat. No: 302240) at 1:10 and CD21- PE-Cy7 (Clone ID: Bu32; Cat. No: 354912) at 1:20 (both from BioLegend). Biotin was detected using streptavidin FITC (Invitrogen; Cat. No: 11-4317-87) at 1:1,000 dilution. 7-Aminoactinomycin D (7-AAD) (Invitrogen; Cat No: A1310) at 1:400 was used as a marker for dead cells. Single-cell sorting of 7AAD−PfCSP+CD19+ B cells gated positive for either IgG or CD27 as PfCSP memory B cells and of 7AAD−CD38+CD27+CD19+ cells as plasmablasts was performed using a FACS Aria II (BD Biosciences) with FACSDiva software (version 8.0.1) using the index sort option. Data were analyzed using FlowJo v.10.0.8 (Tree Star).

Ig gene amplification and sequence analysis

A high-throughput robotic platform was used to amplify the *IGH*, *IGK* and *IGL* loci of Ig genes by RT-PCR as previously reported (Wardemann & Busse, 2019). Briefly, after cDNA synthesis with random hexamers, a nested polymerase chain reaction (PCR) with barcoded primers in the second PCR was used to amplify the Ig heavy and light chain genes. Amplicons were pooled, purified, and sequenced using the MiSeq 2 × 300 base pair (bp) paired-end sequencing platform (Illumina). Integrated indexed flow cytometry and sequence data integration as well as Ig gene annotation was performed using sciReptor version 1.0-6-g034c8ae (Busse *et al*, 2014; Imkeller *et al*, 2016). Downstream sequence analyses and data visualization were performed using GraphPad Prism (version 9.12) and R 4.2.2 and ggplot2.

Cloning of Ig genes and recombinant monoclonal antibodies production

Cloning of the Ig genes and recombinant antibody production were performed as previously described (Wardemann & Busse, 2019). Ig genes were amplified with V- and J-specific primers containing restriction sites. The amplicons were cloned into human *IGHG1* or *IGKC* or *IGLC* expression vectors (Addgene number 80795, 80796 and 99575, respectively). After co-transfection of the Ig heavy and light encoding plasmid DNA into HEK293T cells (Invitrogen), the recombinant monoclonal antibodies (mAbs) were purified from the supernatant with protein G beads (GE Healthcare). Subsequent experiments with the mAbs, including functional assays, were performed in an unblinded manner.

Enzyme-linked immunosorbent assay (ELISA)

ELISAs were performed as described (Triller *et al*, 2017; Murugan *et al*, 2020). In brief, 384-well polystyrene plates (Corning) with

© 2023 The Authors

high binding capacity were coated overnight (O/N) at 4°C with full-length PfCSP (0.4 μg/ml), N-junc, $NANP_{10}$ (2 μg/ml), C-CSP (1 μg/ml), Insulin (10 μg/ml; Sigma Aldrich), lipopolysaccharide (LPS; 10 μg/ml; Sigma Aldrich) and double-stranded DNA (dsDNA; 20 μg/ml; Sigma Aldrich) in PBS. The plates were washed 3 times with PBS-T (0.05% Tween20 in PBS) using a Tecan plate washer. ELISA plates were blocked at RT for 1 h with 4% BSA in PBS (serum ELISA), 1% BSA in PBS (mAb PfCSP ELISA) or blocking buffer (1× PBS, 0.05% (v/v) Tween20, 1 mM EDTA; mAb polyreactivity ELISA). Serum samples serially diluted at an initial dilution of 1:200 in 1% BSA with PBS, or mAbs at an initial concentration of 4 μg/ml (PfCSP ELISA) or 1 μg/ml (polyreactivity ELISA) in 1× PBS were loaded on the plate and incubated at RT for 1.5 h. Antibodies were detected with goat anti-human IgG secondary antibody coupled to horseradish peroxidase (HRP) (Jackson Immuno Research; Cat No: 109-035-098) diluted at 1:1,000 in blocking buffer (1× PBS, 0.05% (v/v); Tween20, 1 mM EDTA) and then detected with 2,2′-azino-bis-(3-ethylbenzothiazoline-6-sulfonic acid) diammonium (ABTS) substrate (Roche Diagnostics; Cat No: 11112422001) diluted at 1:1,000 in H_2O_2. Optical density (OD) at 405 nm was determined using an M1000Pro plate reader (Tecan). Area under the curve (AUC) values were calculated using GraphPad Prism 9.1.2. Serum from placebo recipients was used as a negative control in serum ELISAs. mAbs 2A10 (Triller *et al*, 2017) and mGO53 (Wardemann *et al*, 2003) were used as positive and negative controls, respectively, in PfCSP ELISAs and mAbs ED38 (Meffre *et al*, 2004) and mGO53 as positive and negative controls, respectively, in polyreactivity ELISAs.

Blocking ELISA

Blocking ELISA was performed as described above with the following modifications. After 1.5 h incubation with the test mAbs (starting concentration of 64 μg/ml in 1× PBS and 1:2 dilution) at room temperature, biotinylated mAb 1710 (Scally *et al*, 2018) or mAb 1512 (Beutler *et al*, 2022) at 0.5 μg/ml was added to compete for an additional 15 min at room temperature. mAb 1710 or mAb 1512 binding was detected with streptavidin-HRP diluted at 1:1,000 in blocking buffer (1× PBS, 0.05% (v/v) Tween20, 1 mM EDTA) and then detected with 2,2′-azino-bis-(3-ethylbenzothiazoline-6-sulfonic acid) diammonium (ABTS) substrate (Roche Diagnostics) diluted at 1:1,000 in H_2O_2. The optimal concentrations of the biotinylated 1710 and 1512 were determined by measuring their ability to self-block their non-biotinylated versions over a range of concentrations.

Surface Plasmon resonance (SPR)

SPR measurements were performed using a Biacore T200 (GE Health-care) instrument docked with an S CM5 series sensor chip (GE Healthcare), as described (Murugan *et al*, 2018). In brief, anti-human IgG antibodies were immobilized on the chip using an amine coupling-based human antibody Fab capture kit. Hepes (10 mM) with 150 mM NaCl at pH 7.4 was used as running buffer. Equal concentrations of the sample antibody and isotype control (mGO53; Wardemann *et al*, 2003) were captured in the sample and reference flow cells, respectively. Running buffer was injected at a rate of 10 μl/min for 20 min to stabilize the flow cells. C-CSP peptide at 0.015, 0.09, 0.55, 3.3 and 20 μM in running buffer was injected at a rate of 30 μl/min. The flow cells were regenerated with 3 M $MgCl_2$. Data were fitted by steady-state kinetic analysis using BIACORE T200 software V2.0.

mAb 3764 Fab production

mAb 3764 Ig heavy and light chain variable regions were cloned into custom pcDNA3.4 expression vectors upstream of human IgK constant and IgG1-C_H1 domains, and transiently co-transfected into HEK293F cells. The resulting Fab was purified by Kappa-select affinity chromatography (Cytiva), cation exchange chromatography (MonoS; Cytiva), and size exclusion chromatography (Superdex 200 Increase 10/300 GL; Cytiva).

Crystallization and structure determination

Purified 3764 Fab and PfCSP C-terminal linker peptide ($PfCSP_{281-294}$) were mixed in a 1:3 molar ratio; and, in sparse matrix screening, crystals of the complex emerged from a 1:1 ratio mix with 19% (v/v) isopropanol, 19% (w/v) PEG 4000, 5% (v/v) glycerol, 0.095 M sodium citrate, pH 5.6. Crystals did not require cryopreservation before being flash frozen in liquid nitrogen. Data was collected at the 23-ID-B beamline at the Advanced Photon Source and manually processed using XDS (Kabsch, 2010). Molecular replacement using the heavy and light chains from the 4498 Fab (PDB ID: 6O24) was carried out in Phaser (McCoy *et al*, 2007). Iterative refinement was performed in Coot (Emsley *et al*, 2010) and phenix.refine (Adams *et al*, 2010). All software were accessed through SBGrid (Morin *et al*, 2013). The structure has been deposited in the PDB and is accessible under ID: 8FG0.

Pf sporozoite hepatocyte traversal assay *in vitro*

Traversal assays were performed as previously described (Triller *et al*, 2017; Murugan *et al*, 2018). Briefly, Pf sporozoites were isolated from the mosquito salivary glands 13–15 dpi in complete HC-04 medium, pre-incubated with mAb at the indicated concentrations for 30 min on ice and added to the human hepatocyte cell line (HC-04, BEI Resources MRA-975, Sattabongkot *et al*, 2006) for 2 h at 37°C and 5% CO_2 in the presence of 0.5 mg/ml dextran-rhodamine (Molecular Probes). Untreated Pf sporozoites were used to measure maximum traversal capacity. A chimeric version of mAb 2A10 (Zavala *et al*, 1983) with human IgG1 constant region (Triller *et al*, 2017), and mGO53 (Wardemann *et al*, 2003) were used as positive and negative controls, respectively. Cells were trypsinized, washed and fixed with 1% PFA/PBS before measuring dextran positivity using a FACS LSRFortessa instrument and FACSDiva software (BD Biosciences). Data analysis was performed by subtracting background (dextran positivity of cells treated with uninfected mosquito salivary gland material) and normalizing to maximum Pf traversal capacity (dextran positivity of cells without mAbs) using FlowJo V.10.8.2 (Tree Star).

Pb-PfCSP(mCherry) live sporozoite flow cytometry

Plasmodium berghei PbPfCSP(mCherry) (Ludwig *et al*, 2023) sporozoites were isolated from mosquito salivary glands 18 dpi in HC-04

© 2023 The Authors

complete medium, incubated for 30 min on ice in siliconized tubes with the indicated concentrations of mAbs (150,000 sporozoites in total volume of 100 µl 1% FCS/PBS). Sporozoites were washed with 1.5 ml of 1% FCS/PBS by centrifugation at 9,300 *g* for 4 min at 4°C. The sporozoite pellet was resuspended in 100 µl 1% FCS/PBS containing anti-human IgG1-Cy5 (clone HP6001, 2 µg/ml, Central Laboratory Facility Deutsches Rheuma-Forschungszentrum, Berlin) and incubated on ice for 30 min. The sporozoite suspension was washed again, and the pellet was re-suspended in 200 µl 1% FCS/PBS. Sporozoites stained with antibodies were detected by endogenous mCherry expression and analyzed for Cy5 signal in a FACS LSRFortessa instrument with FACSDiva software (BD Biosciences). Salivary gland material from uninfected mosquitoes was used as sporozoite gating control (Fig EV4A). Data were analyzed using FlowJo V.10.8.2 (Tree Star).

Plasmodium berghei PbPfCSP(mCherry) mouse challenge model by mosquito bites

Female C57BL/6J mice were passively immunized by intraperitoneal injection of 100 µg mAb in 100–200 µl PBS. Mice were randomly assigned to different groups. After 20 h, mice were exposed to the bites of three PbPfCSP(mCherry) salivary gland-infected mosquitoes as described elsewhere (Ludwig *et al*, 2023). Briefly, PbPfCSP(mCherry)-infected mosquitoes were put to sleep on ice at 17 dpi and selected for mCherry signal in the salivary glands under a fluorescence stereo microscope (Leica, M205 FA). Single salivary gland-positive mosquitoes were transferred into individual containers and starved overnight. The next day (18 dpi), anesthetized naïve C57BL/6J mice were exposed for 10 min to three bites of the selected PbPfCSP(mCherry)-infected mosquitoes. Peripheral whole blood was collected from the submandibular vein 2–3 h after challenge to obtain sera. Serum mAb concentrations were determined by ELISA. Mice with undetectable levels of mAbs in the serum were excluded from the analysis. From days 3 to 7 and on day 10 post mosquito bite, parasitemia (mCherry-positive red blood cells/total red blood cells) was measured by flow cytometry (LSRFortessa, BD Biosciences), and confirmed by Giemsa-stained thin blood smears. All infected mice were euthanized on day 7 post mosquito bite, before the occurrence of malaria symptoms. FACS data were analyzed by FlowJo V.10.8.2 and the pre-patency period was declared on the first day when parasitemia values were above the background signal of negative mice.

Statistics

Statistical analysis was done with GraphPad Prism (version 9.12) using a two-tailed non-parametric Mann–Whitney test assuming non-normal distribution of the data and two-tailed Mantel-Cox log-rank test for the *in vivo* experiment, as described in the figure legends. *In vitro* and *in vivo* experiments were performed in at least two independent technical and biological replicates, respectively. Mice were randomly assigned to their respective experimental groups from the same batches. No experiment was done blindly. P values lower than 0.05 were regarded as statistically significant, with asterisks used to indicate the level of significance (*$P < 0.05$; **$P < 0.01$; ***$P < 0.001$; ****$P < 0.0001$), as described in the figure legends.

The paper explained

Problem

RTS,S/AS01, the only available malaria vaccine shows limited efficacy. RTS,S/AS01 induces antibodies against the central repeat domain and C terminus of *Plasmodium falciparum* circumsporozoite protein (PfCSP). Anti-repeat antibodies mediate protection from the infection but the role of antibodies against the C-terminal domain (C-CSP), which is immunodominant in recombinant PfCSP remains unclear.

Results

By generating a large panel of human monoclonal PfCSP antibodies, we demonstrate that C-CSP specific antibodies lack parasite-inhibitory activity independently of their C-CSP fine epitope specificity. We show that high-affinity binding C-CSP specific antibodies are part of the naïve human B cell repertoire and undergo efficient affinity maturation. The most abundant C-CSP specific antibodies targeted polymorphic epitopes in the α-TSR domain and were frequently encoded by *IGHV3-21* genes.

Impact

The apparent lack of protective antibody epitopes in the PfCSP C terminus suggests that the design of a second generation PfCSP-based malaria vaccine may benefit from focusing the humoral immune response on the repeat domain and junction while avoiding non-protective antibody responses against C-CSP.

Data availability

The datasets and material that were generated in this study can be obtained from the corresponding authors under standard transfer agreements. Datasets are deposited on ENA with accession ID: PRJEB60486, Zenodo DOI: https://doi.org/10.5281/zenodo.7708746. The crystal structure has been deposited in the Protein Data Bank at www.rcsb.org under PDB ID code 8FG0 (http://identifiers.org/pdb/8FG0).

Expanded View for this article is available online.

Acknowledgements

The authors thank Dorien Foster, Julia Gärtner, Christine Niesik, Claudia Winter (DKFZ) and Hadenal Gordon, Cornelia Kreschel, Manuela Andres, Daniel Eyermann and Liane Spohr (Max Planck Institute for Infection Biology, Berlin) for experimental assistance, Christian E. Busse (DKFZ) for bioinformatic support, the EMBL Protein Expression and Purification Core Facility and the DKFZ/ EMBL/Heidelberg University Chemical Biology Core Facility, especially P. Sehr, and the Deutsches Rheuma-Forschungszentrum Flow Cytometry Core Facility for technical assistance and services. The work was supported by the Bill and Melinda Gates Foundation (OPP1179906; J.P.J, H.W. and E.A.L.), by the CIFAR Azrieli Global Scholar program (J.P.J.), the Ontario Early Researcher Award program (J.P.J.), and the Canada Research Chair program (J.P.J.). C.B.A was supported by a by Hospital for Sick Children Restracomp Postdoctoral Fellowship and a Banting Postdoctoral Fellowship. X-ray diffraction experiments were performed at GM/CA@APS, which has been funded in whole or in part with federal funds from the National Cancer Institute (ACB-12002) and the National Institute of General Medical Sciences (AGM-12006). The Eiger 16 M detector at GM/CA-XSD was funded by NIH grant S10 OD012289. This research used resources of the Advanced Photon Source, a US Department of

© 2023 The Authors

Energy (DOE) Office of Science user facility operated for the DOE Office of Science by Argonne National Laboratory under contract DE-AC02-06CH11357. Manufacture of PfSPZ Vaccine was funded in part by the National Institute of Allergy and Infectious Diseases of the National Institutes of Health under SBIR award numbers 5R44AI058375 and 5R44AI055229.

Author contributions

Opeyemi Ernest Oludada: Formal analysis; investigation; visualization; writing – original draft; writing – review and editing. **Giulia Costa:** Formal analysis; investigation; visualization; writing – original draft; writing – review and editing. **Clare Burn Aschner:** Formal analysis; investigation; visualization; writing – original draft; writing – review and editing. **Anna S Obraztsova:** Data curation; formal analysis; visualization. **Katherine Prieto:** Investigation. **Caterina Canetta:** Investigation. **Stephen L Hoffman:** Resources. **Peter G Kremsner:** Resources. **Benjamin Mordmüeller:** Resources. **Rajagopal Murugan:** Conceptualization; writing – review and editing. **Jean-Philippe Julien:** Conceptualization; funding acquisition; writing – original draft; writing – review and editing. **Elena A Levashina:** Conceptualization; funding acquisition; writing – original draft; writing – review and editing. **Hedda Wardemann:** Conceptualization; funding acquisition; writing – original draft; writing – review and editing.

Disclosure and competing interests statement

The authors declare the following competing interests: S.L.H is a salaried employee of Sanaria Inc. and has a financial interest in Sanaria Inc. All other authors declare no financial or commercial conflict of interest.

For more information

https://www.who.int/teams/global-malaria-programme/reports/world-malaria-report-2022
https://www.mpiib-berlin.mpg.de/research/vector_biology
https://lab.research.sickkids.ca/julien/
https://www.dkfz.de/de/b-zell-immunologie/index.php

References

Adams PD, Afonine PV, Bunkoczi G, Chen VB, Davis IW, Echols N, Headd JJ, Hung LW, Kapral GJ, Grosse-Kunstleve RW *et al* (2010) PHENIX: a comprehensive python-based system for macromolecular structure solution. *Acta Crystallogr D Biol Crystallogr* 66: 213 – 221

Agnandji ST, Lell B, Soulanoudjingar SS, Fernandes JF, Abossolo BP, Conzelmann C, Methogo BG, Doucka Y, Flamen A, Mordmuller B *et al* (2011) First results of phase 3 trial of RTS,S/AS01 malaria vaccine in African children. *N Engl J Med* 365: 1863 – 1875

Aragam NR, Thayer KM, Nge N, Hoffman I, Martinson F, Kamwendo D, Lin FC, Sutherland C, Bailey JA, Juliano JJ (2013) Diversity of T cell epitopes in *Plasmodium falciparum* circumsporozoite protein likely due to protein-protein interactions. *PLoS One* 8: e62427

Asante KP, Abdulla S, Agnandji S, Lyimo J, Vekemans J, Soulanoudjingar S, Owusu R, Shomari M, Leach A, Jongert E *et al* (2011) Safety and efficacy of the RTS,S/AS01E candidate malaria vaccine given with expanded-programme-on-immunisation vaccines: 19 month follow-up of a randomised, open-label, phase 2 trial. *Lancet Infect Dis* 11: 741 – 749

Beutler N, Pholcharee T, Oyen D, Flores-Garcia Y, MacGill RS, Garcia E, Calla J, Parren M, Yang L, Volkmuth W *et al* (2022) A novel CSP C-terminal epitope targeted by an antibody with protective activity against *Plasmodium falciparum*. *PLoS Pathog* 18: e1010409

Bojang KA, Milligan PJ, Pinder M, Vigneron L, Alloueche A, Kester KE, Ballou WR, Conway DJ, Reece WH, Gothard P *et al* (2001) Efficacy of RTS,S/AS02 malaria vaccine against *Plasmodium falciparum* infection in semi-immune adult men in The Gambia: a randomised trial. *Lancet* 358: 1927 – 1934

Busse CE, Czogiel I, Braun P, Arndt PF, Wardemann H (2014) Single-cell based high-throughput sequencing of full-length immunoglobulin heavy and light chain genes. *Eur J Immunol* 44: 597 – 603

Casares S, Brumeanu TD, Richie TL (2010) The RTS,S malaria vaccine. *Vaccine* 28: 4880 – 4894

Cawlfield A, Genito CJ, Beck Z, Bergmann-Leitner ES, Bitzer AA, Soto K, Zou X, Hadiwidjojo SH, Gerbasi RV, Mullins AB *et al* (2019) Safety, toxicity and immunogenicity of a malaria vaccine based on the circumsporozoite protein (FMP013) with the adjuvant army liposome formulation containing QS21 (ALFQ). *Vaccine* 37: 3793 – 3803

Chaudhury S, Ockenhouse CF, Regules JA, Dutta S, Wallqvist A, Jongert E, Waters NC, Lemiale F, Bergmann-Leitner E (2016) The biological function of antibodies induced by the RTS,S/AS01 malaria vaccine candidate is determined by their fine specificity. *Malar J* 15: 301

Chaudhury S, MacGill RS, Early AM, Bolton JS, King CR, Locke E, Pierson T, Wirth DF, Neafsey DE, Bergmann-Leitner ES (2021) Breadth of humoral immune responses to the C-terminus of the circumsporozoite protein is associated with protective efficacy induced by the RTS,S malaria vaccine. *Vaccine* 39: 968 – 975

Coppi A, Pinzon-Ortiz C, Hutter C, Sinnis P (2005) The *Plasmodium* circumsporozoite protein is proteolytically processed during cell invasion. *J Exp Med* 201: 27 – 33

Dame JB, Williams JL, McCutchan TF, Weber JL, Wirtz RA, Hockmeyer WT, Maloy WL, Haynes JD, Schneider I, Roberts D *et al* (1984) Structure of the gene encoding the immunodominant surface antigen on the sporozoite of the human malaria parasite *Plasmodium falciparum*. *Science* 225: 593 – 599

Dobano C, Sanz H, Sorgho H, Dosoo D, Mpina M, Ubillos I, Aguilar R, Ford T, Diez-Padrisa N, Williams NA *et al* (2019) Concentration and avidity of antibodies to different circumsporozoite epitopes correlate with RTS,S/AS01E malaria vaccine efficacy. *Nat Commun* 10: 2174

Doud MB, Koksal AC, Mi LZ, Song G, Lu C, Springer TA (2012) Unexpected fold in the circumsporozoite protein target of malaria vaccines. *Proc Natl Acad Sci U S A* 109: 7817 – 7822

Emsley P, Lohkamp B, Scott WG, Cowtan K (2010) Features and development of Coot. *Acta Crystallogr D Biol Crystallogr* 66: 486 – 501

Espinosa DA, Gutierrez GM, Rojas-Lopez M, Noe AR, Shi L, Tse SW, Sinnis P, Zavala F (2015) Proteolytic cleavage of the *Plasmodium falciparum* circumsporozoite protein is a target of protective antibodies. *J Infect Dis* 212: 1111 – 1119

Gandhi K, Thera MA, Coulibaly D, Traore K, Guindo AB, Doumbo OK, Takala-Harrison S, Plowe CV (2012) Next generation sequencing to detect variation in the *Plasmodium falciparum* circumsporozoite protein. *Am J Trop Med Hyg* 86: 775 – 781

Genito CJ, Beck Z, Phares TW, Kalle F, Limbach KJ, Stefaniak ME, Patterson NB, Bergmann-Leitner ES, Waters NC, Matyas GR *et al* (2017) Liposomes containing monophosphoryl lipid A and QS-21 serve as an effective adjuvant for soluble circumsporozoite protein malaria vaccine FMP013. *Vaccine* 35: 3865 – 3874

Gordon DM, McGovern TW, Krzych U, Cohen JC, Schneider I, LaChance R, Heppner DG, Yuan G, Hollingdale M, Slaoui M *et al* (1995) Safety, immunogenicity, and efficacy of a recombinantly produced *Plasmodium falciparum* circumsporozoite protein-hepatitis B surface antigen subunit vaccine. *J Infect Dis* 171: 1576 – 1585

Gupta NT, Vander Heiden JA, Uduman M, Gadala-Maria D, Yaari G, Kleinstein SH (2015) Change-O: a toolkit for analyzing large-scale B cell immunoglobulin repertoire sequencing data. *Bioinformatics* 31: 3356 – 3358

Harris C, Lambrechts L, Rousset F, Abate L, Nsango SE, Fontenille D, Morlais I, Cohuet A (2010) Polymorphisms in *Anopheles gambiae* immune genes associated with natural resistance to *Plasmodium falciparum*. *PLoS Pathog* 6: e1001112

Herrera R, Anderson C, Kumar K, Molina-Cruz A, Nguyen V, Burkhardt M, Reiter K, Shimp R Jr, Howard RF, Srinivasan P *et al* (2015) Reversible conformational change in the *Plasmodium falciparum* Circumsporozoite protein masks its adhesion domains. *Infect Immun* 83: 3771 – 3780

Hutter JN, Robben PM, Lee C, Hamer M, Moon JE, Merino K, Zhu L, Galli H, Quinn X, Brown DR *et al* (2022) First-in-human assessment of safety and immunogenicity of low and high doses of *Plasmodium falciparum* malaria protein 013 (FMP013) administered intramuscularly with ALFQ adjuvant in healthy malaria-naive adults. *Vaccine* 40: 5781 – 5790

Imkeller K, Arndt PF, Wardemann H, Busse CE (2016) sciReptor: analysis of single-cell level immunogobulin repertoires. *BMC Bioinformatics* 17: 67

Imkeller K, Scally SW, Bosch A, Marti GP, Costa G, Triller G, Murugan R, Renna V, Jumaa H, Kremsner PG *et al* (2018) Antihomotypic affinity maturation improves human B cell responses against a repetitive epitope. *Science* 360: 1358 – 1362

Kabsch W (2010) XDS. *Acta Crystallogr D Biol Crystallogr* 66: 125 – 132

Kisalu NK, Idris AH, Weidle C, Flores-Garcia Y, Flynn BJ, Sack BK, Murphy S, Schon A, Freire E, Francica JR *et al* (2018) A human monoclonal antibody prevents malaria infection by targeting a new site of vulnerability on the parasite. *Nat Med* 24: 408 – 416

Ludwig J, Scally SW, Costa G, Hoffmann S, Murugan R, Lossin J, Prieto K, Obraztcova A, Lobeto N, Franke-Fayard B *et al* (2023) Glycosylated nanoparticle-based PfCSP vaccine confers long-lasting antibody responses and sterile protection in mouse malaria model. *NPJ Vaccines* 8: 52

MacRaild CA, Seow J, Das SC, Norton RS (2018) Disordered epitopes as peptide vaccines. *Pept Sci* 110: e24067

McCoy AJ, Grosse-Kunstleve RW, Adams PD, Winn MD, Storoni LC, Read RJ (2007) Phaser crystallographic software. *J Appl Crystallogr* 40: 658 – 674

McCutchan TF, Kissinger JC, Touray MG, Rogers MJ, Li J, Sullivan M, Braga EM, Krettli AU, Miller LH (1996) Comparison of circumsporozoite proteins from avian and mammalian malarias: biological and phylogenetic implications. *Proc Natl Acad Sci U S A* 93: 11889 – 11894

Meffre E, Schaefer A, Wardemann H, Wilson P, Davis E, Nussenzweig MC (2004) Surrogate light chain expressing human peripheral B cells produce self-reactive antibodies. *J Exp Med* 199: 145 – 150

Mordmuller B, Surat G, Lagler H, Chakravarty S, Ishizuka AS, Lalremruata A, Gmeiner M, Campo JJ, Esen M, Ruben AJ *et al* (2017) Sterile protection against human malaria by chemoattenuated PfSPZ vaccine. *Nature* 542: 445 – 449

Mordmuller B, Sulyok Z, Sulyok M, Molnar Z, Lalremruata A, Calle CL, Bayon PG, Esen M, Gmeiner M, Held J *et al* (2022) A PfSPZ vaccine immunization regimen equally protective against homologous and heterologous controlled human malaria infection. *NPJ Vaccines* 7: 100

Morin A, Eisenbraun B, Key J, Sanschagrin PC, Timony MA, Ottaviano M, Sliz P (2013) Collaboration gets the most out of software. *Elife* 2: e01456

Murugan R, Buchauer L, Triller G, Kreschel C, Costa G, Pidelaserra Marti G, Imkeller K, Busse CE, Chakravarty S, Sim BKL *et al* (2018) Clonal selection drives protective memory B cell responses in controlled human malaria infection. *Sci Immunol* 3: eaap8029

Murugan R, Scally SW, Costa G, Mustafa G, Thai E, Decker T, Bosch A, Prieto K, Levashina EA, Julien JP *et al* (2020) Evolution of protective human antibodies against *Plasmodium falciparum* circumsporozoite protein repeat motifs. *Nat Med* 26: 1135 – 1145

Nardin EH, Nussenzweig V, Nussenzweig RS, Collins WE, Harinasuta KT, Tapchaisri P, Chomcharn Y (1982) Circumsporozoite proteins of human malaria parasites *Plasmodium falciparum* and *Plasmodium vivax*. *J Exp Med* 156: 20 – 30

Olotu A, Lusingu J, Leach A, Lievens M, Vekemans J, Msham S, Lang T, Gould J, Dubois MC, Jongert E *et al* (2011) Efficacy of RTS,S/AS01E malaria vaccine and exploratory analysis on anti-circumsporozoite antibody titres and protection in children aged 5–17 months in Kenya and Tanzania: a randomised controlled trial. *Lancet Infect Dis* 11: 102 – 109

Olotu A, Fegan G, Wambua J, Nyangweso G, Leach A, Lievens M, Kaslow DC, Njuguna P, Marsh K, Bejon P (2016) Seven-year efficacy of RTS,S/AS01 malaria vaccine among young African children. *N Engl J Med* 374: 2519 – 2529

Oyen D, Torres JL, Wille-Reece U, Ockenhouse CF, Emerling D, Glanville J, Volkmuth W, Flores-Garcia Y, Zavala F, Ward AB *et al* (2017) Structural basis for antibody recognition of the NANP repeats in *Plasmodium falciparum* circumsporozoite protein. *Proc Natl Acad Sci U S A* 114: E10438 – E10445

Pompon J, Levashina EA (2015) A new role of the mosquito complement-like Cascade in male fertility in *Anopheles gambiae*. *PLoS Biol* 13: e1002255

Sattabongkot J, Yimamnuaychoke N, Leelaudomlipi S, Rasameesoraj M, Jenwithisuk R, Coleman RE, Udomsangpetch R, Cui L, Brewer TG (2006) Establishment of a human hepatocyte line that supports *in vitro* development of the exo-erythrocytic stages of the malaria parasites *Plasmodium falciparum* and *P. vivax*. *Am J Trop Med Hyg* 74: 708 – 715

Scally SW, Murugan R, Bosch A, Triller G, Costa G, Mordmuller B, Kremsner PG, Sim BKL, Hoffman SL, Levashina EA *et al* (2018) Rare PfCSP C-terminal antibodies induced by live sporozoite vaccination are ineffective against malaria infection. *J Exp Med* 215: 63 – 75

Stoute JA, Slaoui M, Heppner DG, Momin P, Kester KE, Desmons P, Wellde BT, Garcon N, Krzych U, Marchand M (1997) A preliminary evaluation of a recombinant circumsporozoite protein vaccine against *Plasmodium falciparum* malaria. RTS,S Malaria Vaccine Evaluation Group. *N Engl J Med* 336: 86 – 91

Swearingen KE, Lindner SE, Shi L, Shears MJ, Harupa A, Hopp CS, Vaughan AM, Springer TA, Moritz RL, Kappe SH *et al* (2016) Interrogating the *Plasmodium* Sporozoite surface: identification of surface-exposed proteins and demonstration of glycosylation on CSP and TRAP by mass spectrometry-based proteomics. *PLoS Pathog* 12: e1005606

Tan J, Sack BK, Oyen D, Zenklusen I, Piccoli L, Barbieri S, Foglierini M, Fregni CS, Marcandalli J, Jongo S *et al* (2018) A public antibody lineage that potently inhibits malaria infection through dual binding to the circumsporozoite protein. *Nat Med* 24: 401 – 407

Thai E, Costa G, Weyrich A, Murugan R, Oyen D, Flores-Garcia Y, Prieto K, Bosch A, Valleriani A, Wu NC *et al* (2020) A high-affinity antibody against the CSP N-terminal domain lacks *Plasmodium falciparum* inhibitory activity. *J Exp Med* 217: e20200061

Triller G, Scally SW, Costa G, Pissarev M, Kreschel C, Bosch A, Marois E, Sack BK, Murugan R, Salman AM *et al* (2017) Natural parasite exposure induces protective human anti-malarial antibodies. *Immunity* 47: 1197 – 1209.e10

Wahl I, Obraztsova AS, Puchan J, Hundsdorfer R, Chakravarty S, Sim BKL, Hoffman SL, Kremsner PG, Mordmuller B, Wardemann H (2022) Clonal evolution and TCR specificity of the human TFH cell response to *Plasmodium falciparum* CSP. *Sci Immunol* 7: eabm9644

Wang Q, Fujioka H, Nussenzweig V (2005) Mutational analysis of the GPI-anchor addition sequence from the circumsporozoite protein of *Plasmodium*. *Cell Microbiol* 7: 1616 – 1626

Wang LT, Pereira LS, Flores-Garcia Y, O'Connor J, Flynn BJ, Schon A, Hurlburt NK, Dillon M, Yang ASP, Fabra-Garcia A *et al* (2020) A potent anti-malarial human monoclonal antibody targets circumsporozoite protein minor repeats and neutralizes sporozoites in the liver. *Immunity* 53: 733 – 744.e8

 © 2023 The Authors

Wang LT, Pereira LS, Kiyuka PK, Schon A, Kisalu NK, Vistein R, Dillon M, Bonilla BG, Molina-Cruz A, Barillas-Mury C *et al* (2021) Protective effects of combining monoclonal antibodies and vaccines against the *Plasmodium falciparum* circumsporozoite protein. *PLoS Pathog* 17: e1010133

Wardemann H, Busse CE (2019) Expression cloning of antibodies from single human B cells. *Methods Mol Biol* 1956: 105–125

Wardemann H, Yurasov S, Schaefer A, Young JW, Meffre E, Nussenzweig MC (2003) Predominant autoantibody production by early human B cell precursors. *Science* 301: 1374–1377

WHO (2021) *World malaria report 2021*, p 2021. Geneva: World Health Organization

Yaari G, Vander Heiden JA, Uduman M, Gadala-Maria D, Gupta N, Stern JN, O'Connor KC, Hafler DA, Laserson U, Vigneault F *et al* (2013) Models of somatic hypermutation targeting and substitution based on synonymous mutations from high-throughput immunoglobulin sequencing data. *Front Immunol* 4: 358

Zavala F, Cochrane AH, Nardin EH, Nussenzweig RS, Nussenzweig V (1983) Circumsporozoite proteins of malaria parasites contain a single immunodominant region with two or more identical epitopes. *J Exp Med* 157: 1947–1957

Zavala F, Tam JP, Hollingdale MR, Cochrane AH, Quakyi I, Nussenzweig RS, Nussenzweig V (1985) Rationale for development of a synthetic vaccine against *Plasmodium falciparum* malaria. *Science* 228: 1436–1440

License: This is an open access article under the terms of the Creative Commons Attribution License, which permits use, distribution and reproduction in any medium, provided the original work is properly cited.

Expanded View Figures

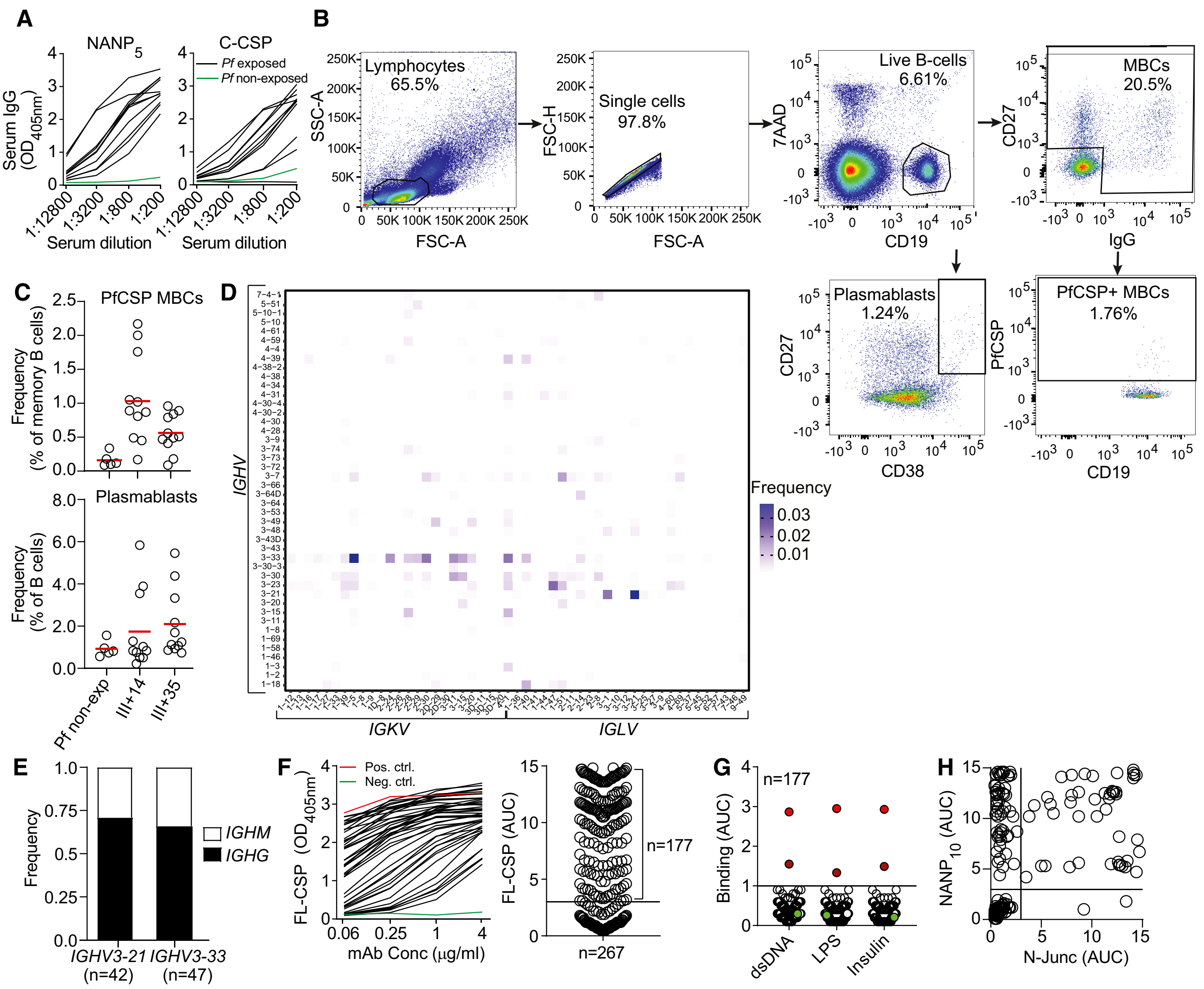

Figure EV1. Immunization with radiation-attenuated *Plasmodium falciparum* sporozoites (PfSPZ Vac) induces strong antibody responses against the PfCSP C terminus.

A Representative ELISA curves of serum IgG reactivity against PfCSP-derived $NANP_5$ peptide and C-CSP domain.

B Flow-cytometric single-cell isolation strategy for PfCSP-reactive memory B cells and plasmablasts from a representative immunized donor.

C Frequency of PfCSP-reactive memory B cells (upper panel) and plasmablasts (lower panel) in PBMCs of immunized donors at the indicated time points (III + 14 and III + 35) and non-immunized donors (Pf non-exp).

D Paired Ig heavy and light chain V gene usage in PfCSP-reactive memory B cells (n = 1,172).

E Isotype distribution of *IGHV3-21-* and *IGHV3-33*-encoded mAbs.

F Representative ELISA curves of mAbs from PfCSP-reactive memory B cells (black) or positive (red) and negative (green) control mAbs 2A10 (Triller *et al*, 2017) and mGO53 (Wardemann *et al*, 2003), respectively, binding to FL-CSP (left) with corresponding AUC values (right; n = 267).

G ELISA binding strength of FL-CSP-reactive mAbs to LPS, dsDNA or insulin compared to the highly polyreactive mAb ED38 (bright red; Meffre *et al*, 2004), weakly polyreactive mAb JB40 (dark red; Meffre *et al*, 2004), and the non-polyreactive mAb mGO53 (green; Wardemann *et al*, 2003).

H ELISA binding strength of FL-CSP-reactive mAbs to N-Junc peptide vs $NANP_{10}$.

Data information: Data in (C) represent one biological measurement obtained prior to cell sorting. Data in (F and H) indicate means from three independent technical replicates. Data in (G) are representative of two independent technical replicates.

© 2023 The Authors

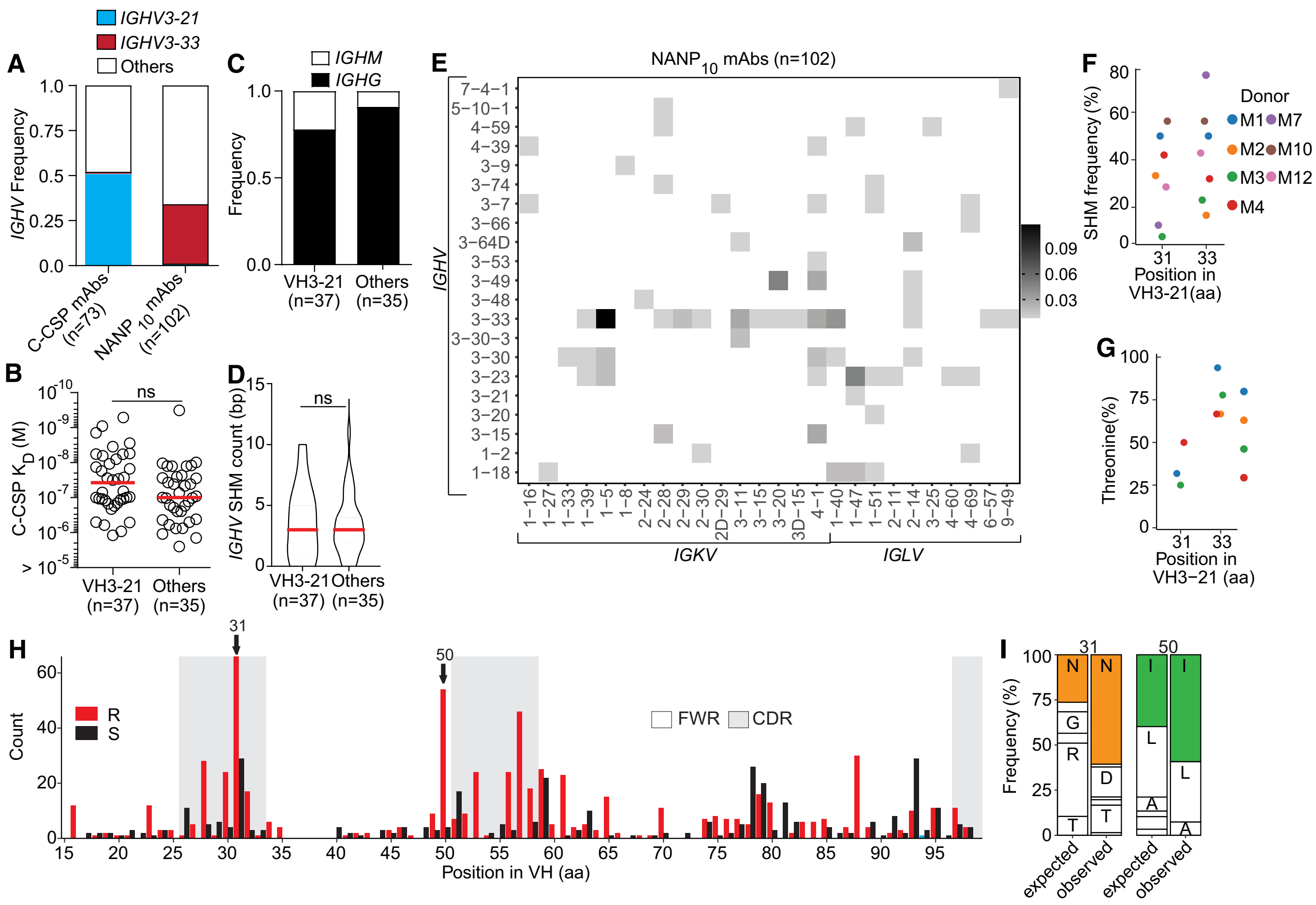

Figure EV2. Molecular characteristics and affinity of C-CSP specific mAbs.

A *IGHV3-21* or *IGHV3-33* gene frequency in C-CSP- and $NANP_{10}$-reactive mAbs.

B–D Comparison of C-CSP specific mAbs encoded by *IGHV3-21* and other *IGHV* genes. Anti-C-CSP SPR affinity (B), isotype distribution (C) and *IGHV* SHM count (D).

E Frequency of $NANP_{10}$-reactive mAbs encoded by the indicated *IGHV* + *IGKV* and *IGHV* + *IGLV* combinations (n = 102).

F, G Percentage of VH aa replacement SHM (F) and frequency of Thr at position H.31 and H.33 (G) in VH3-21 mAbs at donor level.

H Amino acid (aa) VH replacement (red bars) and silent (black bars) SHM in VH3-33 mAbs (n = 256). FWR, framework region; CDR, complementarity-determining region.

I Observed (Obs) aa usage frequency at position H.31 and H.50 in VH3-33 mAbs carrying a replacement mutation at these positions compared to the expected (Exp) neutral mutation model (Yaari *et al*, 2013; Gupta *et al*, 2015). Single-letters indicate aa residues: A, Ala; D, Asp; G, Gly; I, Ile; L, Leu; N, Asn; R, Arg; T, Thr.

Data information: Data in (F and G) represent donors with at least 5 VH3-21 mAbs and ≥ 4 mAbs with mutations at position H.33, respectively. Red lines in (B) and (D) indicate geometric and arithmetic mean values, respectively. ns, non-significant, two-tailed Mann–Whitney test (B, D). Data in (B) are representative of two independent technical replicates.

© 2023 The Authors

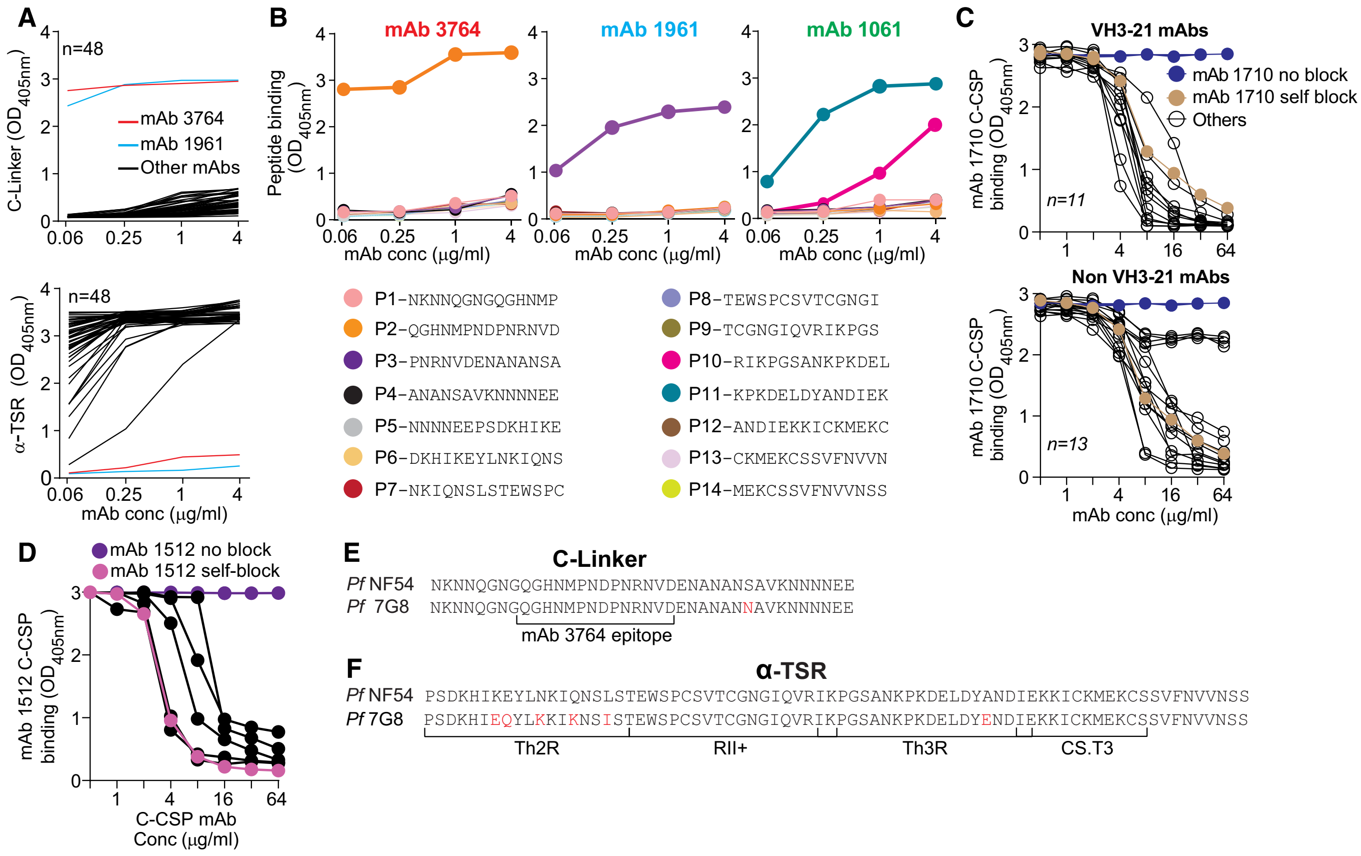

Figure EV3. C-CSP specific mAbs target preferentially two distinct conformational epitopes in the α-TSR domain.

A Representative ELISA binding curves for all C-CSP specific mAbs (black lines) to the C-linker (upper panel) and α-TSR (lower panel). The C-linker specific mAb 3764 (red line) and C-linker cross-reactive mAb 1961 (light blue line) are highlighted.

B Representative ELISA binding curves to overlapping peptides (P1-P14) covering the complete C-CSP for mAb 3764 (left), mAb 1961 (middle) and mAb 1061 (right). Amino acid sequences of the peptides are indicated.

C Representative ELISA curves illustrating the ability of individual VH3-21 (upper panel) and non-VH3-21 (lower panel) C-CSP specific mAbs (black lines) to block binding of mAb 1710 (Scally *et al*, 2018) to C-CSP. mAb 1710 C-CSP binding without blocking (blue) and after self-blocking (golden) is shown for comparison.

D ELISA curves showing C-CSP reactivity of mAb 1512 (Beutler *et al*, 2022) in a blocking ELISA with mAbs that do not block C-CSP binding of mAb 1710 (black). mAb 1512 binding without blocking (purple) and after self-blocking (magenta) is shown for comparison.

E, F Alignment of the PfCSP NF54 and 7G8 C-linker (E) and α-TSR (F) aa sequences. Amino acids that differ are highlighted in red.

 © 2023 The Authors

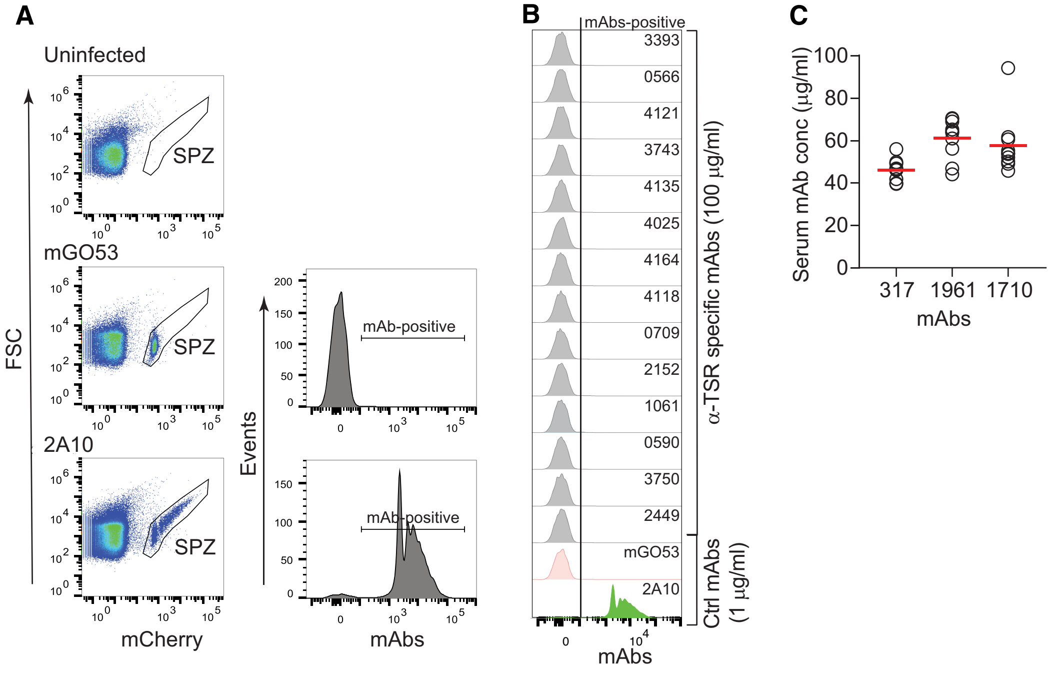

Figure EV4. Gating strategy to assess mAb binding to live PbPfCSP(mCherry) sporozoites.

A Flow cytometric gating strategy to analyze the proportion of mAb-positive PbPfCSP(mCherry) live sporozoites. Live mCherry-positive sporozoites (SPZ) were gated. Salivary gland extracts from uninfected mosquitoes were used as sporozoite gating control. The sporozoite binding profile of the negative control mAb mGO53 (Wardemann *et al*, 2003) and the positive control anti-NANP mAb 2A10 (Triller *et al*, 2017) are shown for comparison.

B Representative flow-cytometric profiles of live PbPfCSP(mCherry) sporozoites recognized by α-TSR-specific mAbs (100 μg/ml) as determined by flow cytometry. The positive control anti-NANP mAb 2A10 (1 μg/ml; Triller *et al*, 2017) and negative control non-PfCSP reactive mAb mGO53 (1 μg/ml; Wardemann *et al*, 2003) are shown for comparison.

C mAb serum concentrations in individual mice (n = 10 for mAb 317, n = 8 for mAb 1961, and n = 9 for mAb 1710) after passive transfer of the indicated monoclonal antibodies. The data in C only included mice that had detectable serum IgG concentrations of the indicated mAbs at the time of the challenge.

© 2023 The Authors

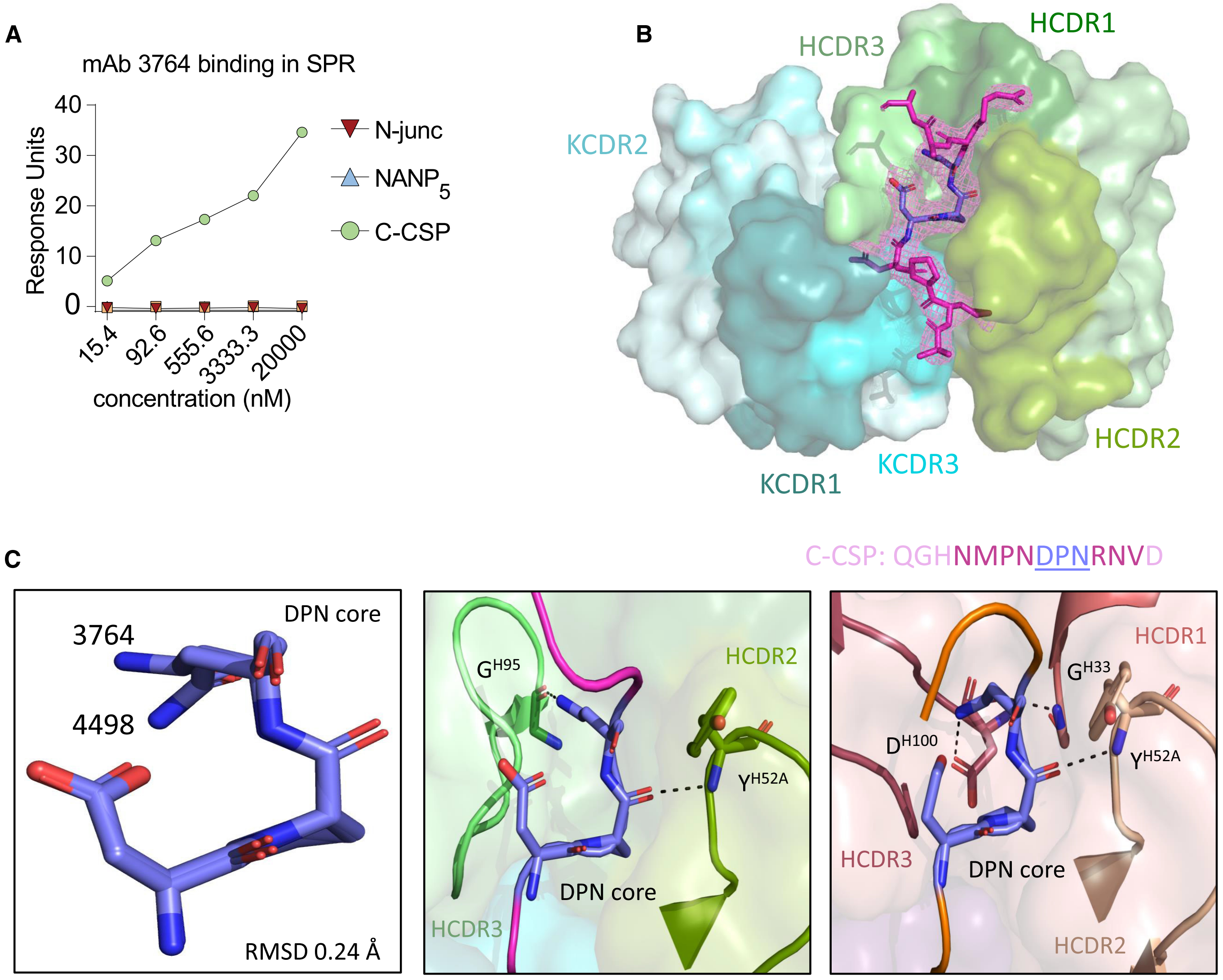

Figure EV5. Specificity of mAb 3764 towards the PfCSP C-terminal region.

A mAb 3764 SPR affinity measurement against peptides covering the N-term junction (N-Junc), $NANP_5$ or C-CSP at various concentrations.

B Composite omit map electron density contoured at 1 σ (light magenta mesh) around the C-CSP peptide (magenta).

C (Left) Superposition of the DPN core (deep blue) in crystal structures of mAb 3764 bound to the indicated C-CSP peptide (magenta) and mAb 4498 (Murugan *et al*, 2020) bound to the NDN3 peptide (orange) (PDB ID: 6ULF). (Middle) mAb 3764 HCDR2 and 3 (pea and light green, respectively) are shown interacting with the C-CSP peptide. (Right) mAb 4498 HCDR1, 2, and 3 (dark salmon, raspberry and wheat, respectively) are shown interacting with NDN3.

Data information: Data in (A) is a representative of two independent technical replicates.

 © 2023 The Authors

Molecular and functional properties of human *Plasmodium falciparum* CSP C-terminus antibodies

Opeyemi Ernest Oludada[1,2], Giulia Costa[3], Clare Burn Aschner[4], Anna S. Obraztsova[1,2], Katherine Prieto[4], Caterina Canetta[1], Stephen L. Hoffman[5], Peter G. Kremsner[6,7], Benjamin, Mordmüller[6,8], Rajagopal Murugan[1*], Jean-Philippe Julien[4,9*], Elena A. Levashina[3*], and Hedda Wardemann[1*]

[1]B Cell Immunology, German Cancer Research Center, Heidelberg, Germany

[2]Biosciences Faculty, University of Heidelberg, Germany

[3]Max Planck Institute for Infection Biology, Berlin, Germany

[4]The Hospital for Sick Children Research Institute, Toronto, Canada

[5]Sanaria Inc., Rockville, MD 20850, USA

[6]Institute of Tropical Medicine, Tübingen, Germany

[7]Centre de Recherches de Lambaréné (CERMEL), Lambaréné, Gabon

[8]Radboud University Medical Center, Nijmegen, The Netherlands

[9]Departments of Biochemistry and Immunology, University of Toronto, Toronto, Canada

*equal contribution

Correspondence: Hedda Wardemann (h.wardemann@dkfz.de), Elena A. Levashina (levashina@mpiib-berlin.mpg.de), Jean-Philippe Julien (jean-philippe.julien@sickkids.ca)

TABLE OF CONTENT

Appendix Table S1: Amino acid (aa) sequences of PfCSP antigens used in ELISAs

Appendix Table S2: Sequenced and cloned antibodies per time-point

Appendix Table S3: Epitope specificity and 7G8 cross-reactivity pattern of C-CSP specific mAbs

Appendix Table S4: Live sporozoites binding and hepatocyte traversal inhibition

Appendix Table S5: Statistical analysis for *in vivo* protection of 1961 mAb against mosquito bite challenge infection (Fig 4F).

Appendix Table S6: X-ray crystallography data collection and refinement data

APPENDIX TABLES

Appendix Table S1: Amino acid (aa) sequences of PfCSP antigens used in ELISAs

Antigen/Peptide	Sequence (aa)
Biotinylated FL-CSP (NF54)	MGILPSPGMPALLSLVSLLSVLLMGCVASLGENDDGNNEDN<br>EKLRKPKHKKLKQPADGNPDPNANPNVDPNANPNVDPNAN<br>PNVDPNANPNANPNANPNANPNANPNANPNANPNANPNA<br>NPNANPNANPNANPNANPNANPNANPNANPNANPNVDPN<br>ANPNANPNKNNQGNGQGHNMPNDPNRNVDENANANSAV<br>KNNNNEEPSDKHIKEYLNKIQNSLSTEWSPCSVTCGNGIQV<br>RIKPGSANKPKDELDYANDIEKKICKMEKCSSVFNVV**NSSG**<br>**SGLNDIFEAQKIEWHEGSHHHHHH**
7G8 FL-CSP	MGILPSPGMPALLSLVSLLSVLLMGCVASLGENDDGNNNN<br>GDNGREGKDEDKRDGNNEDNEKLRKPKHKKLKQPGDGNP<br>DPNANPNVDPNANPNVDPNANPNANPNANPNANPNANPN<br>ANPNANPNANPNANPNANPNANPNANPNANPNANPNANP<br>NVDPNANPNANPNANPNANPNKNNQGNGQGHNMPNDPN<br>RNVDENANANNAVKNNNNEEPSDKHIEQYLKKIKNSISTEW<br>SPCSVTCGNGIQVRIKPGSANKPKDELDYENDIEKKICKMEK<br>C**SSVFNVVNSSGSHHHHHH**
FL-CSP (NF54)	MEYQCYGSSSNTRVLNELNYDNAGTNLYNELEMNYYGKQE<br>NWYSLKKNSRSLGENDDGNNEDNEKLRKPKHKKLKQPAD<br>GNPDPNANPNVDPNANPNVDPNANPNVDPNANPNANPNA<br>NPNANPNANPNANPNANPNANPNANPNANPNANPNANPN<br>ANPNANPNANPNANPNANPNVDPNANPNANPNANPNANP<br>NANPNANPNANPNANPNANPNANPNANPNANPNANPNAN<br>PNANPNANPNANPNANPNKNNQGNGQGHNMPNDPNRNV<br>DENANANSAVKNNNNEEPSDKHIKEYLNKIQNSLSTEWSPC<br>SVTCGNGIQVRIKPGSANKPKDELDYANDIEKKICKMEKCSS<br>VFNVVNSSKLAAALEHHHHHH
N-Junc	GKQPADGNPDPNANPNVDPNANP
$NANP_{10}$	NANPNANPNANPNANPNANPNANPNANPNANPNANPNANP
C-CSP	NKNNQGNGQGHNMPNDPNRNVDENANANSAVKNNNNEE<br>PSDKHIKEYLNKIQNSLSTEWSPCSVTCGNGIQVRIKPGSAN<br>KPKDELDYANDIEKKICKMEKCSVFNVVNSS
C-linker	NKNNQGNGQGHNMPNDPNRNVDENANANSAVKNNNNEE
α-TSR	MGILPSPGMPALLSLVSLLSVLLMGCVAETGYVEFEPSDKHI<br>KEYLNKIQNSLSTEWSPCSVTCGNGIQVRIKPGSANKPKDE<br>LDYANDIEKKICKMEKCPHHHHHHALE
P1	NKNNQGNGQGHNMP
P2	QGHNMPNDPNRNVD
P3	PNRNVDENANANSA
P4	ANANSAVKNNNNEE
P5	NNNNEEPSDKHIKE
P6	DKHIKEYLNKIQNS
P7	NKIQNSLSTEWSPC
P8	TEWSPCSVTCGNGI
P9	TCGNGIQVRIKPGS

P10	RIKPGSANKPKDEL
P11	KPKDELDYANDIEK
P12	ANDIEKKICKMEKC
P13	CKMEKCSSVFNVVN
P14	MEKCSSVFNVVNSS

Appendix Table S2: Sequenced and cloned antibodies per time-point

	**PfCSP Memory B cells**				**Plasmablasts**	
	**Sequenced antibodies**		**Cloned antibodies**		**Sequenced antibodies**	
**Donor**	**III+14**	**III+35**	**III+14**	**III+35**	**III+14**	**III+35**
M1	67	107	24	23	67	NA
M2	25	35	4	13	53	NA
M3	41	36	13	12	216	196
M4	213	134	26	26	129	136
M5	43	NA	10	-	144	NA
M6	NA	37	-	12	NA	NA
M7	29	14	11	5	177	119
M8	NA	34	-	9	NA	86
M9	22	10	5	1	342	184
M10	24	73	9	15	140	NA
M11	NA	46	-	8	NA	NA
M12	132	50	29	12	147	244

Appendix Table S3: Epitope specificity and 7G8 cross-reactivity pattern of C-CSP specific mAbs

mAbs	Affinity	NF54 C-CSP binding (ELISA AUC) after blocking with the indicated mAbs		7G8 FL-CSP binding
ID	K_D (M)	mAb 1710	mAb 1512	ELISA AUC
3393	5.11E-10	15.48		1
1805	1.41E-09	21.54		1.3
4192	2.77E-09	18.35		0.8
3667	3.49E-09	22.46		1.4
3666	5.50E-09	50.69		2.9
0566	5.70E-09	18.1		0.6
2801	5.74E-09	42.07		0.6
4595	6.40E-09	19.76		10.1
1593	8.00E-09	19.72		0.5
2889	9.28E-09	18.09		0.6
4121	9.81E-09	36.09		12.7
0621	1.02E-08	40.09		1.6
3743	1.04E-08	22.59		0.7
3033	1.09E-08	17.49		13.4
3133	1.21E-08	18.4		0.6
1891	1.25E-08	48.59		0.9
3451	1.26E-08	69.77		0.8
0937	1.34E-08	28.11		1.4
1984	1.50E-08	26.99		1.3
3116	1.74E-08	23.48		0.9
4025	2.31E-08	145.8	28.33	13.2
4164	2.54E-08	25.04		0.5
3268	2.66E-08	41.77		1.8
4118	2.74E-08	149.3	45.37	10.8
2446	2.78E-08	46.61		1.5
2384	2.80E-08	45.21		0.8
3128	2.91E-08	26.48		2.4
4135	3.15E-08	47.63		2.2
2959	3.51E-08	143.9	75.75	13.1
0762	3.70E-08	41.61		0.8
2421	4.10E-08	69.39		1.4
0709	4.11E-08	144.4	27.04	13.1
3616	4.63E-08	49.29		0.6
2599	6.61E-08	31.44		1.1
0335	7.34E-08	51.57		0.5
3694	9.10E-08	155.2	68.04	13.5
3713	9.58E-08	55.03		0.6
3217	9.95E-08	42.57		12

mAb 1710 (Scally *et al.*, 2017)	7.01E-08	53.74	183.6	0.2
mAb 1512 (Beutler *et al.*, 2022)	2.85E-10	156.8	21.53	ND

mAbs- monoclonal antibodies; ID- identification number; AUC- area under the curve; ND- not determined

Appendix Table S4: Live sporozoites binding and hepatocyte traversal inhibition

		**mAbs**		**Binding affinity**	**Sporozoite binding (% average)**		**Traversal inhibition (% average)**
**ID**	**VH**	**VL**	**Epitope specificity**	**K_D (M)**	**100 µg/ml**	**1 µg/ml**	**100 µg/ml**
3393	3-21	Vλ3-1	Th2R/Th3R	5.11E-10	0.8		4.1
0566	3-21	Vλ3-21	Th2R/Th3R	5.70E-09	0.4		-3.2
4121	3-30	Vλ1-36	Th2R/Th3R	9.81E-09	0.7		0.7
3743	3-9	Vλ2-23	Th2R/Th3R	1.04E-08	0.6		9.9
4135	3-21	Vλ6-57	Th2R/Th3R	3.15E-08	0.6		-8.3
4025	4-61	Vκ3-20	RII+	2.31E-08	1		3.5
4164	3-7	Vλ3-1	Th2R/Th3R	2.54E-08	0.7		-1.6
4118	3-23	Vλ7-43	RII+	2.74E-08	0.5		5.1
0709	3-48	Vκ3-11	RII+	4.11E-08	0.6		-12.8
2152	3-23	Vκ1-13	ND	1.64E-07	1		2
3764	3-33	Vκ2-30	C-linker	1.72E-07	77	0.9	-12.8
1061	4-59	Vλ1-47	Linear peptide	1.93E-07	0.9		-10.7
0590	1-3	Vλ3-21	ND	2.45E-07	0.6		-13.7
3750	1-3	Vκ1-33	ND	4.17E-07	0.8		16.3
2449	3-9	Vκ1-12	ND	5.04E-07	0.7		3.1

mAbs- monoclonal antibodies; ID- identification number; VH- Variable Ig heavy chain segment; VL- Variable Ig light chain segment; ND- not determined

Appendix Table S5: Statistical analysis for *in vivo* protection of 1961 mAb against mosquito bite challenge infection (Fig 4F).

	**1961 (n=8)**	**317 (n=10)**
**1710 (n=9)**	0.0592	<0.0001
**1961 (n=8)**	-	0.027

P-values of pair comparisons are indicated, Mantel ‐ Cox log ‐ rank test, N=2.

Appendix Table S6: X-ray crystallography data collection and refinement data

	**3764 Fab - PfCSP$_{281-294}$**
Beamline	APS-23-ID-B
Wavelength (Å)	1.033167
Space group	$P2_1$
Cell dimensions	
a, b, c (Å)	92.2, 66.7, 95.5
α, β, γ (°)	90, 113.4, 90
Resolution (Å)[a]	40.0-2.36 (2.46-2.36)
No. molecules in ASU	2
No. observations	303,283 (34,515)
No. unique observations	43,719 (5067)
Multiplicity	6.9 (6.7)
R_{merge} (%)[b]	12.3 (63.4)
R_{pim} (%)[c]	5.0 (26.1)
$<I/\sigma I>$	10.9 (1.8)
$CC_{1/2}$	99.7 (74.9)
Completeness (%)	98.9 (98.6)
Refinement statistics	
Reflections used in refinement	43,695
Reflections used for R_{free}	1995
Non–hydrogen atoms	7007
Macromolecules	6718
Solvent	289
R_{work}[d]/R_{free}[e]	17.5 / 23.7
Rms deviations from ideality	
Bond lengths (Å)	0.007
Bond angle (°)	0.93
Ramachandran plot	
Favored regions (%)	97.6
Allowed regions (%)	2.3
Wilson B-value (Å^2)	43.7
B-factors (Å^2)	
Average	44.5
Macromolecules	44.5
Solvent	44.3

[a]Values in parentheses refer to the highest resolution bin.
[b]$R_{merge} = \Sigma hkl\ \Sigma i \mid Ihkl, i - <Ihkl> \mid / \Sigma hkl <Ihkl>$.
[c]$R_{pim} = \Sigma hkl\ [1/(N-1)]1/2\ \Sigma i \mid Ihkl, i - <Ihkl> \mid / \Sigma hkl <Ihkl>$.
[d]$R_{work} = (\Sigma \mid |Fo| - |Fc| \mid) / (\Sigma \mid |Fo|)$ for all data except as indicated in footnote [e].
[e]5% of data was used for the R_{free} calculation.
