# Supplementary material for: Molecular and functional properties of human Plasmodium falciparum CSP C‐terminus antibodies
Source: EMBO Mol Med. 2023 Apr 21;15(6):e17454. doi: 10.15252/emmm.202317454 (PMC10245032; doi:10.15252/emmm.202317454)
Supplement: Supplementary file 9 [file EMMM-15-e17454-s007.pdf]

EMBO Molecular Medicine

Molecular and functional properties of human Plasmodium falciparum CSP C-terminus antibodies

Opeyemi Oludada, Giulia Costa, Clare Burn Aschner, Anna Obraztsova, Katherine Prieto, Caterina Canetta, Stephen Hoffmann, Peter Kremsner, Benjamin Mordmueller, Rajagopal Murugan, Jean-Philippe Julien, Elena Levashina, and Hedda Wardemann
DOI: 10.15252/emmm.202317454

Corresponding authors: Hedda Wardemann (h.wardemann@dkfz-heidelberg.de) , Elena Levashina (levashina@mpiib-berlin.mpg.de), Jean-Philippe Julien (jean-philippe.julien@sickkids.ca)

Review Timeline:

Submission Date:	19th Jan 23
Editorial Decision:	13th Feb 23
Revision Received:	11th Mar 23
Accepted:	23rd Mar 23

Editor: Zeljko Durdevic

Transaction Report:

(Note: With the exception of the correction of typographical or spelling errors that could be a source of ambiguity, letters and reports are not edited. Depending on transfer agreements, referee reports obtained elsewhere may or may not be included in this compilation. Referee reports are anonymous unless the Referee chooses to sign their reports.)

13th Feb 2023

Dear Dr. Wardemann,

Thank you for the submission of your manuscript to EMBO Molecular Medicine. We have now received feedback from the three reviewers who agreed to evaluate your manuscript. As you will see from the reports below, all referees support publication of the study, but also raise important concerns that should be addressed in a revision of the current manuscript. No additional experiments are required. If you would like to discuss further the points raised by the referees, I am available to do so via email or video. Let me know if you are interested in this option.

Addressing the reviewers' concerns in full will be necessary for further considering the manuscript in our journal. EMBO Molecular Medicine encourages a single round of revision only and therefore, acceptance or rejection of the manuscript will depend on the completeness of your responses included in the next, final version of the manuscript. For this reason, and to save you from any frustrations in the end, I would strongly advise against returning an incomplete revision.

In addition, please amend the following:
1) Figures: Please upload separate, high-resolution main Figure files. Please check "Author Guidelines" for more information: https://www.embopress.org/page/journal/17574684/authorguide#figureformat
2) In the main manuscript file, please do the following:
- Correct/answer the track changes suggested by our data editors by working from the attached document.
- Remove supplementary figure legends and leave only main figure legends.
- In M&M, the statistical paragraph should reflect all information that you have filled in the Authors Checklist, especially regarding randomization, blinding, replication.
- In M&M, please include statement that the informed consent was obtained from the human subject and that in addition to the principles set out in the WMA Declaration of Helsinki the experiments also conformed the Department of Health and Human Services Belmont Report.
- Please rename "Competing Interest Statement" to "Disclosure Statement & Competing Interests". We updated our journal's competing interests policy in January 2022 and request authors to consider both actual and perceived competing interests. Please review the policy https://www.embopress.org/competing-interests and update your competing interests if necessary.
- Author contributions: Please remove it from the manuscript and specify author contributions in our submission system. CRediT has replaced the traditional author contributions section because it offers a systematic machine-readable author contributions format that allows for more effective research assessment. You are encouraged to use the free text boxes beneath each contributing author's name to add specific details on the author's contribution. More information is available in our guide to authors:
https://www.embopress.org/page/journal/17574684/authorguide#authorshipguidelines
- Add data availability statement that should contain information about data deposited in public repositories like PDB. Please be aware that all deposited datasets should be made freely available upon acceptance, without restriction. Use the following format to report the accession number of your data:

The datasets produced in this study are available in the following databases:
[data type]: [full name of the resource] [accession number/identifier] ([doi or URL or identifiers.org/DATABASE:ACCESSION])

Please check "Author Guidelines" for more information.
https://www.embopress.org/page/journal/17574684/authorguide#availabilityofpublishedmaterial
3) Appendix: Please rename the supplemental material file to Appendix and add table of content to the first page. Table S3 should be removed from the file, uploaded as an excel table and renamed to Dataset EV1, also in the main manuscript text. Please also rename figures and tables in the Appendix and in the main manuscript text to 'Appendix Figure S1' etc. and 'Appendix Table S1' etc.
4) The Paper Explained: Please provide "The Paper Explained" and add it to the main manuscript text. Please check "Author Guidelines" for more information. https://www.embopress.org/page/journal/17574684/authorguide#researcharticleguide
5) Synopsis: Every published paper now includes a 'Synopsis' to further enhance discoverability. Synopses are displayed on the journal webpage and are freely accessible to all readers. They include separate synopsis image and synopsis text.
- Synopsis image: Please provide the visual abstract as a high-resolution jpeg file 550 px-wide x (250-400)-px high.
- Synopsis text: Please provide a short standfirst (maximum of 300 characters, including space) as well as 2-5 one sentence bullet points that summarise the paper as a .doc file. Please write the bullet points to summarise the key NEW findings. They should be designed to be complementary to the abstract - i.e. not repeat the same text. We encourage inclusion of key acronyms and quantitative information (maximum of 30 words / bullet point). Please use the passive voice.
- Please check your synopsis text and image before submission with your revised manuscript. Please be aware that in the proof stage minor corrections only are allowed (e.g., typos).
6) For more information: This space should be used to list relevant web links for further consultation by our readers. Could you identify some relevant ones and provide such information as well? Some examples are patient associations, relevant databases, OMIM/proteins/genes links, author's websites, etc...

7) As part of the EMBO Publications transparent editorial process initiative (see our Editorial at http://embomolmed.embopress.org/content/2/9/329), EMBO Molecular Medicine will publish online a Review Process File (RPF) to accompany accepted manuscripts. This file will be published in conjunction with your paper and will include the anonymous referee reports, your point-by-point response and all pertinent correspondence relating to the manuscript. Let us know whether you agree with the publication of the RPF and as here, if you want to remove or not any figures from it prior to publication. Please note that the Authors checklist will be published at the end of the RPF.
8) Please provide a separate point-by-point response to my comments (as Word file).

We would welcome the submission of a revised version within three months for further consideration. Please let us know if you require longer to complete the revision.

I look forward to receiving your revised manuscript.

Yours sincerely,

Zeljko Durdevic

Zeljko Durdevic
Editor
EMBO Molecular Medicine

*** Instructions to submit your revised manuscript ***

*** PLEASE NOTE *** As part of the EMBO Publications transparent editorial process initiative (see our Editorial at https://www.embopress.org/doi/pdf/10.1002/emmm.201000094), EMBO Molecular Medicine will publish online a Review Process File to accompany accepted manuscripts.

In the event of acceptance, this file will be published in conjunction with your paper and will include the anonymous referee reports, your point-by-point response and all pertinent correspondence relating to the manuscript. If you do NOT want this file to be published, please inform the editorial office at contact@embomolmed.org.

When submitting your revised manuscript, please include:

1) a .docx formatted version of the manuscript text (including Figure legends and tables)

2) Separate figure files*

3) supplemental information as Expanded View and/or Appendix. Please carefully check the authors guidelines for formatting Expanded view and Appendix figures and tables at https://www.embopress.org/page/journal/17574684/authorguide#expandedview

4) a letter INCLUDING the reviewer's reports and your detailed responses to their comments (as Word file).

5) The paper explained: EMBO Molecular Medicine articles are accompanied by a summary of the articles to emphasize the major findings in the paper and their medical implications for the non-specialist reader. Please provide a draft summary of your article highlighting
- the medical issue you are addressing,
- the results obtained and
- their clinical impact.
This may be edited to ensure that readers understand the significance and context of the research.
Please refer to any of our published articles for an example.

6) For more information: There is space at the end of each article to list relevant web links for further consultation by our readers.

Could you identify some relevant ones and provide such information as well? Some examples are patient associations, relevant databases, OMIM/proteins/genes links, author's websites, etc...

7) Author contributions: the contribution of every author must be detailed in a separate section.

8) EMBO Molecular Medicine now requires a complete author checklist (https://www.embopress.org/page/journal/17574684/authorguide) to be submitted with all revised manuscripts. Please use the checklist as guideline for the sort of information we need WITHIN the manuscript. The checklist should only be filled with page numbers were the information can be found. This is particularly important for animal reporting, antibody dilutions (missing) and exact values and n that should be indicted instead of a range.

9) Every published paper now includes a 'Synopsis' to further enhance discoverability. Synopses are displayed on the journal webpage and are freely accessible to all readers. They include a short stand first (maximum of 300 characters, including space) as well as 2-5 one sentence bullet points that summarise the paper. Please write the bullet points to summarise the key NEW findings. They should be designed to be complementary to the abstract - i.e. not repeat the same text. We encourage inclusion of key acronyms and quantitative information (maximum of 30 words / bullet point). Please use the passive voice. Please attach these in a separate file or send them by email, we will incorporate them accordingly.

You are also welcome to suggest a striking image or visual abstract to illustrate your article. If you do please provide a jpeg file 550 px-wide x 400-px high.

10) A Conflict of Interest statement should be provided in the main text

11) Please note that we now mandate that all corresponding authors list an ORCID digital identifier. This takes <90 seconds to complete. We encourage all authors to supply an ORCID identifier, which will be linked to their name for unambiguous name identification.

Currently, our records indicate that the ORCID for your account is 0000-0003-3921-5933.

Please click the link below to modify this ORCID:
Link Not Available

12) The system will prompt you to fill in your funding and payment information. This will allow Wiley to send you a quote for the article processing charge (APC) in case of acceptance. This quote takes into account any reduction or fee waivers that you may be eligible for. Authors do not need to pay any fees before their manuscript is accepted and transferred to our publisher.

*Additional important information regarding Figures

Each figure should be given in a separate file and should have the following resolution:
Graphs 800-1,200 DPI
Photos 400-800 DPI
Colour (only CMYK) 300-400 DPI"

Figures are not edited by the production team. All lettering should be the same size and style; figure panels should be indicated by capital letters (A, B, C etc). Gridlines are not allowed except for log plots. Figures should be numbered in the order of their appearance in the text with Arabic numerals. Each Figure must have a separate legend and a caption is needed for each panel.

*Additional important information regarding figures and illustrations can be found at https://bit.ly/EMBOPressFigurePreparationGuideline. See also figure legend preparation guidelines: https://www.embopress.org/page/journal/17574684/authorguide#figureformat

The system will prompt you to fill in your funding and payment information. This will allow Wiley to send you a quote for the article processing charge (APC) in case of acceptance. This quote takes into account any reduction or fee waivers that you may be eligible for. Authors do not need to pay any fees before their manuscript is accepted and transferred to our publisher.

***** Reviewer's comments *****

Referee #1 (Comments on Novelty/Model System for Author):

This is an important and novel study, made particularly relevant and potentially impactful by the recent endorsement of the poorly protective RTS,S vaccine and the need to design new vaccines with enhanced efficacy. The study was made possible by access to samples collected from malaria-naïve individuals who underwent immunization with Sanaria's PfSPZ vaccine, consisting of radiation-attenuated Pf sporozoites, which constitutes a most appropriate model system to conduct this study.

Referee #1 (Remarks for Author):

The authors set out to identify and assess the inhibitory activity of monoclonal antibodies (mAbs) specific of the C-terminal region of the P. falciparum (Pf) circumsporozoite protein (CSP), and compare it with that conferred by mAbs targeting the central repeat region of this protein. This is an important study, made particularly relevant by the endorsement of the poorly protective RTS,S vaccine and the need to design new vaccines with enhanced efficacy. The study was made possible by access to samples collected from malaria-naïve individuals who underwent immunization with Sanaria's PfSPZ vaccine, consisting of radiation-attenuated Pf sporozoites. The authors show that immunization with PfSPZ elicits the production of 73 out of 177 antibodies that recognize the C-terminus of PfCSP. These are frequently encoded by the IGHV3-21 gene and preferentially target two conformational epitopes in the alpha-TSR domain of CSP, one of the two C-terminal subregions of this protein. However, when compared with mAb against the CSP repeat region, these C-terminal-specific mAbs showed low binding activity to Pf sporozoites and were poorly inhibitory of these parasites. In fact, only a single mAb reactive to the C-terminus of CSP, with cross-reactivity to the protein's repeat and N-terminal junction, was able to inhibit the parasite in vitro, but not in vivo.

The Introduction is thorough and well-structured, the figures are informative, and the conclusions are supported by the data. The Discussion is very well organized and very clear. Overall, I have no objections to the publication of the manuscript in its current form, except for one minor suggestion, regarding the last sentence of the Abstract and, indeed, the author's take home message summarized by that statement, which reads "The data provide novel insights in the human anti-C-linker and anti-α-TSR antibody response that support exclusion of the PfCSP C terminus from malaria vaccine designs". In my opinion, the statement that these data "support exclusion of the PfCSP C terminus from malaria vaccine designs" overlooks the fact that this region of CSP contains T-cell epitopes that may be key to an effective protection against infection and disease. As such, I urge the authors to revise this statement or at least to discuss the importance of the C-terminal region of CSP for T-cell immunity and protective efficacy. As it is, the statement completely overlooks this aspect of immunity and, in my opinion, is misleading.

Referee #2 (Comments on Novelty/Model System for Author):

Overall, this is a very high quality study, thorough in approach and clear in outcome. The use of the rodent model to assess Mab efficacy is essential (as human trials would be extremely challenging and likely unethical without prior demonstration in a mouse anyway). As such, I believe the right experiments have been undertaken.

Referee #2 (Remarks for Author):

This is an extremely thorough study, which has surveyed the less well-investigated C terminus of the lead vaccine target CSP from the Plasmodium malaria parasite. Surveying a suite of mabs isolated from human PfSPZ challenges, the authors systematically dissect the profile and target of numerous mabs, drilling down to the important (and not to be dismissed) ultimately negative result that Mabs targetting the C terminus and on the whole non-protective. This is important data and will help refine second and third generation vaccines (e.g. RTS,S/R21 2.0) or even entirely new vaccines that wish to target CSP but avoid immunogenic but likely non-protective domains of the protein. As such, I think the study is compelling and should be published. My comments are minor and perhaps more suggestion therefore rather than a demand for more laborious experimentation/change.

1. The non-accessibility of antibodies to the C terminus on the sporozoite surface, yet their generation in vivo is (to me) intriguing. Is there anything about the PfSPZ vaccine that might bias formation of C terminal antibodies that are otherwise inaccessible? E.g. might there be degraded CSP in the cocktail that elicits a non-protective response that wouldn't otherwise exist in live sporozoites (e.g. from a mosquito bite). Said another way, is there any evidence from direct bites that C-CSP targeting antibodies are less likely?

2. In the same vein, I wondered whether the authors had considered doing labelling experiments with Pf sporozoites, rather than Pb-PfCSP sporozoites - in case their is a difference in their heterologous presentation on Pb sporozoites, or indeed if any labelling had been trialled on PfSPZ (cryo-preserved) sporozoites? It might have been nice to see some images of these labelled to partner the flow data.

3. The final section on DPN binding is interesting but I admit to having found it hard to follow (e.g. use of BSA abbreviation - where elsewhere BSA means Bovine Serum Albumin). I just wondered whether this and Figure 5 could be simplified slightly just to make them easier for a non-structural biologist (but avid vaccinologist) to follow?

4. Figure 4 took me several reads to realise that Panel A had a separate panel - just wondered if this could be either divided into a new A/B or made distinct from Panel E below.

5. In Figure 4F mAb 1710 is listed as a test, and then in the same sentence listed as the control - this could be re-written for clarity.

Referee #3 (Comments on Novelty/Model System for Author):

This is really nice work and on the whole is done well. These are not the first human antibodies against CSP purified and tested, including others made against similar regions. The focus on the C-terminal domain and antibodies against this region is an interesting addition to the field and could help design better vaccines going forward. The finding of non functional (non protective) antibodies at this region is unfortunate, but this information should be used to build a better vaccine construct.

Referee #3 (Remarks for Author):

Oludada et al describe purification and characterisation of a series of human derived monoclonal antibodies that target the C-terminus of the malaria sporozoite circumsporozoite protein (C-CSP). The manuscript is well written and the data nicely presented, I quite enjoyed reading about this study. The manuscript establishes a series of antibodies with similar origins, specificity and functional properties and shows that antibodies to this region of CSP have limited efficacy in blocking sporozoite host-cell entry. Although the inability to find a blocking antibody targeting this domain is unfortunate for the overall impact of the study, the fact that this paper helps to rule out much of the c-terminal domain as a target is of benefit for future vaccine design. The authors have done a very nice job of putting this paper together and I found little to criticise.

Major comments
-Discussion: The authors state that.

In response to RTS,S/AS01 immunization, anti-NANP and anti-C-terminus antibody titers correlate with protection (Chaudhury et al, 2021; Chaudhury et al, 2016; Dobano et al, 16 2019). To what degree the C-CSP-reactive serum antibodies recognize C-CSP specifically or cross-react with the repeat and junction has not been determined. Therefore, it is unclear whether protection is associated with C-CSP-specific or cross-reactive antibodies.

The summary suggests here that high titres of vaccine induced C-CSP antibodies must have some role in protection, perhaps through targeting domains other than some of the ones focussed on here. However, could not the antibody titres received during vaccination be simply a marker of antibody production (i.e. antibodies are made against the region in high titres because it is part of the vaccine), with the anti-NANP antibodies doing the bulk of the protective work and the C-CSP antibodies themselves do little functional protection? The data presented in this study demonstrate convincingly that a lot of antibodies to the C-CSP epitope have limited/no functional activity. While its possible that cell-mediated responses may confer protection via C-CSP targeting antibodies, this goes against the theory postulated here that antibodies targeting this domain don't work because they can't access it. In effect, the anti-C-CSP antibodies titres might be significant only as markers of exposure and antibody production towards the vaccine and have no role in protection. Is this possible and worth considering in the discussion?

RESPONSE TO REVIEWERS:

Referee #1 (Comments on Novelty/Model System for Author):
This is an important and novel study, made particularly relevant and potentially impactful by the recent endorsement of the poorly protective RTS,S vaccine and the need to design new vaccines with enhanced efficacy. The study was made possible by access to samples collected from malaria-naïve individuals who underwent immunization with Sanaria's PfSPZ vaccine, consisting of radiation-attenuated Pf sporozoites, which constitutes a most appropriate model system to conduct this study.

We thank the reviewer for the positive feedback.

Referee #1 (Remarks for Author):
The authors set out to identify and assess the inhibitory activity of monoclonal antibodies (mAbs) specific of the C-terminal region of the P. falciparum (Pf) circumsporozoite protein (CSP), and compare it with that conferred by mAbs targeting the central repeat region of this protein. This is an important study, made particularly relevant by the endorsement of the poorly protective RTS,S vaccine and the need to design new vaccines with enhanced efficacy. The study was made possible by access to samples collected from malaria-naïve individuals who underwent immunization with Sanaria's PfSPZ vaccine, consisting of radiation-attenuated Pf sporozoites. The authors show that immunization with PfSPZ elicits the production of 73 out of 177 antibodies that recognize the C-terminus of PfCSP. These are frequently encoded by the IGHV3-21 gene and preferentially target two conformational epitopes in the alpha-TSR domain of CSP, one of the two C-terminal subregions of this protein. However, when compared with mAb against the CSP repeat region, these C-terminal-specific mAbs showed low binding activity to Pf sporozoites and were poorly inhibitory of these parasites. In fact, only a single mAb reactive to the C-terminus of CSP, with cross-reactivity to the protein's repeat and N-terminal junction, was able to inhibit the parasite in vitro, but not in vivo.
The Introduction is thorough and well-structured, the figures are informative, and the conclusions are supported by the data. The Discussion is very well organized and very clear. Overall, I have no objections to the publication of the manuscript in its current form, except for one minor suggestion, regarding the last sentence of the Abstract and, indeed, the author's take home message summarized by that statement, which reads "The data provide novel insights in the human anti-C-linker and anti-α-TSR antibody response that support exclusion of the PfCSP C terminus from malaria vaccine designs". In my opinion, the statement that these data "support exclusion of the PfCSP C terminus from malaria vaccine designs" overlooks the fact that this region of CSP contains T-cell epitopes that may be key to an effective protection against infection and disease. As such, I urge the authors to revise this statement or at least to discuss the importance of the C-terminal region of CSP for T-cell immunity and protective efficacy. As it is, the statement completely overlooks this aspect of immunity and, in my opinion, is misleading.

We would like to thank the reviewer for raising this important point, which we now address as follows in the discussion:
“Future studies will need to determine whether efforts to design a second generation PfCSP vaccine might benefit from suppressing or even abrogating the humoral response against C-CSP, e.g. by boosting the anti-repeat and junction response. However, exclusion of the complete domain, especially of the highly immunodominant α-TSR, would eliminate the main T helper cell epitopes with likely strong negative effects on the quality and strength of the humoral response against the potent repeat and junction epitopes. Inclusion of linear peptide epitopes rather than the complete C-CSP may be sufficient to provide efficient T cell help without inducing non-protective humoral responses (Wahl et al., 2022). Alternatively, non-PfCSP T cell epitopes could substitute for the loss of T cell help and promote affinity maturation of the PfCSP-specific response. “

Referee #2 (Comments on Novelty/Model System for Author):
Overall, this is a very high-quality study, thorough in approach and clear in outcome. The use of the rodent model to assess Mab efficacy is essential (as human trials would be extremely challenging and likely unethical without prior demonstration in a mouse anyway). As such, I believe the right experiments have been undertaken.

We thank the reviewer for the positive feedback.

Referee #2 (Remarks for Author):
This is an extremely thorough study, which has surveyed the less well-investigated C terminus of the lead vaccine target CSP from the Plasmodium malaria parasite. Surveying a suite of mabs isolated from human PfSPZ challenges, the authors systematically dissect the profile and target of numerous mabs, drilling down to the important (and not to be dismissed) ultimately negative result that Mabs targetting the C terminus and on the whole non-protective. This is important data and will help refine second and third generation vaccines (e.g. RTS,S/R21 2.0) or even entirely new vaccines that wish to target CSP but avoid immunogenic but likely non-protective domains of the protein. As such, I think the study is compelling and should be published. My comments are minor and perhaps more suggestion therefore rather than a demand for more laborious experimentation/change.

1. The non-accessibility of antibodies to the C terminus on the sporozoite surface, yet their generation in vivo is (to me) intriguing. Is there anything about the PfSPZ vaccine that might bias formation of C terminal antibodies that are otherwise inaccessible? E.g. might there be degraded CSP in the cocktail that elicits a non-protective response that wouldn't otherwise exist in live sporozoites (e.g. from a mosquito bite). Said another way, is there any evidence from direct bites that C-CSP targeting antibodies are less likely?

The reviewer raises a very interesting point. To the best of our knowledge, it is unclear whether the PfSPZ antigens are presented differently to the immune system than the antigens of mosquito-transmitted sporozoites. The overall weak response induced by natural parasites and strong differences in the number of transmitted parasites in the field vs. PfSPZ vaccination and different routes of parasite injection (s.c. for mosquito-bites vs. i.v. for PfSPZ vaccination) make direct quantitative comparisons difficult.

2. In the same vein, I wondered whether the authors had considered doing labelling experiments with Pf sporozoites, rather than Pb-PfCSP sporozoites - in case their is a difference in their heterologous presentation on Pb sporozoites, or indeed if any labelling had been trialled on PfSPZ (cryo-preserved) sporozoites? It might have been nice to see some images of these labelled to partner the flow data.

We used transgenic Pb-PfCSP parasites for quantification of antibody binding capacity to live sporozoites because they express mCherry, a red fluorescence marker that allows us to accurately gate single sporozoites for flow cytometry analyses. We have used live Pf and Pb-PfCSP sporozoites in IFA analyses and did not detect differences in the staining pattern, however, this method has low resolution for detection of fine differences related to heterologous expression. The reviewer raised an interesting question about potential changes in antibody binding to irradiated or cryo-preserved parasites. However, to the best of our knowledge, this question has not yet been quantitatively assessed, likely due to the lack of a *Pf* fluorescence reporter line compatible with *A. gambiae* mosquitoes.

3. The final section on DPN binding is interesting but I admit to having found it hard to follow (e.g. use of BSA abbreviation - where elsewhere BSA means Bovine Serum Albumin). I just

wondered whether this and Figure 5 could be simplified slightly just to make them easier for a non-structural biologist (but avid vaccinologist) to follow?

We appreciate the complexity of the structural data, and have included some additional context with the results. No changes have been made to the figures, however, additional guidance on interpreting the figures has been provided in the results, and the important take-home messages from the structural data are emphasized in the discussion.

4. Figure 4 took me several reads to realise that Panel A had a separate panel - just wondered if this could be either divided into a new A/B or made distinct from Panel E below.

We agree with the reviewer that Panel A was difficult to read and have modified Fig. 4 accordingly. For clarity, we now show the data for each antibody separately and indicate the respective affinity in Panel A. The raw data panel has been moved to extended view figure 4.

5. In Figure 4F mAb 1710 is listed as a test, and then in the same sentence listed as the control - this could be re-written for clarity.

For clarity, we modified the legend as follows:
"Capacity of the passively-transferred indicated antibodies (100 µg) to protect mice (n=10 for mAb 317 (Oyen et al., 2017), n=8 for mAb 1961, and n=9 for mAb 1710 (Scally et al., 2018)) from parasitemia after the bite of three PbPfCSP(mCherry)-infected mosquitoes."

Referee #3 (Comments on Novelty/Model System for Author):
This is really nice work and on the whole is done well. These are not the first human antibodies against CSP purified and tested, including others made against similar regions. The focus on the C-terminal domain and antibodies against this region is an interesting addition to the field and could help design better vaccines going forward. The finding of non functional (non protective) antibodies at this region is unfortunate, but this information should be used to build a better vaccine construct.

Referee #3 (Remarks for Author):
Oludada et al describe purification and characterisation of a series of human derived monoclonal antibodies that target the C-terminus of the malaria sporozoite circumsporozoite protein (C-CSP). The manuscript is well written and the data nicely presented, I quite enjoyed reading about this study. The manuscript establishes a series of antibodies with similar origins, specificity and functional properties and shows that antibodies to this region of CSP have limited efficacy in blocking sporozoite host-cell entry. Although the inability to find a blocking antibody targeting this domain is unfortunate for the overall impact of the study, the fact that this paper helps to rule out much of the c-terminal domain as a target is of benefit for future vaccine design. The authors have done a very nice job of putting this paper together and I found little to criticise.

We thank the reviewer for the positive feedback.

Major comments
-Discussion: The authors state that.
In response to RTS,S/AS01 immunization, anti-NANP and anti-C-terminus antibody titers correlate with protection (Chaudhury et al, 2021; Chaudhury et al, 2016; Dobano et al, 16 2019). To what degree the C-CSP-reactive serum antibodies recognize C-CSP specifically or cross-react with the repeat and junction has not been determined. Therefore, it is unclear whether protection is associated with C-CSP-specific or cross-reactive antibodies. The summary suggests here that high titres of vaccine induced C-CSP antibodies must have some role in protection, perhaps through targeting domains other than some of the ones focussed on here. However, could not the antibody titres received during vaccination be

simply a marker of antibody production (i.e. antibodies are made against the region in high titres because it is part of the vaccine), with the anti-NANP antibodies doing the bulk of the protective work and the C-CSP antibodies themselves do little functional protection? The data presented in this study demonstrate convincingly that a lot of antibodies to the C-CSP epitope have limited/no functional activity. While its possible that cell-mediated responses may confer protection via C-CSP targeting antibodies, this goes against the theory postulated here that antibodies targeting this domain don't work because they can't access it. In effect, the anti-C-CSP antibodies titres might be significant only as markers of exposure and antibody production towards the vaccine and have no role in protection. Is this possible and worth considering in the discussion?

The reviewer raises a very valid point. We would like to clarify that we fully agree that most of the protective capacity comes from anti-repeat antibodies and that C-CSP specific antibodies alone would not show any parasite inhibitory effect. Indeed, the C-CSP antibodies might simply be a marker of exposure and of the overall strength of the anti-parasite response. To clarify this point, we have modified the discussion as follows:
"In response to RTS,S/AS01 immunization, anti-NANP and anti-C-terminus antibody titers correlate with protection (Chaudhury et al, 2021; Chaudhury et al, 2016; Dobano et al, 2019). To what degree the C-CSP-reactive serum antibodies recognize C-CSP specifically or cross-react with the repeat and junction has not been determined. Therefore, it is unclear whether protection is associated with C-CSP-specific or cross-reactive antibodies or whether C-CSP antibodies are simply a marker of the overall strength of the anti-parasite response whereas protection is mediated by anti-repeat antibodies. "

23rd Mar 2023

Dear Dr. Wardemann,

We are pleased to inform you that your manuscript is accepted for publication and is now being sent to our publisher to be included in the next available issue of EMBO Molecular Medicine.

Please read below for additional IMPORTANT information regarding your article, its publication and the production process.

Congratulations on your interesting work,

Zeljko Durdevic

Zeljko Durdevic
Editor
EMBO Molecular Medicine

Follow us on Twitter @EmboMolMed
Sign up for eTOCs at embopress.org/alertsfeeds

*** *** *** IMPORTANT INFORMATION *** *** ***

SPEED OF PUBLICATION
The journal aims for rapid publication of papers, using using the advance online publication "Early View" to expedite the process: A properly copy-edited and formatted version will be published as "Early View" after the proofs have been corrected. Please help the Editors and publisher avoid delays by providing e-mail address(es), telephone and fax numbers at which author(s) can be contacted.

Should you be planning a Press Release on your article, please get in contact with embomolmed@wiley.com as early as possible, in order to coordinate publication and release dates.

LICENSE AND PAYMENT:

All articles published in EMBO Molecular Medicine are fully open access: immediately and freely available to read, download and share.

EMBO Molecular Medicine charges an article processing charge (APC) to cover the publication costs. You, as the corresponding author for this manuscript, should have already received a quote with the article processing fee separately. Please let us know in case this quote has not been received.

Once your article is at Wiley for editorial production you will receive an email from Wiley's Author Services system, which will ask you to log in and will present you with the publication license form for completion. Within the same system the publication fee can be paid by credit card, an invoice, pro forma invoice or purchase order can be requested.

Payment of the publication charge and the signed Open Access Agreement form must be received before the article can be published online.

PROOFS

You will receive the proofs by e-mail approximately 2 weeks after all relevant files have been sent o our Production Office. Please return them within 48 hours and if there should be any problems, please contact the production office at embopressproduction@wiley.com.

Please inform us if there is likely to be any difficulty in reaching you at the above address at that time. Failure to meet our

deadlines may result in a delay of publication.

All further communications concerning your paper proofs should quote reference number EMM-2023-17454-V2 and be directed to the production office at embopressproduction@wiley.com.

Thank you,

Zeljko Durdevic
Editor
EMBO Molecular Medicine

EMBO Press Author Checklist

Corresponding Author Name: Hedda Wardemann	
Journal Submitted to: EMBO Molecular Medicine	
Manuscript Number: EMM-2023-17454	

USEFUL LINKS FOR COMPLETING THIS FORM
[The EMBO Journal - Author Guidelines](#)
[EMBO Reports - Author Guidelines](#)
[Molecular Systems Biology - Author Guidelines](#)
[EMBO Molecular Medicine - Author Guidelines](#)

Reporting Checklist for Life Science Articles (updated January 2022)
This checklist is adapted from Materials Design Analysis Reporting (MDAR) Checklist for Authors. MDAR establishes a minimum set of requirements in transparent reporting in the life sciences (see Statement of Task: 10.31222/osf.io/9sm4x). Please follow the journal's guidelines in preparing your manuscript.
Please note that a copy of this checklist will be published alongside your article.

Abridged guidelines for figures
1. Data
The data shown in figures should satisfy the following conditions:
- the data were obtained and processed according to the field's best practice and are presented to reflect the results of the experiments in an accurate and unbiased manner.
- ideally, figure panels should include only measurements that are directly comparable to each other and obtained with the same assay.
- plots include clearly labeled error bars for independent experiments and sample sizes. Unless justified, error bars should not be shown for technical replicates.
- if n<5, the individual data points from each experiment should be plotted. Any statistical test employed should be justified.
- Source Data should be included to report the data underlying figures according to the guidelines set out in the authorship guidelines on Data Presentation.

2. Captions
Each figure caption should contain the following information, for each panel where they are relevant:
- a specification of the experimental system investigated (eg cell line, species name).
- the assay(s) and method(s) used to carry out the reported observations and measurements.
- an explicit mention of the biological and chemical entity(ies) that are being measured.
- an explicit mention of the biological and chemical entity(ies) that are altered/varied/perturbed in a controlled manner.
- the exact sample size (n) for each experimental group/condition, given as a number, not a range;
- a description of the sample collection allowing the reader to understand whether the samples represent technical or biological replicates (including how many animals, litters, cultures, etc.).
- a statement of how many times the experiment shown was independently replicated in the laboratory.
- definitions of statistical methods and measures:
  - common tests, such as t-test (please specify whether paired vs. unpaired), simple χ2 tests, Wilcoxon and Mann-Whitney tests, can be unambiguously identified by name only, but more complex techniques should be described in the methods section;
  - are tests one-sided or two-sided?
  - are there adjustments for multiple comparisons?
  - exact statistical test results, e.g., P values = x but not P values < x;
  - definition of 'center values' as median or average;
  - definition of error bars as s.d. or s.e.m.

Please complete ALL of the questions below.
Select "Not Applicable" only when the requested information is not relevant for your study.

Materials

Newly Created Materials	Information included in the manuscript?	In which section is the information available? (Reagents and Tools Table, Materials and Methods, Figures, Data Availability Section)
New materials and reagents need to be available; do any restrictions apply?	Not Applicable	

Antibodies	Information included in the manuscript?	In which section is the information available? (Reagents and Tools Table, Materials and Methods, Figures, Data Availability Section)
For **antibodies** provide the following information:<br>- Commercial antibodies: RRID (if possible) or supplier name, catalogue number and or/clone number<br>- Non-commercial: RRID or citation	Yes	Materials, Methods, results and figures Section:<br>Commercially available antibodies used are: mouse anti-human CD27-PE (Clone ID: M-T271; Cat. No: 555441), CD38-BV605 (Clone ID: HB7; Cat. No: 562665), IgG-BV510 (Clone ID: G18-145; Cat. No: 563247), IgM-BV421 (Clone ID: G20-127; Cat. No: 562618), IgD- APC-H7 (Clone ID: IA6-2; Cat.

DNA and RNA sequences	Information included in the manuscript?	In which section is the information available? (Reagents and Tools Table, Materials and Methods, Figures, Data Availability Section)
**Short novel DNA or RNA including primers, probes**: provide the sequences.	Not Applicable	

Cell materials	Information included in the manuscript?	In which section is the information available? (Reagents and Tools Table, Materials and Methods, Figures, Data Availability Section)
**Cell lines:** Provide species information, strain. Provide accession number in repository **OR** supplier name, catalog number, clone number, and/**OR** RRID.	Yes	Materials and Methods Section: FreeStyle™ 293-F Cells (Thermo Fischer Scientific, Cat#R79007); HC-04, BEI Resources (MRA-975).
**Primary cultures:** Provide species, strain, sex of origin, genetic modification status.	Not Applicable	
Report if the cell lines were recently **authenticated** (e.g., by STR profiling) and tested for mycoplasma contamination.	Yes	Materials and Methods Section: All cell lines were tested negative for *Mycoplasma* contamination.

Experimental animals	Information included in the manuscript?	In which section is the information available? (Reagents and Tools Table, Materials and Methods, Figures, Data Availability Section)
**Laboratory animals or Model organisms:** Provide species, strain, sex, age, genetic modification status. Provide accession number in repository **OR** supplier name, catalog number, clone number, **OR** RRID.	Yes	Materials and Methods Section: Female C57BL/6J mice (8 weeks old), female CD-1 mice (7-12 weeks old), *Anopheles coluzzii* (Ngousso strain) (Harris et al. 2010), transgenic *Anopheles gambiae* (7b strain) (Pompon & Levashina, 2015)
**Animal observed in or captured from the field:** Provide species, sex, and age where possible.	Not Applicable	
Please detail **housing and husbandry conditions**.	Yes	Materials and Methods Section: Animals were bred in the MPIIB Experimental Animal Facility (Marienfelde, Berlin) and housed in a pathogen-free animal

Plants and microbes	Information included in the manuscript?	In which section is the information available? (Reagents and Tools Table, Materials and Methods, Figures, Data Availability Section)
**Plants:** provide species and strain, ecotype and cultivar where relevant, unique accession number if available, and source (including location for collected wild specimens).	Not Applicable	
**Microbes:** provide species and strain, unique accession number if available, and source.	Yes	Materials and Methods Section: Bacteria. MAX efficiency DH10B™ (ThermoFisher scientific; Cat. No: 18297010) competent cell was used for DNA transformation. Parasites: trangenic *Plasmodium berghei* ANKA strain

Human research participants	Information included in the manuscript?	In which section is the information available? (Reagents and Tools Table, Materials and Methods, Figures, Data Availability Section)
If collected and within the bounds of privacy constraints report on age, sex and gender or ethnicity for all study participants.	Not Applicable	These demographics are listed in recent publication: Mordmüller et al. A PfSPZ vaccine immunization regimen equally protective against homologous and heterologous controlled human malaria infection. NPJ Vaccines. 2022

Core facilities	Information included in the manuscript?	In which section is the information available? (Reagents and Tools Table, Materials and Methods, Figures, Data Availability Section)
If your work benefited from core facilities, was their service mentioned in the acknowledgments section?	Yes	Acknowledgement: This work benefited from core facility support.

Design

Study protocol	Information included in the manuscript?	In which section is the information available? (Reagents and Tools Table, Materials and Methods, Figures, Data Availability Section)

If study protocol has been **pre-registered, provide DOI in the manuscript**. For clinical trials, provide the trial registration number **OR** cite DOI.	Not Applicable	The clinical trial is described in recent publication: Mordmüller et al. A PfSPZ vaccine immunization regimen equally protective against homologous and heterologous controlled human malaria infection. NPJ Vaccines. 2022 doi: 10.1038/s41541-022-00510-z. The manuscript is cited in the respective
Report the **clinical trial registration number** (at ClinicalTrials.gov or equivalent), where applicable.	Not Applicable	The samples used in this study were collected from the clinical trial registered with ClinicalTrials.gov, NCT02704533. The clinical trial registration number is also mentioned in recent publication: Mordmüller et al. A PfSPZ vaccine

Laboratory protocol	Information included in the manuscript?	In which section is the information available?<br>(Reagents and Tools Table, Materials and Methods, Figures, Data Availability Section)
Provide DOI OR other citation details if **external detailed step-by-step protocols** are available.	Yes	Detailed protocols for cloning and expression of monoclonal antibodies can be found in our recent publication: Wardemann H, Busse CE. Expression Cloning of Antibodies from Single Human B Cells. *Methods Mol Biol*.

Experimental study design and statistics	Information included in the manuscript?	In which section is the information available?<br>(Reagents and Tools Table, Materials and Methods, Figures, Data Availability Section)
Include a statement about **sample size** estimate even if no statistical methods were used.	Not Applicable	
Were any steps taken to minimize the effects of subjective bias when allocating animals/samples to treatment (e.g. **randomization procedure**)? If yes, have they been described?	Yes	Materials and Methods Section: Mice were randomly assigned to their respective experimental groups from the same batches.
Include a statement about **blinding** even if no blinding was done.	Yes	Materials and Methods Section: Investigators were not blinded to group assignment during the experiment and/or when evaluating the outcome.
Describe **inclusion/exclusion criteria** if samples or animals were excluded from the analysis. Were the criteria pre-established?<br><br>If sample or data points were omitted from analysis, report if this was due to attrition or intentional exclusion and provide justification.	Yes	Figure legends: The pre-established inclusion criteria for mice in challenge experiment was the presence of detectable levels of mAbs at the time of the challenge. Two mice were excluded from the mAb 1961 immunized group and one mouse was excluded from the mAb 1710 immunized group because their mAb serum concentrations were not detectable. No samples were excluded [illegible]
For every figure, are **statistical tests** justified as appropriate? Do the data meet the assumptions of the tests (e.g., normal distribution)? Describe any methods used to assess it. Is there an estimate of variation within each group of data? Is the variance similar between the groups that are being statistically compared?	Yes	Materials and Methods Section: Statistical analysis was done with GraphPad Prism (version 9.12) using the two-tailed Mann-Whitney assuming a non-normally distributed data and the two-tailed Mantel-Cox log-rank test for the *in vivo* experiments.

Sample definition and in-laboratory replication	Information included in the manuscript?	In which section is the information available?<br>(Reagents and Tools Table, Materials and Methods, Figures, Data Availability Section)
In the figure legends: state number of times the experiment was **replicated** in laboratory.	Yes	All experimental results were reproduced at least twice and the number of replicates are indicated in the corresponding figure legends.
In the figure legends: define whether data describe **technical or biological replicates**.	Yes	All technical or biological replicates are indicated in the figure legends

Ethics

Ethics	Information included in the manuscript?	In which section is the information available?<br>(Reagents and Tools Table, Materials and Methods, Figures, Data Availability Section)
Studies involving **human participants**: State details of **authority granting ethics approval** (IRB or equivalent committee(s), provide reference number for approval.	Not Applicable	Human participants were not directly involved in thus study. However, details of authority granting ethics are described in a recent publication: Mordmüller et al. A PfSPZ vaccine immunization regimen equally protective against homologous and heterologous controlled human malaria infection. NPJ Vaccines. 2022 doi: 10.1038/s41541-022-00510-z. The manuscript is cited in the respective results and methods sections.
Studies involving **human participants**: Include a statement confirming that **informed consent** was obtained from all subjects and that the experiments conformed to the principles set out in the WMA Declaration of Helsinki and the Department of Health and Human Services Belmont Report.	Not Applicable	Statement confirming informed consent is found in a recent publication: Mordmüller et al. A PfSPZ vaccine immunization regimen equally protective against homologous and heterologous controlled human malaria infection. NPJ Vaccines. 2022 doi: 10.1038/s41541-022-00510-z. The manuscript is cited in the respective results and methods sections.
Studies involving **human participants:** For publication of **patient photos**, include a statement confirming that consent to publish was obtained.	Not Applicable	
Studies involving experimental **animals**: State details of **authority granting ethics approval** (IRB or equivalent committee(s), provide reference number for approval. Include a statement of compliance with ethical regulations.	Yes	Materials and Methods Section: Mice were handled in accordance with the German Animal Protection Law (§8 Tierschutzgesetz) and approved by the Landesamt für Gesundheit und Soziales (LAGeSo), Berlin, Germany (project numbers 368/12 and H0335/17).
Studies involving **specimen and field samples:** State if relevant **permits** obtained, provide details of authority approving study; if none were required, explain why.	Not Applicable	

Dual Use Research of Concern (DURC)	Information included in the manuscript?	In which section is the information available?<br>(Reagents and Tools Table, Materials and Methods, Figures, Data Availability Section)
Could your study fall under dual use research restrictions? Please check biosecurity documents and list of select agents and toxins (CDC): https://www.selectagents.gov/sat/list.htm	Not Applicable	
If you used a select agent, is the security level of the lab appropriate and reported in the manuscript?	Not Applicable	
If a study is subject to dual use research of concern regulations, is the name of the **authority granting approval and reference number** for the regulatory approval provided in the manuscript?	Not Applicable	

Reporting

The MDAR framework recommends adoption of discipline-specific guidelines, established and endorsed through community initiatives. Journals have their own policy about requiring specific guidelines and recommendations to complement MDAR.

Adherence to community standards	Information included in the manuscript?	In which section is the information available?<br>(Reagents and Tools Table, Materials and Methods, Figures, Data Availability Section)
State if relevant guidelines or checklists (e.g., **ICMJE, MIBBI, ARRIVE, PRISMA**) have been followed or provided.	Not Applicable	
For **tumor marker prognostic studies,** we recommend that you follow the **REMARK** reporting guidelines (see link list at top right). See author guidelines, under 'Reporting Guidelines'. Please confirm you have followed these guidelines.	Not Applicable	
For **phase II and III randomized controlled trials**, please refer to the **CONSORT** flow diagram (see link list at top right) and submit the CONSORT checklist (see link list at top right) with your submission. See author guidelines, under 'Reporting Guidelines'. Please confirm you have submitted this list.	Not Applicable	

Data Availability

Data availability	Information included in the manuscript?	In which section is the information available?<br>(Reagents and Tools Table, Materials and Methods, Figures, Data Availability Section)
Have **primary datasets** been deposited according to the journal's guidelines (see 'Data Deposition' section) and the respective accession numbers provided in the Data Availability Section?	Yes	Materials and Methods Section: Primary datasets or material generated in this study can be obtained from the corresponding authors under standard transfer agreements. Datasets are deposited on ENA with accession [illegible]
Were **human clinical and genomic datasets** deposited in a public access-controlled repository in accordance to ethical obligations to the patients and to the applicable consent agreement?	Not Applicable	
Are **computational models** that are central and integral to a study available without restrictions in a machine-readable form? Were the relevant accession numbers or links provided?	Not Applicable	
If publicly available data were reused, provide the respective **data citations in the reference list**.	Not Applicable	
